# Supplementary material for: Factors influencing quality nutrition service provision at antenatal care contacts: Findings from a public health facility-based observational study in 21 districts of Bangladesh
Source: PLoS One. 2022 Jan 27;17(1):e0262867. doi: 10.1371/journal.pone.0262867 (PMC8794200; doi:10.1371/journal.pone.0262867)
Supplement: S1 Table — (DOCX) [file pone.0262867.s001.docx]

**S1 Table: Definition of outcome and explanatory variables and data collection methods**

| **Variable type** | **Variable** | **Items/measurements included** | **Data collection methods** |
| --- | --- | --- | --- |
| **Outcome variable** | |  |  |
|  | Quality nutrition service at ANC: Summative score with equal weight of each item (0-4) | - Weight assessed - Anaemia measured - Provided iron-folic acid supplement - Provided nutrition counselling | Structured observation of the ANC consultation |
| **Independent variables** | |  |  |
| ***Facility level*** |  |  |  |
| Facility characteristics and readiness | Type of facility | Type of facility | Structured health facility assessment |
|  | Infrastructural readiness: (1=All five items available) | Availability of:   - Examination room/area - Arrangement for privacy - Examination bed - Electricity - Functioning toilet for clients |  |
|  | Logistics and supplies:  Principal component analysis score of seven items divided into quantile level 1 (low), level 2 (medium), level 3 (good), level 4 (best). | Availability of  -Weighing scale  -Haemoglobin testing tool (tallquist book)  -IFA supplement  -health education materials/visual aids  -ANC card  -ANC guideline  -ANC register |  |
| Facility utilization | - ANC service utilization | - Average number of ANC clients per day |  |
| ***Provider level*** |  |  |  |
| Provider characteristic | - Type of provider - Age - Sex - Duration of work | - Provider’s designation - Age in years - Sex - Years of service at the facility | Interview of health care providers with a structured questionnaire |
|  | - Training on nutrition | - Provider received in-service training on nutrition |  |
| Training and knowledge of health care providers | - Knowledge on nutrition services to be provided during ANC: Summative score of four items (Score= 0-6) | - IFA supplementation (score=1) - Diagnosis and management of anaemia (score=1 - Measurement of weight and height (score =1) - Dietary counselling (score 3)   - protein intake  - green and leafy vegetables  - seasonal fruits |  |
|  | - External supervision | - Service provider received external supervision in last six months |  |
| ***Provider-client interaction*** | - Provider-client interaction: Two indicators were considered individually | - HCP-client communication: services provider informed the client about the progress of the pregnancy or   service provider asked if the client had any question | Structured observation of the ANC consultation |
|  |  | - Service provider used poster, pamphlet, booklets, visual aids or ANC card |  |
| ***Client-level*** |  |  |  |
| Client characteristics | Background characteristics of the clients: Four indicators were considered individually | - Age in years | Exit interviews of the clients (pregnant women) |
|  |  | - Education in years |  |
|  |  | - Gestational age in weeks |  |
|  |  | - Number of ANC visit |  |

ANC: antenatal care, IFA: iron and folic acid
