# Supplementary material for: Factors influencing quality nutrition service provision at antenatal care contacts: Findings from a public health facility-based observational study in 21 districts of Bangladesh
Source: PLoS One. 2022 Jan 27;17(1):e0262867. doi: 10.1371/journal.pone.0262867 (PMC8794200; doi:10.1371/journal.pone.0262867)
Supplement: Attachment — Submitted filename: PONE-D-21-16534 Due 22 Oct.pdf [file pone.0262867.s002.pdf]

PLOS ONE

Determinants of quality nutrition service provision at antenatal care contacts: findings from a public health facility-based observational study in 21 districts of Bangladesh

--Manuscript Draft--

Manuscript Number:	PONE-D-21-16534
Article Type:	Research Article
Full Title:	Determinants of quality nutrition service provision at antenatal care contacts: findings from a public health facility-based observational study in 21 districts of Bangladesh
Short Title:	Determinants of quality nutrition service provision at antenatal care contacts
Corresponding Author:	Sk Masum Billah<br>International Centre for Diarrhoeal Disease Research, Bangladesh<br>Dhaka, BANGLADESH
Keywords:	Quality of Health Care, Maternal Nutrition, Antenatal Care, Job Aids
Abstract:	Malnutrition during pregnancy is associated with increased maternal morbidity and mortality and has a long-term negative impact on child growth and development. Antenatal care (ANC) is the formal point of contact for pregnant women to receive preventive health and nutrition services. We assessed the quality of nutrition service delivery during ANC and examined its determinants related to the health facility, health care provider (HCP) and client characteristics. We conducted a cross-sectional assessment in 179 facilities, including 1242 ANC observations and exit interviews of the pregnant women from 21 districts in Bangladesh. We defined 'quality nutrition service' outcome by adding the counts of four services, including maternal weight measurement, anaemia assessment, nutrition counselling and iron-folic acid (IFA) supplement provision. We explored both the supply side and the client-level determinants of quality nutrition service using multilevel Poisson regression. Overall, only 15% of clients received all four nutrition services. Performance of weight measurement (79%) was higher than IFA provision (56%), anaemia assessment (52%) and nutrition counselling (52%). The multivariable analysis showed that quality nutrition service delivery is positively associated with good logistical readiness of the facilities (aIRR: 1.23, 95% CI: 1.08-1.39), consultation by paramedics (aIRR 1.23, 95% CI: 1.06-1.42) and community health care providers (aIRR 1.32, 95% CI: 1.12-1.57), HCP's knowledge on maternal nutrition (aIRR 1.04; 95% CI: 1.01-1.08), better HCP-client communication (aIRR 1.14; 95% CI: 1.04-1.26) and use visual aids or ANC card (aIRR 1.18; 95% CI: 1.11-1.27). We found limited associations of HCP's training and external supervision with the quality of nutrition services. The quality of nutrition service provision during ANC is suboptimal. Public health nutrition programmers should ensure the facilities' logistical readiness, revisits and reinforce the content and modality of training and supportive supervision of the HCPs. They should also emphasize positive HCP-client communication and use of job aids to improve the quality of nutrition service provision during ANC.
Order of Authors:	Sk Masum Billah
	Nazia Binte Ali
	Abdullah Nurus Salam Khan
	Camille Raynes-Greenow
	Patrick John Kelly
	Md. Shahjahan Siraj
	Sufia Askari
	Purnima Menon
	Shams El Arifeen
	Michael John Dibley
	Phuong Hong Nguyen

Powered by Editorial Manager® and ProduXion Manager® from Aries Systems Corporation

Additional Information:	
**Question**	**Response**
**Financial Disclosure**<br><br>Enter a financial disclosure statement that describes the sources of funding for the work included in this submission. Review the submission guidelines for detailed requirements. View published research articles from *PLOS ONE* for specific examples.<br><br>This statement is required for submission and **will appear in the published article** if the submission is accepted. Please make sure it is accurate.<br><br>**Unfunded studies**<br>Enter: *The author(s) received no specific funding for this work.*<br><br>**Funded studies**<br>Enter a statement with the following details:<br>• Initials of the authors who received each award<br>• Grant numbers awarded to each author<br>• The full name of each funder<br>• URL of each funder website<br>• Did the sponsors or funders play any role in the study design, data collection and analysis, decision to publish, or preparation of the manuscript?<br>• **NO** - Include this sentence at the end of your statement: *The funders had no role in study design, data collection and analysis, decision to publish, or preparation of the manuscript.*<br>• **YES** - Specify the role(s) played.<br><br>* typeset	The lead author, SMB, received funding from Children's Investment Fund Foundation (CIFF) for conducting the initial assessment for the Evaluation of Accelerating Implementation of the National Nutritional Service (AINNS) project in Bangladesh (Grant number G-1612-00805). This paper uses the data from the initial assessment. Sufia Askari from the funding agency has contributed to the conception of the paper and provided inputs to the manuscript draft. The funding agency had no other role in original study design, data collection and analysis, decision to publish, or preparation of the manuscript.
**Competing Interests**<br><br>Use the instructions below to enter a competing interest statement for this submission. On behalf of all authors, disclose any competing interests that could be perceived to bias this work—acknowledging all financial support and any other relevant financial or non-financial competing interests.	I have read the journal's policy and the authors of this manuscript have the following competing interests: Sufia Askari is from the funding agency. She contributed to the study concept but played no role in study design, data collection or data analysis. Her contributions to this manuscript included inputs to the section on interpretation of results and review of manuscript drafts. However, the final decision about the results to include, interpretation and conclusion rested with the lead author and the authors from the evaluation team. All other authors declare that no competing interests exist.

This statement is required for submission and **will appear in the published article** if the submission is accepted. Please make sure it is accurate and that any funding sources listed in your Funding Information later in the submission form are also declared in your Financial Disclosure statement.<br><br>View published research articles from *PLOS ONE* for specific examples.<br><br>**NO authors have competing interests**<br><br>Enter: *The authors have declared that no competing interests exist.*<br><br>**Authors with competing interests**<br><br>Enter competing interest details beginning with this statement:<br><br>*I have read the journal's policy and the authors of this manuscript have the following competing interests: [insert competing interests here]*<br><br>* typeset	
**Ethics Statement**<br><br>Enter an ethics statement for this submission. This statement is required if the study involved:<br><br>• Human participants<br>• Human specimens or tissue<br>• Vertebrate animals or cephalopods<br>• Vertebrate embryos or tissues<br>• Field research<br><br>Write "N/A" if the submission does not require an ethics statement.<br><br>General guidance is provided below. Consult the submission guidelines for detailed instructions. **Make sure that all information entered here is included in the**	Ethical Review Committee of icddr,b approved the study (Protocol Number-15107). Written informed consent was obtained from health care providers, health facility managers and clients before data collection.

**Methods section of the manuscript.**<br><br>**Format for specific study types**<br><br>**Human Subject Research (involving human participants and/or tissue)**<br>• Give the name of the institutional review board or ethics committee that approved the study<br>• Include the approval number and/or a statement indicating approval of this research<br>• Indicate the form of consent obtained (written/oral) or the reason that consent was not obtained (e.g. the data were analyzed anonymously)<br><br>**Animal Research (involving vertebrate animals, embryos or tissues)**<br>• Provide the name of the Institutional Animal Care and Use Committee (IACUC) or other relevant ethics board that reviewed the study protocol, and indicate whether they approved this research or granted a formal waiver of ethical approval<br>• Include an approval number if one was obtained<br>• If the study involved *non-human primates*, add *additional details* about animal welfare and steps taken to ameliorate suffering<br>• If anesthesia, euthanasia, or any kind of animal sacrifice is part of the study, include briefly which substances and/or methods were applied<br><br>**Field Research**<br><br>Include the following details if this study involves the collection of plant, animal, or other materials from a natural setting:<br>• Field permit number<br>• Name of the institution or relevant body that granted permission	
**Data Availability**<br><br>Authors are required to make all data underlying the findings described fully available, without restriction, and from the time of publication. PLOS allows rare exceptions to address legal and ethical concerns. See the PLOS Data Policy and FAQ for detailed information.	Yes - all data are fully available without restriction

Powered by Editorial Manager® and ProduXion Manager® from Aries Systems Corporation

A Data Availability Statement describing where the data can be found is required at submission. Your answers to this question constitute the Data Availability Statement and **will be published in the article**, if accepted.<br><br>**Important:** Stating 'data available on request from the author' is not sufficient. If your data are only available upon request, select 'No' for the first question and explain your exceptional situation in the text box.<br><br>Do the authors confirm that all data underlying the findings described in their manuscript are fully available without restriction?	
**Describe where the data may be found in full sentences. If you are copying our sample text, replace any instances of XXX with the appropriate details.**<br><br>• If the data are **held or will be held in a public repository**, include URLs, accession numbers or DOIs. If this information will only be available after acceptance, indicate this by ticking the box below. For example: *All XXX files are available from the XXX database (accession number(s) XXX, XXX.*).<br>• If the data are all contained **within the manuscript and/or Supporting Information files**, enter the following: *All relevant data are within the manuscript and its Supporting Information files.*<br>• If neither of these applies but you are able to provide **details of access elsewhere**, with or without limitations, please do so. For example:<br><br>*Data cannot be shared publicly because of [XXX]. Data are available from the XXX Institutional Data Access / Ethics Committee (contact via XXX) for researchers who meet the criteria for access to confidential data.*<br><br>*The data underlying the results*	All relevant data are held on a public repository and can be accessed from https://doi.org/10.6084/m9.figshare.14608206.v1

presented in the study are available from (include the name of the third party and contact information or URL).<br>• This text is appropriate if the data are owned by a third party and authors do not have permission to share the data.<br><br>* typeset	
Additional data availability information:	

Powered by Editorial Manager® and ProduXion Manager® from Aries Systems Corporation

Manuscript Click here to access/download;Manuscript;Manuscript.docx

Determinants of quality nutrition service provision at antenatal care contacts: findings from a public health facility-based observational study in 21 districts of Bangladesh

Authors

Sk Masum Billah[1,2*], Nazia Binte Ali[1], Abdullah Nurus Salam Khan[1], Camille Raynes-Greenow[2], Patrick John Kelly[2], Md. Shahjahan Siraj[1], Sufia Askari[3], Purnima Menon[4], Shams El Arifeen[1], Michael John Dibley[2], Phuong Hong Nguyen[4]

[1]Maternal and Child Health Division, International Centre for Diarrhoeal Disease Research, Bangladesh (icddr,b), Dhaka, Bangladesh
[2]The University of Sydney School of Public Health, Sydney, NSW 2006, Australia
[3]Children's Investment Fund Foundation, Clifford Street. London W1S 2FT, UK
[4]International Food Policy Research Institute (IFPRI), Washington, DC., USA

* Corresponding author
E-mail: billah@icddrb.org

Abstract

Malnutrition during pregnancy is associated with increased maternal morbidity and mortality and has a long-term negative impact on child growth and development. Antenatal care (ANC) is the formal point of contact for pregnant women to receive preventive health and nutrition services. We assessed the quality of nutrition service delivery during ANC and examined its determinants related to the health facility, health care provider (HCP) and client characteristics. We conducted a cross-sectional assessment in 179 facilities, including 1242 ANC observations and exit interviews of the pregnant women from 21 districts in Bangladesh. We defined 'quality nutrition service' outcome by adding the counts of four services, including maternal weight measurement, anaemia assessment, nutrition counselling and iron-folic acid (IFA) supplement provision. We explored both the supply side and the client-level determinants of quality nutrition service using multilevel Poisson regression. Overall, only 15% of clients received all four nutrition services. Performance of weight measurement (79%) was higher than IFA provision (56%), anaemia assessment (52%) and nutrition counselling (52%). The multivariable analysis showed that quality nutrition service delivery is positively associated with good logistical readiness of the facilities (aIRR: 1.23, 95% CI: 1.08-1.39), consultation by paramedics (aIRR 1.23, 95% CI: 1.06-1.42) and community health care providers (aIRR 1.32, 95% CI: 1.12-1.57), HCP's knowledge on maternal nutrition (aIRR 1.04; 95% CI: 1.01-1.08), better HCP-client communication (aIRR 1.14; 95% CI: 1.04-1.26) and use visual aids or ANC card (aIRR 1.18; 95% CI: 1.11-1.27). We found limited associations of HCP's training and external supervision with the quality of nutrition services. The quality of nutrition service provision during ANC is suboptimal. Public health nutrition programmers should ensure the facilities' logistical readiness, revisits and reinforce the content and modality of training and supportive supervision of the HCPs. They should also emphasize positive HCP-client communication and use of job aids to improve the quality of nutrition service provision during ANC.

Introduction

Maternal nutrition is a major global public health concern. Undernutrition and micronutrient deficiencies during pregnancy are associated with increased maternal morbidity and mortality and have a long term negative impact on child growth and development [1, 2]. Despite gradual progress in low and middle-income countries (LMIC) during the last two decades, maternal and child nutrition improvements remain an unfinished agenda, especially with the interruptions by the COVID-19 pandemic [1]. In Bangladesh, 42% of women of reproductive age (15-49 years) and nearly half of all pregnant women are anaemic, 12% are thin, and 14% have short stature [3, 4]. The principal drivers for maternal malnutrition are poor-quality diets, low intake of micronutrient supplements, and lack of access to quality health care during pregnancy [5, 6]. A comprehensive analysis of data from 69 LMICs suggested that receiving antenatal care (ANC) from appropriate sources and with recommended frequency was associated with positive birth outcomes and childhood nutrition [7]. Evidence also suggests receiving three or more antenatal care visits is likely to reduce maternal malnutrition by three-fold[8]. All these findings highlight the need for improving coverage and quality of nutrition services during pregnancy.

ANC is the first contact for pregnant women to health and nutrition services. The "WHO antenatal care guideline for positive pregnancy experience" recommends routine supplementation of iron-folic acid (IFA), nutrition counselling, and weight monitoring during ANC to ensure greater coverage of nutrition interventions during pregnancy [9]. Aligned with global recommendations, the government of Bangladesh prioritized maternal nutrition service delivery using the ANC platform in their fourth Health Nutrition and Population Sector Program to continue the 'nutrition mainstreaming' into the health systems approach [10]. Like several LMICs, timely utilization and quality of ANC remain a major challenge to increase the reach and coverage of nutrition interventions in Bangladesh [11]. In 2017, the coverage of four ANC visits was 47%, indicating more than half of women were missing the opportunity of maternal nutrition services

[4]. Another challenge is ensuring the quality of care, without which programmes will not fully harness the benefits of the nutrition interventions. Moreover, facilities have demonstrated incomplete readiness for nutrition interventions during ANC (51%) [12]. Nutrition input-adjusted coverage was suboptimal (18% for ANC) and disproportionately affected the poor and women low education. Suboptimal quality of care for nutrition services during ANC further deteriorates the situation. An assessment of the quality of nutrition service in the primary care facilities showed that only 30% of mothers received four nutrition services during ANC [13].

The quality of health service delivery primarily depends on three-levels of factors: structure (readiness of the health facilities), process (provision and experience of care), and outcomes (effective coverage and health outcomes) [14]. Previous studies from LMICs have mainly reported the quality of ANC service by using one or some of the following indicators: frequency of visits, structural readiness to provide the service, content on maternity care, client experience, and their determinants [11, 15-21]. Although, most of these studies covered the structural and outcome level indicators, very few explored process level indicators. One of the few attempts in exploring process-level factors and adherence to recommended quality provisions during ANC was made in 2016 in Tanzania [21]. The study reported that female providers, availability of routine tests and basic medicines at the public facilities and providers who received refresher training were positively associated with better adherence to quality ANC provision at a single point of contact. However, this study lacks the client level factor and experience of care domains. On the contrary, another study conducted in Ghana explored the client level factors for quality ANC but lacked structural and provider-level factors [22]. Moreover, a recent systematic review (2018) looking at the effectiveness of nutrition programming, highlighted the need for additional analysis on barriers and enablers of nutrition service delivery using ANC and maternal, newborn and child health platforms in South Asia [23]. Considering the existing evidence and current research needs, a comprehensive analysis of quality ANC service delivery including structural, process and client level factors, will be beneficial for

effective programme planning [21]. This paper aims to assess the quality nutrition service provision during ANC and examine its determinants related to facility readiness, health care providers, the process of care, and client characteristics.

Methods

Study design and setting

This study is a part of an initial cross-sectional assessment of coverage and quality of maternal and child nutrition services for a health system strengthening project that aimed to accelerate the implementation of the National Nutritional Service (AINNS) in Bangladesh. The project included interventions to improve coordination of nutrition service delivery both at the national and sub-national levels and health care provider's skills for maternal and child nutrition service delivery at public health care platforms. Details of the project evaluation plan have been reported elsewhere [24, 25]. Briefly, the initial assessment was done in 21 out of 64 districts in Bangladesh. We selected public health facilities from 14 of 40 AINNS intervention districts and 7 of 24 non-intervention districts. Before selection, we matched two intervention districts with one non-intervention district by a score created by principal component analysis of district's population, geographic, and health care characteristics. The initial assessment included secondary to community-level public health facilities, namely district hospitals, sub-district hospitals, union health and family welfare centres and community clinics at ward level. At district and sub-district level hospitals, physicians with or without specialization in obstetrics and gynaecology, nurses and paramedics provide maternal care services, including ANC. At union level facilities, which serves a catchment of 25-30 thousand people, paramedics are the main ANC service providers. These paramedics have either a four-year medical diploma or 18 months of pre-service training including six months on midwifery skills. At community clinics, which serve six to eight thousand people in each ward, community health care providers and frontline community health workers provide ANC services. From each district

we selected the districts hospital and the sub-district hospital from two randomly selected subdistricts for this assessment. We also randomly selected four union-level health facilities and four community clinics from a list of functioning facilities prepared in consultation with the sub-district health manager in each chosen sub-district. We conducted the initial assessment between February and June 2016.

Data collection

We used the structure and process domains of the Donabedian framework for the determinants of quality nutrition service provision. Globally, the Donabedian framework is widely used for measuring the quality of healthcare service delivery [14, 26]. This framework identifies structure, process, and outcome level indicators for a comprehensive measurement of quality of care [26, 27]. We have also considered the client characteristics as they have been found to influence the provision of ANC services [21]. We used mixed methods to collect different data at both supply and client levels, including 1) a health facility readiness assessment, 2) health care provider interviews, 3) direct observation of ANC practices, and 4) exit interviews with the clients. For health facility assessment, we used a structured checklist adapted from the Bangladesh Health Facility Assessment tool [28] to collect information on facility infrastructure, and availability of specific logistics items (including weighing scale, haemoglobin testing tool, health education material/visual aids, ANC card, guideline, registers, and iron-folic acid (IFA) supplements). Interviews of the ANC service providers included information about their duration of work in the facility, training status, and knowledge regarding nutrition services. For direct observation, we developed the observation checklist following the standard operating procedures for maternal health services [29] to document the nutrition services provided at ANC consultation. Finally, we conducted exit interviews with clients after receiving ANC to collect information on their background, demographic and obstetric characteristics, and ANC service care-seeking. S1 Table summarizes the outcome and explanatory measures, definitions, and data collection methods.

Sample size

We conducted the assessment in 231 facilities, and in 184 facilities, we observed ANC services provided on the assessment days. We interviewed 217 health care providers who offered ANC services at the facilities on the day of the visit, and we observed 1296 ANC consultations. We excluded 54 observations due to missing information about the health care providers, the client's characteristics, or the exit interview (Fig 1). We conducted the final analysis among 1242 observations of ANC services provided by 201 service providers at 179 facilities.

Fig 1. Study flow diagram showing health facilities, health care providers and participants recruited and final sample size.

ANC: antenatal care, HCP: Health care provider, HF: Health facility

Measures of quality of nutrition services (Outcome measures)

We considered maternal weight measurement, assessment for anaemia, nutrition counselling and provision of IFA supplements as the ANC nutrition services [10]. We included anaemia assessment in the measurement of the quality of nutrition service. It an essential service during ANC, following the national guideline for a population with a high anaemia burden among women of reproductive age [30]. During the observations of ANC, the assessors recorded the services provided. We created a composite score variable for 'quality nutrition service' by adding the number of services (out of four) provided at the ANC consultation. Similar methods of defining a composite score for quality service have been used in previous studies [11, 31].

Explanatory variables

We explored the supply side and the client-level determinants of quality nutrition service provided at ANC (S1 Table). According to the 'structure' domain of the Donabedian framework [14], we considered the

health facility characteristics and readiness, and HCP's characteristics and knowledge as supply-side determinants. At the health facility level, we constructed two indicators. The first was structural readiness (if all five readiness items were available). And the second was logistics and supplies readiness (created from seven logistic and supplies items using a principal component analysis, then categorized into four logistical readiness quantiles). Cronbach's alpha for the logistics and supplies included in the composite score calculation was 0.62. HCP's characteristics included age, sex, duration of service at the facility, and receiving external supervision at least once in the last six months. HCP's knowledge of nutrition service at ANC was a composite score derived by adding the correct responses to four questions (S1 Table). The 'process' level determinants two variables on HCP-client interaction including HCP-client communication (HCP discussing the progress of pregnancy and asking if the client has any question) and use of visual aids and ANC card during ANC service provision. Client-level determinants involved client characteristics namely age, education, gestational age, and the number of ANC visits.

Statistical analysis

We used descriptive analysis to summarise the client and provider characteristics, health facility readiness to provide nutrition services at ANC, and client-provider interactions during ANC consultation. We reported summary statistics by frequency and proportion or by mean (±SD) for categorical and continuous variables, respectively. We explored the differences in provider characteristics and provider-client interactions by types of provider and facility readiness by types of facilities using ANOVA and chi-square test for continuous and categorical characteristics variables, respectively. We checked for the linearity in the relationship between each continuous explanatory variable and the quality nutrition service score by fitting the locally estimated scatterplot smoothing (loess) curve. As the outcome variable, includes counts of nutrition services,, we used Poisson regressions to examine the potential determinants of quality nutrition service at ANC [32]. We included provider and facility as the random effects in the model as

clients were nested within the providers and providers were nested within the facilities. We calculated p-values using Wald tests that had robust standard errors to adjust for model violations, such as under-dispersion. In the multivariable regression model, we included the explanatory variables associated with quality nutrition service at a p-value<0.2 in the bivariate analysis. The facility type had collinearity (correlation coefficient 0.72) with HCP type. We compared Akaike's information criterion (AIC) and Bayesian information criterion (BIC) between the models with and without facility type and excluded facility type from the final model. We also ran multivariable Poisson regression using each of the four elements of quality nutrition services as the outcome. We reported the adjusted incidence rate ratios (aIRR) and their 95% confidence interval as the measure of association. Associations were considered statistically significant at a p-value<0.05 in the final model. We used Stata version 14 in all statistical analyses.

Ethical approval

We obtained ethical approval from the Ethical Review Committee of icddr,b (Protocol Number-15107), and written informed consent from HCPs, health facility managers and clients before data collection.

Result

The study includes data from 21 district hospitals, 40 sub-district hospitals, 61 union level facilities and 57 community clinics. Most of the facilities (88%) had one HCP who provided ANC service on the visit day and participated in the study. Overall, 58% of facilities had all five infrastructural readiness items considered in this analysis (Table 1). Infrastructural readiness of community clinics (21%) and FWC (56%) were poorer than higher-tier facilities, including district hospitals (95%) and sub-district hospitals (91%). The availability of logistics like weighing scale (82%), ANC register (99%), IFA supplements (93%) and visual aids for counselling (88%) were high. However, only 35% of facilities had a haemoglobin testing kit to detect

maternal anaemia and about a third had an ANC guideline. We found significant differences in the availability of haemoglobin testing tool, visual job aids, ANC card and IFA supplement across different types of facilities (all p<0.01).

Table 1. Characteristics and readiness of the healthcare facilities by types of facility

	Overall (N=179)	District hospital (N=21)	Sub-district hospital (N=40)	Union health centre (N=61)	Community clinic (N=57)	p-value
**All structural readiness[1] items available**	57.5	90.5	95.0	55.7	21.0	<0.001
**Logistics and supplies**						
Weighing scale	82.1	81.0	72.5	85.3	86.0	0.318
Haemoglobin testing tool	35.2	9.5	17.5	50.8	40.4	<0.001
Health education materials/visual aids	88.3	81.0	72.5	93.4	96.5	0.001
ANC card	84.9	71.4	72.5	95.1	87.7	0.004
ANC guideline	30.2	38.1	30.0	32.8	24.6	0.642
ANC register	99.4	95.2	100	100	100	0.056
IFA supplement	93.3	95.2	97.5	83.6	100	0.002
**ANC utilization per day**						
<5	44.7	9.5	42.5	57.4	45.6	
5-10	35.8	23.8	30.0	36.1	43.9	<0.001
11 or more	19.6	66.7	27.5	6.6	10.5	

Values are %; p-values are test of difference in facility readiness across facility types; [1] Structural readiness: availability of examination room/area, privacy, examination bed, electricity and functioning toilet for clients
ANC: antenatal care, IFA: iron and folic acid

Most of the HCPs (86%) were female (Table 2). About a half of the community HCPs received nutrition training compared to only 9% of physicians. Knowledge of nutrition services was similar across the HCP types. Although 84% HCPs received external supervision at least once in six months preceding the assessment, the external supervision of nurses was much lower (55%) compared to other types of HCP (>80%). HCP-client interaction during ANC consultation varied significantly by types of providers (Table 2). While the proportion of HCPs informing clients about the progress of their pregnancy or allowing clients to ask questions was high (73%), visual aids or ANC card usage during counselling was low (only in 47% ANC consultations). On average, clients who received ANC from the facilities were 23 years old and

had eight years of schooling. Clients were mostly first-time pregnant women (42%) in the second trimester (46%) and came for either the second or third ANC visit (44%) (data not shown).

Table 2. Characteristics of health care providers and provider-client interaction by types of health care provider

	Overall (N=201)	Physician (N=35)	Nurse (N=20)	Paramedics (N=88)	CHCP/CHW (N=58)	p-value
**HCP characteristics**						
Age of provider (years)	38.1 (±10.0)	35.7 (±6.8)	41.4 (±9.1)	43.1 (±10.2)	30.9 (±6.2)	<0.001
Sex of the provider						
Male	13.9	5.7	0	4.6	37.9	<0.001
Female	86.1	94.3	100	95.5	62.1	
Duration of work at the current facility (years)						
≤3	32.3	62.9	35.0	35.2	8.6	<0.001
4-7	37.8	25.7	15.0	15.9	86.2	
8 or more	29.9	11.4	50.0	48.9	5.2	
Received any in-service training on nutrition	28.9	8.6	20.0	23.9	51.7	<0.001
Knowledge (score out of 6) on nutrition services at ANC[1]	4.3 (±1.4)	4.2 (±1.6)	4.3 (±1.1)	4.3 (±1.4)	4.3 (±1.4)	0.994
Received any external supervision in last 6 months	83.6	82.9	55.0	83.0	94.8	0.001
Place of work						
District hospital	13.9	40.0	30.0	9.1	0	<0.001
Sub-district hospital	26.9	60.0	70.0	21.6	0	
Union health centre	30.4	0	0	69.3	0	
Community clinic	28.9	0	0	0	100	
**HCP-client interaction**	**N=1242**	**N=338**	**N=179**	**N=497**	**N=228**	
HCP-client communication[2]	72.6	61.8	63.1	87.7	63.2	<0.001
Used visual aids or ANC card	47.0	29.9	71.5	56.3	32.9	<0.001

Values are % or mean (± SD) , p-values test difference of health care providers characteristics by types of health care provider, [1]includes knowledge on the provision of Iron folic acid, diagnosis and management of anaemia, weight measurement and diet counselling; [2]HCP discussing about the progress of pregnancy, asked if the client had any question HPC: health care providers, ANC: antenatal care, CHCP: Community Health Care Providers, CHW: Community Health Workers

Among the four nutrition services to be provided during ANC, assessment of weight for maternal weight gain during pregnancy was the highest (79%), followed by IFA provision (56%) (Fig 2). In nearly half of the ANC consultations, HCPs assessed anaemia either by testing haemoglobin or by clinical examination of

eye or palm and provided counselling on appropriate dietary practices. Overall, only 15% of clients received all four nutrition services components and about a third received three services (Fig 2B).

Fig 2. Percentage of clients received (A) nutrition service components and (B) number of nutrition service components at ANC.

IFA: Iron folic acid

We summarized the determinants of the overall quality of ANC nutrition services in Table 3. The unadjusted results showed that logistical readiness of the facilities, types of HCP, knowledge of the HCP on maternal nutrition, provision for external supervision, HCP-client communication, use of visual aids or ANC cards during the consultation, and gestational age at ANC visit were positively associated with quality nutrition service at ANC. In the adjusted analysis, there was an association between facilities with medium and good logistical readiness with higher quality nutrition service provision (adjusted incidence rate ratio, aIRR: 1.17, 95% CI: 1.04-1.32 and aIRR: 1.23, 95% CI: 1.08-1.39, respectively) compared to facilities with low readiness. Although nurses provided services with similar quality to the physicians, paramedics were 23% (aIRR 1.23, 95% CI: 1.06-1.42) and community health care providers 32% (aIRR 1.32, 95% CI: 1.12-1.57) more likely to provide quality nutrition services. Each point increase in the HCPs' knowledge of nutrition was associated with a 4% higher quality of nutrition services at ANC (aIRR 1.04; 95% CI: 1.01-1.08). There was no association between HCP's training on nutrition and receiving external supervision in the last six months with quality nutrition service provision in the adjusted analysis. There was an association between HCP-client communication (aIRR 1.14; 95% CI: 1.04-1.26) and the use of visual aids or ANC cards (aIRR 1.18; 95% CI: 1.11-1.27) with quality nutrition service provision. Finally, the client's in the second and third trimesters of pregnancy received higher-quality nutrition service (aIRR: 1.23, 95% CI: 1.11-1.35 and aIRR 1.19, 95% CI: 1.08-1.32, respectively) compared with those in the first trimester.

Table 3. Unadjusted and adjusted incidence rate ratio of quality nutrition service at ANC according to health facility readiness, HCP characteristics, client-provider interactions and client characteristics

Variables	Unadjusted models		Adjusted model	
	IRR (95% CI)	p-value	aIRR (95% CI)	p-value
**Facility characteristics and readiness**				
All structural readiness[1] available	0.86 (0.77-0.96)	0.007	1.00 (0.98-1.03)	0.884
Logistical readiness quantile[2] (ref: Level 1 (low))		0.015		0.013
Level 2 (medium)	1.23 (1.04-1.45)	0.016	1.17 (1.04-1.32)	0.011
Level 3 (good)	1.31 (1.11-1.54)	0.001	1.23 (1.08-1.39)	0.002
Level 4 (best)	1.27 (1.03-1.58)	0.024	1.10 (0.95-1.28)	0.201
ANC utilization per day (ref: <5)		0.107		0.302
5-10	0.92 (0.81-1.05)	0.220	0.98 (0.89-1.07)	0.614
11 or more	0.85 (0.73-0.99)	0.039	0.91 (0.82-1.02)	0.123
**Provider characteristics**				
Age of provider (years)	1.00 (0.99-1.00)	0.447		
Sex of provider (ref: female)	0.99 (0.84-1.17)	0.919		
Duration of work at this facility (years) (ref: ≤3)		0.062		0.731
4-7	1.16 (1.02-1.32)	0.024	1.04 (0.93-1.16)	0.537
8 or more	1.13 (1.00-1.27)	0.055	1.05 (0.93-1.18)	0.464
Type of provider (ref: Physician)		<0.001		<0.001
Nurse	0.99 (0.80-1.21)	0.900	0.89 (0.74-1.05)	0.169
Paramedic	1.41 (1.17-1.69)	<0.001	1.23 (1.06-1.42)	0.006
CHCP/CHW	1.46 (1.21-1.76)	<0.001	1.32 (1.12-1.57)	0.001
Received any training on nutrition	1.11 (1.00-1.24)	0.060	1.00 (0.91-1.10)	0.949
Knowledge on nutrition service (score out of 6)	1.07 (1.03-1.11)	0.001	1.04 (1.01-1.08)	0.012
Received external supervision in last 6 months	1.17 (1.01-1.36)	0.034	1.09 (0.97-1.24)	0.154
**HCP-client interaction (process)**				
HCP-client communication[3]	1.24 (1.11-1.38)	<0.001	1.14 (1.04-1.26)	0.007
Used visual aids or ANC card	1.19 (1.10-1.28)	<0.001	1.18 (1.11-1.27)	<0.001
**Client characteristics**				
Age of women in years	1.00 (0.99-1.00)	0.169	1.00 (0.99-1.01)	0.946
Education (years of schooling)	1.00 (0.99-1.01)	0.781		
Gravida (ref: 1)		0.184		0.188
2	1.01 (0.96-1.05)	0.784	1.00 (0.96-1.05)	0.841
3 or more	0.95 (0.90-1.01)	0.127	0.94 (0.87-1.02)	0.149
Gestational Age (ref: 1st trimester)		<0.001		<0.001
2nd trimester	1.28 (1.16-1.42)	<0.001	1.23 (1.11-1.35)	<0.001
3rd Trimester	1.23 (1.10-1.37)	<0.001	1.19 (1.08-1.32)	0.001
Number of ANC Visit (ref: 1st visit)		0.234		
2nd – 3rd	1.05 (0.99-1.11)	0.108		
4th or more	1.04 (0.99-1.09)	0.156		

[1] Structural readiness: availability of examination room, privacy, examination bed, electricity and functioning toilet for clients; [2]Logistical readiness quantile was derived by categorizing score from principal component analysis of the availability of weighing scale, haemoglobin testing tool, health education materials/visual aids, ANC card, ANC guideline, ANC register and IFA supplement; [3] HCP discussing about the progress of pregnancy, asked if the client

had any question; IRR: incidence rate ratio, aIRR: adjusted incidence rate ratio; ANC: antenatal care, CHCP: Community Health Care Providers, CHW: Community Health Workers; HCP: Health care provider, ref: reference

We also explored facility, HCP and client level determinants for each of the components of nutrition service at ANC (Table 4). The key determinants for weight measurement were the logistical readiness of the health facilities, lower level providers like paramedics or CHCP/CHW, using visual aids or ANC cards during service provision, and clients attending health facilities in the second and third trimester. Anaemia assessment was also positively associated with higher knowledge on nutrition and the use of visual aids or ANC cards but inversely associated with provider receiving external supervision. For IFA provision, the key determinants include lower level HCPs like community health workers, clients in the second and third trimester of pregnancy, external supervision in the last six months, and HCP-client communication. Provision of nutrition counselling was lower at facilities with 11 or more ANC utilization daily, but higher among community level providers and HCPs with better knowledge of nutrition service. The use of visual aids or ANC cards and good HCP-client communication during the consultation also resulted in the higher nutrition counselling at ANC.

Table 4. Adjusted incidence rate ratio of four nutrition service components at ANC according to health facility readiness, HCP characteristics, client-provider interactions and client characteristics

Variables	Weight measured		Anaemia[1] assessed		IFA provided		Nutrition counselling	
	aIRR (95% CI)	p-value	aIRR (95% CI)	p-value	aIRR (95% CI)	p-value	aIRR (95% CI)	p-value
**Facility characteristics**								
All structural readiness[1] available	1.00 (0.97-1.04)	0.876	1.03 (0.96-1.10)	0.483	0.97 (0.91-1.04)	0.383	1.00 (0.94-1.06)	0.996
Logistical readiness quantile[3] (ref: Level 1 -low)		0.002		0.386		0.410		0.236
Level 2 (medium)	1.61 (1.22-2.12)	0.001	0.94 (0.67-1.33)	0.749	0.83 (0.61-1.13)	0.253	1.29 (0.96-1.75)	0.095
Level 3 (good)	1.59 (1.21-2.11)	0.001	1.18 (0.87-1.60)	0.276	0.81 (0.62-1.05)	0.108	1.35 (1.00-1.82)	0.047
Level 4 (best)	1.39 (1.03-1.87)	0.030	0.94 (0.63-1.40)	0.761	0.84 (0.61-1.17)	0.306	1.25 (0.90-1.74)	0.190
ANC utilization per day (ref: <5)		0.699		0.163		0.799		0.045
5-10	1.00 (0.86-1.16)	0.999	1.06 (0.81-1.38)	0.668	0.93 (0.72-1.18)	0.538	0.89 (0.72-1.10)	0.271
11 or more	1.06 (0.92-1.23)	0.456	0.78 (0.57-1.07)	0.130	1.01 (0.73-1.39)	0.950	0.70 (0.53-0.94)	0.016
**Provider characteristics**								
Duration of work in facility (years) (ref: ≤3)		0.486		0.334		0.454		0.471
4-7	1.07 (0.90-1.28)	0.429	1.17 (0.87-1.57)	0.298	0.81 (0.58-1.14)	0.235	1.15 (0.88-1.51)	0.302
8 or more	1.11 (0.94-1.30)	0.212	0.95 (0.73-1.24)	0.712	0.94 (0.68-1.33)	0.779	1.18 (0.89-1.56)	0.260
Type of provider (ref: Physician)		0.001		0.132		0.023		0.029
Nurse	0.94 (0.76-1.18)	0.615	0.77 (0.51-1.17)	0.222	0.87 (0.51-1.46)	0.594	0.88 (0.57-1.38)	0.586
Paramedic	1.23 (1.01-1.49)	0.042	1.07 (0.80-1.43)	0.658	1.41 (0.99-2.00)	0.058	1.28 (0.89-1.85)	0.182
CHCP/CHW	1.32 (0.99-1.74)	0.055	0.76 (0.50-1.16)	0.202	1.74 (1.19-2.56)	0.005	1.56 (1.03-2.34)	0.034
Received training on nutrition	0.95 (0.85-1.06)	0.327	1.02 (0.76-1.37)	0.882	1.11(0.88-1.40)	0.357	1.01(0.85-1.20)	0.886
Knowledge on nutrition service (score out of 6)	0.98 (0.93-1.03)	0.404	1.18 (1.08-1.29)	<0.001	0.99 (0.91-1.07)	0.745	1.08(1.01-1.16)	0.040
Received external supervision in last 6 months	1.00 (0.86-1.17)	0.959	0.77 (0.60-0.99)	0.042	2.42(1.42-4.11)	0.001	1.00(0.75-1.31)	0.949
**HCP-client interaction (process)**								
HCP-client communication[4]	1.06 (0.92-1.20)	0.427	0.87 (0.69-1.09)	0.222	1.34 (1.06-1.70)	0.013	1.51 (1.17-1.95)	0.002
Used visual aids or ANC card	1.23 (1.11-1.37)	<0.001	1.28 (1.08-1.53)	0.005	1.05 (0.90-1.22)	0.571	1.20 (1.01-1.42)	0.038
**Client characteristics**								
Age of women (in years)	1.01(1.00-1.01)	0.144	1.00 (0.98-1.01)	0.546	1.00(0.99-1.01)	0.955	1.00(0.98-1.01)	0.675
Gravida (ref: 1)		0.084		0.927		0.239		0.432
2	1.02 (0.96-1.08)	0.546	0.99 (0.88-1.13)	0.907	1.06 (0.96-1.15)	0.245	0.96 (0.87-1.06)	0.443
3+	0.92 (0.82-1.02)	0.120	1.03 (0.87-1.20)	0.790	0.96 (0.84-1.11)	0.599	0.90 (0.76-1.06)	0.219

Variables	Weight measured		Anaemia[1] assessed		IFA provided		Nutrition counselling	
	aIRR (95% CI)	p-value	aIRR (95% CI)	p-value	aIRR (95% CI)	p-value	aIRR (95% CI)	p-value
Gestational Age (ref: 1st trimester)		0.026		0.128		<0.001		0.360
2nd trimester	1.19 (1.05-1.33)	0.005	1.03 (0.85-1.25)	0.763	2.13 (1.58-2.88)	<0.001	0.92 (0.79-1.09)	0.354
3rd Trimester	1.19 (1.04-1.35)	0.009	1.14 (0.93-1.38)	0.206	1.84 (1.35-2.51)	<0.001	0.89 (0.75-1.05)	0.164

[1]Anaemia assessment included assessment of blood haemoglobin level or examined eye or palm (clinical assessment), [2] Structural readiness: availability of examination room/area, privacy, examination bed, electricity and functioning toilet for clients**;** [3]Logistical readiness quantile was derived by categorizing score from principal component analysis of the availability of weighing scale, haemoglobin testing tool, health education materials/visual aids, ANC card, ANC guideline, ANC register and IFA supplement. [4] HCP discussing about the progress of pregnancy, asked if the client had any question, CHCP: Community Health Care Providers, CHW: Community Health Workers; HCP: Health care provider, aIRR: adjusted incidence rate ratio

Discussion

Antenatal care is the formal point of contact for pregnant women to receive preventive health and nutrition services. We found that only 15% of women received all four nutrition services at ANC. Anaemia measurement and nutrition counselling were the least provided (52%) nutrition service. Logistical readiness of the facilities, types of ANC provider, knowledge of nutrition services, client-provider interactions during the consultation, and gestational age were determinants of quality nutrition services during ANC. We found limited associations of training and external supervision of the providers with the quality of nutrition services. Our study adds to the knowledge on the quality of nutrition service provision during pregnancy. It provides some critical insights on areas to intervene for improving the nutrition service provision at ANC in a low resource setting.

Our findings of quality of nutrition service provision at ANC is consistent with the poor content of ANC service reported in Bangladesh and other low-income settings [11, 17, 20]. However, previous studies mostly explored the content of ANC based on the recall by women with a recent birth if they had received listed services at any time during pregnancy, but not at any specific service contract [11, 15, 20]. Point of care observation-based studies from Peru and Tanzania reported low adherence to recommended service provision at ANC [19, 21]. An earlier study in Bangladesh also reported a high level of weight measurement and IFA provision but inadequate nutrition counselling at ANC [13].

Adequate readiness of the health facilities is essential to ensure any service provision [11, 33-35]. Our study showed that improved logistical readiness of the health facilities is strongly associated with a higher quality of nutrition services provision at ANC. In the desegregated analysis, we found weight assessments was substantially lower in the facilities with poor logistical readiness compared with the facilities with better logistical readiness. However, the provision of IFA did not differ significantly by the logistical readiness levels of the facilities as IFA was available in most (93%) of the facilities. Although unadjusted

analysis showed an inverse association between infrastructural readiness of facilities and quality nutrition service provision, this association is attenuated and no longer significant in the adjusted analysis. The crude association is likely to be confounded by the higher-tier facilities with better infrastructural readiness but poorer service provision as shown in our descriptive analysis. Moreover, we found better quality service provision by lower-level providers who predominately offer services at lower-tier facilities than physicians and nurses at higher-tier facilities. Previous studies from LMICs also reported that service provision by lower-level providers offered similar or better compliance to standard maternal and child out-patient care than providers at the higher-level facility [22, 36]. The NNS programme should ensure a consistent supply of the logistics and supplies that are essential for nutrition service provision at ANC to improve the quality of service. There should be an emphasis on role clarification of the providers at the higher level facility based on WHO and national recommendations of nutrition services during pregnancy.

Our study demonstrates that HCP-client interactions like informing them about the progress of pregnancy and giving them the opportunity to ask questions had a significant association with quality nutrition service provision. Both WHO's standards for maternal and newborn care and WHO guidelines on ANC recommend treating clients with dignity and compassion, and providing information on health status and available interventions for making an informed choice by clients [9, 37]. Earlier studies also reported the importance of HCP's interpersonal communication and attitude towards clients to improve quality ANC service provision at health facilities [16, 18]. A study in Lao reported, based on both HCP and client's opinion, that HCP's behaviour, mutual communication and treating clients equally with dignity are important considerations to improve ANC service quality [16]. All this evidence highlights the importance of enhancing client provider interactions for improving the quality of ANC nutrition services. These enhancements should include providing client's adequate information and treating them with dignity so that they feel empowered and confident to ask questions.

We also found that job aid tools like visual aids and the ANC card during ANC consultation improved the quality of nutrition service, especially on weight measurement, anaemia assessment, and nutrition counselling. A possible reason could be the requirement that the HCPs document the key components of nutrition services provided on the ANC card, which acts as a reminder to ensure the services [38]. Similar to the ANC card, visual aids are likely to act as a prompt to remind HCPs of the counselling contents, improved communication technique and service provision[39]. There was no association between the use of ANC card or visual aid and IFA supplementation. The ANC card used in public facilities in Bangladesh does not include any item on IFA provision. Moreover, IFA provision is likely to depend more on the availability of the supplements, the HCP's perceptions about supplementation, and client characteristics.

In contrast to previous studies, we did not find any association of HCP's nutrition training with the quality of nutrition service delivery during ANC [21, 40]. The content and quality of their training in nutrition might explain this finding. There are several criticisms of the Basic Nutrition Training received by that most of the HCPs. There was too much content to cover in a short training time. It focused too much on child feeding, and there were, few practical demonstrations. and the training lost quality when implemented to a large number of HCPs throughout the country using a cascade model [41, 42]. Receiving external supervision was positively associated only with IFA provision only. Like training on nutrition, the quality of supervision visits to HCPs is often a question in resource-poor settings. Administrative supervisors often do the external supervision received by HCPs without having much technical knowledge and supervisory skills, which results in a missed opportunity to reinforce the implementation of the knowledge and training obtained by HCPs into practice [41, 43]. Considering the quality issue of nutrition training, in 2017, the current NNS operation plan introduced Comprehensive Competency-Based Training on Nutrition, which is implemented by technical experts [10]. The Competency-Based Training also addressed the supervision issue by developing and training supervisors on specifically designed supervisor modules to improve the

technical competence of the supervisors. However, we do not know if the Competency-Based Training in overcoming these challenges.

Gestation was an important determinant of receiving higher quality ANC. Women coming for ANC in late trimesters were more likely to receive better quality nutrition services compared to women presenting in the first trimester [13, 22, 44]. The women presenting in the first trimester were less likely to have their weight assessed or receive IFA supplements. Low IFA supplement provision in the first trimester could be related to some inconsistent perceptions among HCPs on the timing of starting IFA and the adverse effect of iron on foetal development [45, 46]. HCP's perception of the importance of different services may also differ according to the gestational age. A study in Ghana suggested that HCP sometimes consider physical measurements not important in the first trimester [22]. However, the reasons for lower compliance with weight assessment in the earlier stage of pregnancy require further in-depth research. Detection and management of anaemia and provision of IFA early in pregnancy are important to avert its detrimental effect on maternal and child health outcomes[47]. Adaptation of the national ANC guideline to comply with the global recommendations and to improve perceptions of the HCPs on the recommended nutrition service at ANC may enhance the quality of service provision.

The main strength of our study is the collection of observation-based data on nutrition services provision at ANC and the assessment of the facility, provider, and client characteristics at the same time-point which allowed us to conduct the supply side and client-level determinant analysis. The sampling of facilities covered 21 out of 64 districts spread across all geographic regions, which improved the generalizability of the study findings to public facilities in Bangladesh. Our study has some limitations. Firstly, observation of service provision by an external assessor may have resulted in some improved practice by HCPs. However, we anticipate a limited effect of such bias on the determinant analysis as facility and provider characteristics would not change during the observation. Besides, we conducted the

observations in a natural setting, spending a reasonable time, i.e., 1-2 full days, at each facility and assuring the HCPs about their anonymity when presenting findings. Also, we stressed that our observations were not related to individual performance assessment to try and reduce the Hawthorne effect [48]. Secondly, the study methodology did not involve any in-depth interview with HCPs to explore the reasons for low compliance to weight assessment in the women presenting in the first trimester of pregnancy and the limited impact of training and supervision on quality service provision. Thirdly, we could not also account for hospital management and governance level determinants of providing quality nutrition service at health care platforms like ANC. Future research should explore these questions.

Conclusion

ANC is the main platform to deliver nutrition services during pregnancy. The quality of nutrition service provided at ANC in public health facilities in Bangladesh is sub-standard. On the contrary to WHO and national recommendations, we observed differential practice in nutrition service provision by gestational age of the clients, which necessitates improving awareness of ANC standards and guidelines among HCPs. Logistical readiness of the facility influences the quality of nutrition service provision and requires urgent attention by the nutrition program managers. Better HCP-client communications like providing information about the progress of pregnancy and allowing them to ask questions were associated with improved the quality of the service. Future HCP's capacity building and quality-focused supportive supervision should emphasize these key elements of service provision . The utilization of simple but useful tools like ANC cards and visual aids needed across all ANC services to improve the quality of nutrition service. Redesigning the content and implementation modality of HCP's training and assessing the long-term effect of such training in improving HCP's practice is imperative. Lastly, the traditional hierarchical administrative-focused supervision failed to improve the quality of nutrition service provision, which calls

for a paradigm shift to service-specific supportive supervision by technical experts to harness the benefit of supervision.

Acknowledgements

We thank the Directorate General Health Services, Directorate General Family Planning and National Nutrition Service of Ministry of Health and Family Welfare for approving us to conduct the study at public health facilities. We acknowledge the contribution of physicians and research assistants involved in the assessment. We are grateful to all participants for allowing us to conduct the assessment and interviews. icddr,b is also grateful to the Governments of Bangladesh, Canada, Sweden and the UK for providing core/unrestricted support.

References

1. Victora CG, Christian P, Vidaletti LP, Gatica-Domínguez G, Menon P, Black RE. Revisiting maternal and child undernutrition in low-income and middle-income countries: variable progress towards an unfinished agenda. The Lancet. 2021.
2. Christian P, Mullany LC, Hurley KM, Katz J, Black RE. Nutrition and maternal, neonatal, and child health. Seminars in perinatology. 2015;39(5):361-72.
3. National Institute of Population Research and Training (NIPORT), Mitra and Associates, and ICF International. Bangladesh Demographic and Health Survey 2014. Dhaka, Bangladesh, and Rockville, Maryland, USA: NIPORT, Mitra and Associates, and ICF International, 2016.
4. National Institute of Population Research and Training (NIPORT), Mitra and Associates, ICF. Bangladesh Demographic and Health Survey 2017-18: Key Indicators. Dhaka, Bangladesh, and Rockville, Maryland, USA: National Institute of Population Research and Training (NIPORT), ICF, 2019.
5. Arsenault JE, Yakes EA, Islam MM, Hossain MB, Ahmed T, Hotz C, et al. Very low adequacy of micronutrient intakes by young children and women in rural Bangladesh is primarily explained by low food intake and limited diversity. The Journal of nutrition. 2013;143(2):197-203.
6. Abera SF, Kantelhardt EJ, Bezabih AM, Tsadik M, Lauvai J, Ejeta G, et al. What factors are associated with maternal undernutrition in eastern zone of Tigray, Ethiopia? Evidence for nutritional well-being of lactating mothers. BMC public health. 2020;20(1):1-12.
7. Kuhnt J, Vollmer S. Antenatal care services and its implications for vital and health outcomes of children: evidence from 193 surveys in 69 low-income and middle-income countries. BMJ open. 2017;7(11):e017122.
8. Haileslassie K, Mulugeta A, Girma M. Feeding practices, nutritional status and associated factors of lactating women in Samre Woreda, South Eastern Zone of Tigray, Ethiopia. Nutrition journal. 2013;12(1):1-11.
9. World Health Organization. WHO recommendations on antenatal care for a positive pregnancy experience: WHO; 2016.
10. GoB. 4th Health, Population and Nutrition Sector Programme (4th HPNSP), Operational Plan, National Nutrition Service (NNS) Institute of Public Health Nutrition, Directorate General of Health Service, Ministry of Health and Family Welfare, Government of the People's Republic of Bangladesh, 2017.
11. Islam MM, Masud MS. Determinants of frequency and contents of antenatal care visits in Bangladesh: Assessing the extent of compliance with the WHO recommendations. PLoS One. 2018;13(9):e0204752.
12. Nguyen PH, Khương LQ, Pramanik P, Billah SM, Menon P, Piwoz E, et al. Effective coverage of nutrition interventions across the continuum of care in Bangladesh: insights from nationwide cross-sectional household and health facility surveys. BMJ open. 2021;11(1):e040109.
13. Billah SM, Saha KK, Khan ANS, Chowdhury AH, Garnett SP, Arifeen SE, et al. Quality of nutrition services in primary health care facilities: Implications for integrating nutrition into the health system in Bangladesh. PLoS One. 2017;12(5):e0178121-e. doi: 10.1371/journal.pone.0178121. PubMed PMID: 28542530.
14. Donabedian A. The quality of care: How can it be assessed? JAMA. 1988;260(12):1743-8.
15. Afulani PA, Buback L, Essandoh F, Kinyua J, Kirumbi L, Cohen CR. Quality of antenatal care and associated factors in a rural county in Kenya: an assessment of service provision and experience dimensions. BMC health services research. 2019;19(1):1-16.

16. Phommachanh S, Essink DR, Jansen M, Broerse JE, Wright P, Mayxay M. Improvement of Quality of Antenatal Care (ANC) Service Provision at the Public Health Facilities in Lao PDR: Perspective and Experiences of Supply and Demand Sides. BMC pregnancy and childbirth. 2019;19(1):1-13.
17. Kanyangarara M, Munos MK, Walker N. Quality of antenatal care service provision in health facilities across sub–Saharan Africa: Evidence from nationally representative health facility assessments. Journal of global health. 2017;7(2).
18. Emiru AA, Alene G, Debelew GT. Women's satisfaction with the quality of antenatal care services rendered at public health facilities in Northwest Ethiopia: the application of partial proportional odds model. BMJ open. 2020;10(9):e037085.
19. Wynne SJ, Duarte R, de Wildt G, Meza G, Merriel A. The timing and quality of antenatal care received by women attending a primary care centre in Iquitos, Peru: A facility exit survey. PLoS One. 2020;15(3):e0229852.
20. Singh L, Dubey R, Singh S, Goel R, Nair S, Singh P. Measuring quality of antenatal care: a secondary analysis of national survey data from India. BJOG: An International Journal of Obstetrics & Gynaecology. 2019;126:7-13.
21. Bintabara D, Nakamura K, Ntwenya J, Seino K, Mpondo BC. Adherence to standards of first-visit antenatal care among providers: A stratified analysis of Tanzanian facility-based survey for improving quality of antenatal care. PLoS One. 2019;14(5):e0216520.
22. Amoakoh-Coleman M, Agyepong IA, Zuithoff NP, Kayode GA, Grobbee DE, Klipstein-Grobusch K, et al. Client factors affect provider adherence to clinical guidelines during first antenatal care. PLoS One. 2016;11(6):e0157542.
23. Goudet S, Murira Z, Torlesse H, Hatchard J, Busch-Hallen J. Effectiveness of programme approaches to improve the coverage of maternal nutrition interventions in South Asia. Maternal & child nutrition. 2018;14 Suppl 4(Suppl 4):e12699-e. doi: 10.1111/mcn.12699. PubMed PMID: 30499258.
24. Billah SM, Arifeen SE. Evaluation of GoB, UNICEF, CIFF initiative to facilitate and accelerate implementation of the Bangladesh National Nutrition Services Registry for International Development for Impact Evaluations (RIDIE), Available at: 10.23846/ridie0902016. Available from: https://ridie.3ieimpact.org/index.php?r=search/detailView&id=415.
25. Billah SM, Khan ANS, Raihana S, Rahman M, Salam SS, Arifeen SE, et al. Evaluation of Accelerating Implementation of the Bangladesh National Nutrition Services: Initial Assessment 2016. 2016.
26. Campbell SM, Roland MO, Buetow SA. Defining quality of care. Social science & medicine. 2000;51(11):1611-25.
27. Bruce J. Fundamental elements of the quality of care: a simple framework. Studies in family planning. 1990;21(2):61-91.
28. NIPORT, ACPR, ICF International. Bangladesh Health Facility Survey 2014. Dhaka, Bangladesh: National Institute of Population Research and Training (NIPORT), Associates for Community and Population Research (ACPR) and ICF International, 2016.
29. MOH&FW. Maternal Health Standard Operating Procedure (SOP) Volume-1. Bangladesh: Ministry of Health and Family Welfare, 2015.
30. NIPORT, Mitra and Associates, and ICF International. Bangladesh Demographic and Health Survey 2014. . Dhaka, Bangladesh, and Rockville, Maryland, USA:: National Institute of Population Research and Training (NIPORT), Mitra and Associates, and ICF International., 2016.
31. Owili PO, Muga MA, Mendez BR, Chen B. Quality of maternity care and its determinants along the continuum in Kenya: A structural equation modeling analysis. PLoS One. 2017;12(5):e0177756.
32. Harris T, Yang Z, Hardin JW. Modeling underdispersed count data with generalized Poisson regression. The Stata Journal. 2012;12(4):736-47.

33. Mansur AM, Rezaul KM, Mahmudul HM. Quality of antenatal care in primary health care centers of bangladesh. Journal of family & reproductive health. 2014;8(4):175.
34. Rahman A, Nisha MK, Begum T, Ahmed S, Alam N, Anwar I. Trends, determinants and inequities of 4+ ANC utilisation in Bangladesh. Journal of Health, Population and Nutrition. 2017;36(1):2.
35. Stelfox HT, Straus SE. Measuring quality of care: considering measurement frameworks and needs assessment to guide quality indicator development. Journal of clinical epidemiology. 2013;66(12):1320-7.
36. Hoque DE, Arifeen SE, Rahman M, Chowdhury EK, Haque TM, Begum K, et al. Improving and sustaining quality of child health care through IMCI training and supervision: experience from rural Bangladesh. Health Policy Plan. 2014;29(6):753-62.
37. World Health Organization. Standards for improving quality of maternal and newborn care in health facilities. Switzerland: WHO, 2016 9241511214.
38. Gross K, Schellenberg JA, Kessy F, Pfeiffer C, Obrist B. Antenatal care in practice: an exploratory study in antenatal care clinics in the Kilombero Valley, south-eastern Tanzania. BMC pregnancy and childbirth. 2011;11(1):1-11.
39. Jennings L, Yebadokpo AS, Affo J, Agbogbe M. Antenatal counseling in maternal and newborn care: use of job aids to improve health worker performance and maternal understanding in Benin. BMC pregnancy and childbirth. 2010;10(1):1-13.
40. Omer AM, Haile D, Shikur B, Macarayan ER, Hagos S. Effectiveness of a nutrition education and counselling training package on antenatal care: a cluster randomized controlled trial in Addis Ababa. Health Policy Plan. 2020;35(Supplement_1):i65-i75. doi: 10.1093/heapol/czaa101. PubMed PMID: 33165586.
41. Saha KK, Billah M, Menon P, El Arifeen S, Mbuya NV. Bangladesh National Nutrition Services: Assessment of Implementation Status: World Bank Publications; 2015.
42. Ajeani J, Mangwi Ayiasi R, Tetui M, Ekirapa-Kiracho E, Namazzi G, Muhumuza Kananura R, et al. A cascade model of mentorship for frontline health workers in rural health facilities in Eastern Uganda: processes, achievements and lessons. Global health action. 2017;10(sup4):1345497.
43. Karuga RN, Mireku M, Muturi N, McCollum R, Vallieres F, Kumar M, et al. Supportive supervision of close-to-community providers of health care: Findings from action research conducted in two counties in Kenya. PLoS One. 2019;14(5):e0216444.
44. Berehe TT, Modibia LM. Assessment of Quality of Antenatal Care Services and Its Determinant Factors in Public Health Facilities of Hossana Town, Hadiya Zone, Southern Ethiopia: A Longitudinal Study. Advances in Public Health. 2020;2020:NA-NA.
45. Alam A, Rasheed S, Khan NU, Sharmin T, Huda TM, Arifeen SE, et al. How can formative research inform the design of an iron-folic acid supplementation intervention starting in first trimester of pregnancy in Bangladesh? 2015;15(1):374.
46. Siekmans K, Roche M, Kung'u JK, Desrochers RE, De-Regil LMJM, nutrition c. Barriers and enablers for iron folic acid (IFA) supplementation in pregnant women. 2018;14:e12532.
47. Di Renzo GC, Spano F, Giardina I, Brillo E, Clerici G, Roura LC. Iron deficiency anemia in pregnancy. Women's Health. 2015;11(6):891-900.
48. Oswald D, Sherratt F, Smith S. Handling the Hawthorne effect: The challenges surrounding a participant observer. Review of social studies. 2014;1(1):53-73.

Supporting information

S1 Table. Definition of outcome and explanatory variables and data collection methods

Figure 1

Click here to access/download;Figure;Fig 1.tif

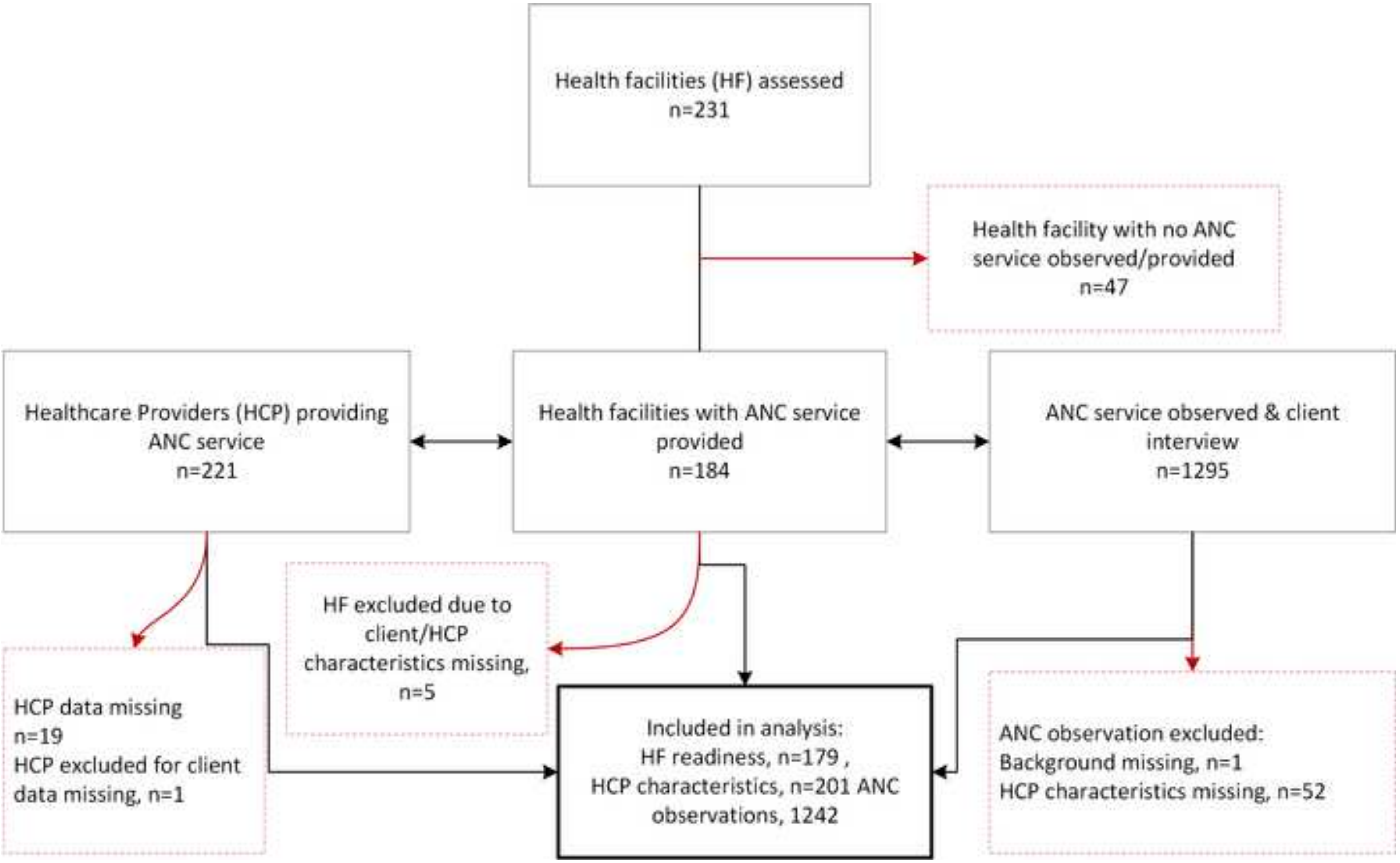

Figure 2

Click here to access/download;Figure;Fig 2.tif

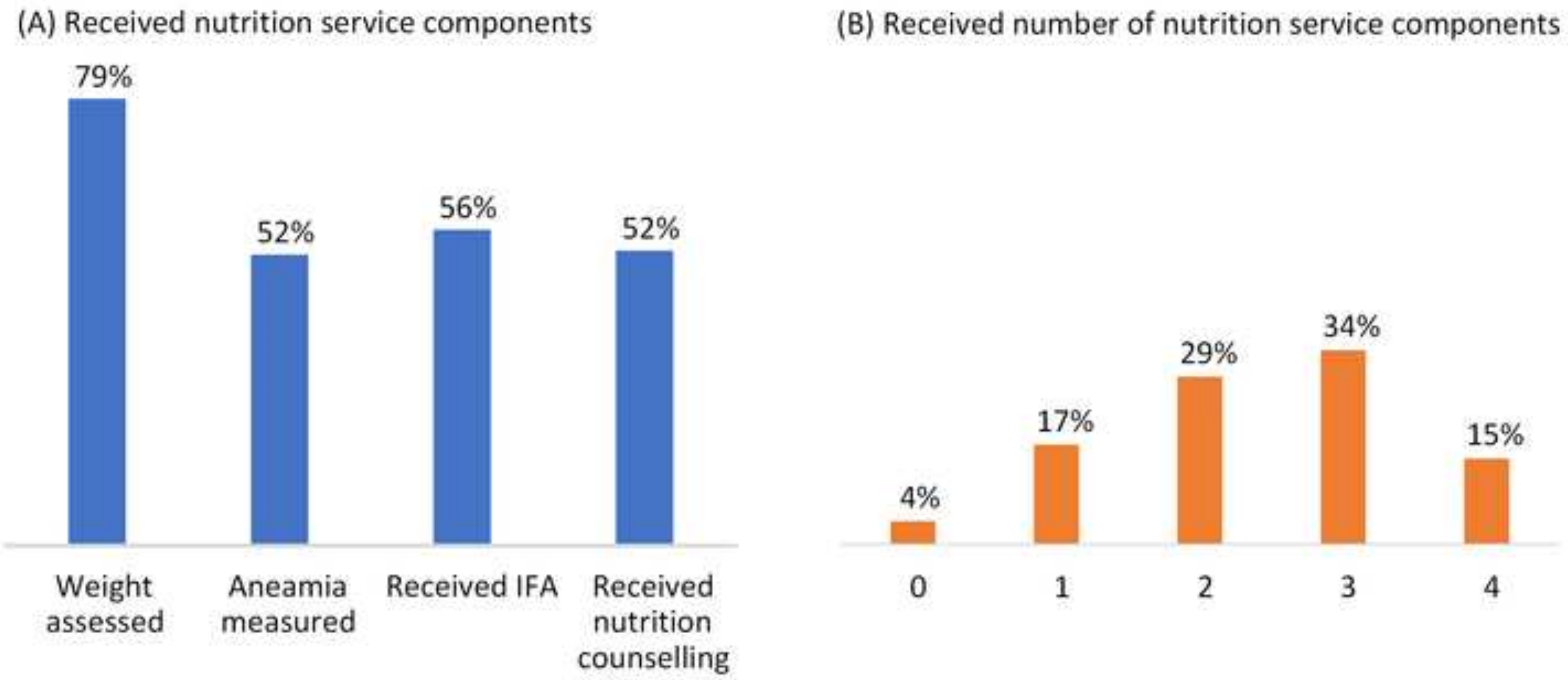

S1 Table

Click here to access/download
Supporting Information
S1_Table.docx
