# Supplementary material for: Factors influencing quality nutrition service provision at antenatal care contacts: Findings from a public health facility-based observational study in 21 districts of Bangladesh
Source: PLoS One. 2022 Jan 27;17(1):e0262867. doi: 10.1371/journal.pone.0262867 (PMC8794200; doi:10.1371/journal.pone.0262867)
Supplement: Attachment — Submitted filename: PONE-D-21-16534_R1 Dated 10 Dec 21.pdf [file pone.0262867.s004.pdf]

PLOS ONE

Factors influencing quality nutrition service provision at antenatal care contacts: findings from a public health facility-based observational study in 21 districts of Bangladesh

--Manuscript Draft--

Manuscript Number:	PONE-D-21-16534R1
Article Type:	Research Article
Full Title:	Factors influencing quality nutrition service provision at antenatal care contacts: findings from a public health facility-based observational study in 21 districts of Bangladesh
Short Title:	Factors influencing quality nutrition service provision at antenatal care contacts
Corresponding Author:	Sk Masum Billah<br>International Centre for Diarrhoeal Disease Research, Bangladesh<br>Dhaka, BANGLADESH
Keywords:	Quality of Health Care, Maternal Nutrition, Antenatal Care, Job Aids
Abstract:	Malnutrition during pregnancy is associated with increased maternal morbidity and mortality and has a long-term negative impact on child growth and development. Antenatal care (ANC) is the formal point of contact for pregnant women to receive preventive health and nutrition services. We assessed the quality of nutrition service delivery during ANC and examined its determinants related to the health facility, health care provider (HCP) and client characteristics. We conducted a cross-sectional assessment in 179 facilities, including 1242 ANC observations and exit interviews of the pregnant women from 21 districts in Bangladesh. We defined 'quality nutrition service' outcome by adding the counts of four services, including maternal weight measurement, anaemia assessment, nutrition counselling and iron-folic acid (IFA) supplement provision. We explored both the supply side and the client-level determinants of quality nutrition service using multilevel Poisson regression. Overall, only 15% of clients received all four nutrition services. Performance of weight measurement (79%) was higher than IFA provision (56%), anaemia assessment (52%) and nutrition counselling (52%). The multivariable analysis showed that quality nutrition service delivery is positively associated with good logistical readiness of the facilities (aIRR: 1.23, 95% CI: 1.08-1.39), consultation by paramedics (aIRR 1.23, 95% CI: 1.06-1.42) and community health care providers (aIRR 1.32, 95% CI: 1.12-1.57), HCP's knowledge on maternal nutrition (aIRR 1.04; 95% CI: 1.01-1.08), better HCP-client communication (aIRR 1.14; 95% CI: 1.04-1.26) and use visual aids or ANC card (aIRR 1.18; 95% CI: 1.11-1.27). We found limited associations of HCP's training and external supervision with the quality of nutrition services. The quality of nutrition service provision during ANC is suboptimal. Public health nutrition programmers should ensure the facilities' logistical readiness, revisits and reinforce the content and modality of training and supportive supervision of the HCPs. They should also emphasize positive HCP-client communication and use of job aids to improve the quality of nutrition service provision during ANC.
Order of Authors:	Sk Masum Billah
	Nazia Binte Ali
	Abdullah Nurus Salam Khan
	Camille Raynes-Greenow
	Patrick John Kelly
	Md. Shahjahan Siraj
	Sufia Askari
	Purnima Menon
	Shams El Arifeen

Powered by Editorial Manager® and ProduXion Manager® from Aries Systems Corporation

	Michael John Dibley
	Phuong Hong Nguyen
**Response to Reviewers:**	Response to reviewers' comments<br><br>Reviewer #1:<br>1.The study has addressed an important public health issue for Bangladesh, and other LIMCs. However, one thing should be noted, the four factors which were finally revealed as independent determinants (logistical readiness of the facilities, HCP's knowledge on maternal nutrition, better HCP-client communication, and use visual aids or ANC card) are essential pre-requisite for delivering quality nutrition service. If the pre-requisites are not fulfilled, it is not possible to deliver quality service. So, in discussion and conclusion sections of the papers should focused on the other factor (service provider: Doctors/paramedics/CHCP) as it is not a prerequisite like other four factors. Considering these issues, the authors should revised the term 'Determinants' used in the Title and introduction section.<br><br>Response: Our study hypothesizes that facility readiness, health care providers, the process of care, and client characteristics all contribute to the provision of high-quality nutrition services at antenatal care contact. We examined a variety of variables in each category (including provider types) and identified which of those variables are associated with (or may influence) high-quality service. We have revised our title and introduction, replacing the term "Determinants" by "Factors influencing". In the discussion, we have included practice variations among different types of providers and essential programmatic inputs to mitigate the practice gap (page 19, lines 354-362) and now have included them in the conclusion (page 23 lines 444-445).<br><br>Reviewer #2:<br>1.ANC is the classic best entry point for counseling expecting mothers on proper nutrition, danger signs of pregnancy and monitor the health of the mother and the fetus. The authors looked at a critical health system fitness to provide quality nutrition services for pregnant women. The research questions are relevant and the methodology is sound. The results are clearly described and the implications well discussed.<br><br>Response: Thank you for your comments on our manuscript.<br><br>Just one curious question. What did the authors think was the reason for the finding that the quality of nutrition services given during the 2nd and 3rd trimester was better than the one during the 1st trimester? I know that some misperception or misunderstanding from the provider side was mentioned. A bit more explanation would be great, if possible.<br><br>Response: We have discussed some possible explanations in the discussion (page 21, lines 404-412). The difference in the quality of nutrition service across different trimesters of pregnancy is mainly due to the providers' perception gap and self-prioritisation of some services over others. For instance, among the four service components included in the quality of nutrition service during ANC, weight measurement and provision of IFA supplements were poorer in the 1st trimester compared to women in the 2nd and 3rd trimesters. Sometimes health care providers choose not to offer IFA in early pregnancy fearing iron's adverse effect on foetal development. Similarly, sometimes they disregard physical measurement and weight assessment during early pregnancy. It is unlikely that the client's demand or choice for service played a big role in this case, as clients often lack knowledge/perception of good quality care and what to anticipate from service providers when seeking health care in low and middle-income settings.<br><br>Reviewer #3:<br>This manuscript will be a useful addition to our knowledge of nutrition services provided during pregnancy, especially for ANC in Low and Low Middle Income Countries.<br><br>Response: Thank you for your encouraging comments on our manuscript.

Powered by Editorial Manager® and ProduXion Manager® from Aries Systems Corporation

1.I have a few clarification/suggestions that should be addressed;
Lines# 23 to 25. Definition is not clear. What is being counted? Will weight measurements done twice and anemia measurements done two times in one woman across pregnancy be counted as equivalent to weight measurement done once and and anemia measure done three times in another women?

Response: We counted the number of the selected four services provided at each ANC consultation as the ‘quality nutrition service’ indicator from observation of the ANC consultations. It was a cross-sectional study, and we observed ANC service consultation for a woman only once. To avoid confusion, we have revised the text as:
“We considered four essential nutrition services at each ANC contact including maternal weight measurement, anaemia assessment, nutrition counselling and iron-folic acid (IFA) supplement provision. We defined a composite ‘quality nutrition service’ outcome by counting the number of services (out of four) provided at each ANC from observation data” (page 2, lines 23-26).

2.Lines# 64 and 65. Some information is missing. Rephrase the statement for clarity.

Response: We have rephrased the sentence to improve clarity (page 4, lines 65-68).
“Another challenge is ensuring the quality of nutrition interventions provided at ANC contacts, without which programmes will not fully harness the benefits of these interventions on maternal and child nutrition outcomes. Moreover, a recent study reported incomplete readiness of healthcare facilities to provide nutrition interventions during ANC (51%).”

3.Line#175. typo.

Response: We have corrected the typo (page 10, line 203)

4.Table 1: Haemoglobin testing tool. Was it expected that sub-districts and community clinics will be better than district hospitals? Are they special programs/interventions that are being run by the health authority in Bangladesh that focus on resourcing rural and sub-district hospitals as compared to district hospitals? In most LMICS we see that district level hospitals are better equipped that rural clinics.

Response: In general, districts and sub-district level facilities have better readiness than union-level health facilities and community clinics. However, the availability of logistics and tests to provide ANC service is expected to be similar across all tiers of facilities. One explanation of poorer on-site readiness of haemoglobin testing at the district and sub-district hospital is out of the Tallquist strips due to high client load and availability of other advanced diagnostic facilities at district and sub-district levels. Health care providers sometimes refer the clients to these diagnostic labs for haemoglobin testing rather than doing the rapid test onsite. We did not consider the availability of advanced diagnostics tests for anaemia outside of ANC consultation as HCPs can recommend the test but do not take a decision if the test is done afterward. However, we have included the following text in the limitation (pages 22-23, lines 437-439)
“Fourth, we assessed only the on-site availability of rapid haemoglobin testing kits at the ANC room which might under-estimate the haemoglobin testing capacities at district and sub-district hospitals as some of them may have advanced diagnostic tests for anaemia.”

5.Table 1: What does "ANC utilization per day" mean?

Response: ANC utilization per day means the average number of ANC services provided on a day at the facility. We have now clarified this in Table 1 (page 11)

6.What was the effect of clients (pregnant women) educational status on the outcome variables?

Response: We did not find any association between the client’s educational status and the quality of nutrition service provided at ANC. We have reported this in table 3. Education was not associated with any of the four selected nutrition services in the

Powered by Editorial Manager® and ProduXion Manager® from Aries Systems Corporation

bivariate analyses and we did not include it in the multiple regression model presented in Table 4.

7.Figure 2 B: should the items add up to 100%? Currently it adds up 99%.

Response: We have corrected the rounding error in Figure 2B.

Reviewer #4:
1.Methods
Upon what basis districts were selected?

Response: We have expanded the text on district selection as follows (page 5-6, lines 103-111):
“We selected the districts based on comparability of population density, literacy rate, housing characteristics, people in the lowest wealth quintile, access to a safe drinking water source, electricity connection, improved sanitation, coverage of childhood immunization, ANC, skilled birth attendance, postnatal care and modern methods of family planning, under-five mortality rate and childhood stunting prevalence. We created a score for each district by principal component analysis of these variables. Then, we applied nearest neighbour matching of district’s PCA score to match two intervention districts with one non-intervention district. Finally, we selected seven matched groups of districts (each having two AINNS intervention districts and one non-intervention), which had the minimum difference in the PCA score.”

2.Line 109:Better to mention Upa Zilla as sub district is non existent

Response: We have added upazila health complexes and mentioned sub-district hospitals in parenthesis in line 123 on page 6. However, we have used sub-district hospital throughout the paper for the ease of understanding of the global audience.

3.Please mention how anaemia was defined

Response: We have defined anaemia screening as (page 8, lines 169-171):
“Screening for anaemia included assessing blood haemoglobin level either using Tallquist paper onsite, by previous laboratory investigation or examining eye or palm (clinical assessment)”.

4.Conclusion:
Need to make brief depending upon objective

Response: We have revised the conclusion and made it relevant only to the study objectives and findings (page 23, lines 445-454).

Reviewer #5:
General comments
1.This manuscript is very interesting having very important findings, but the paper required same professional proofreading to edit the entire paper for language, grammatical, spelling, and punctuations to improve the paper for publication. Once the authors are able to address the concerns appropriately, the manuscript can be published.

Response: We have proof-read the manuscript and corrected typos and grammatical errors.

2.The four main target variables (maternal weight measurement, anaemia assessment, nutrition counselling and iron-folic acid (IFA) supplementation) for this study was not properly addressed and exhausted, need to elaborate more.

Response: We have elaborated the definitions of four target variables for quality of nutrition service in lines 164-174 on page 8:
“We considered weight assessment performed if the HCPs took the weight of pregnant women using either a digital or analogue weighing scale”. “Screening for anaemia included assessing blood haemoglobin level either using Tallquist paper onsite, by

Powered by Editorial Manager® and ProduXion Manager® from Aries Systems Corporation

	previous laboratory investigation or examining eye or palm (clinical assessment). Provision of nutrition counselling included HCPs providing messages on dietary diversity, quantity and types of nutritious food. We defined IFA provision as HCPs distributing IFA supplements during the ANC consultation; however, we did not include HCPs prescribing IFA to be bought from outside pharmacies".<br><br>3.Methods<br>Page 7, line 133 – 137 the paragraph "We conducted the assessment in 231 facilities, and in 184 facilities, we observed ANC services provided on the assessment days. We interviewed 217 health care providers who offered ANC services at the facilities on the day of the visit, and we observed 1296 ANC consultations. We excluded 54 observations due to missing information about the health care providers, the client's characteristics, or the exit interview" is not clear, need to be reconsidered or rephrased.<br><br>Response: We have rephrased the text as (page 7-8, lines 149-157):<br>"We assessed the readiness to provide ANC services in 231 facilities (Fig 1). ANC services were not sought in 47 out of the 231 facilities on the assessment days; thus, we observed 1295 ANC service consultations at 184 facilities. We had complete background information from 201 HCPs who offered ANC services on the day of the visit. We excluded 53 observations from the analysis due to missing information on HCPs or the client characteristics. Finally, we included 1,242 observations of ANC service consultation provided by 201 service providers at 179 facilities in the analysis for this paper".<br><br>4.Result<br>Page 10 line 213 & 2014 the sentence "On average, clients who received ANC from the facilities were 23 years old and had eight years of schooling" is not clear, need more explanation?.<br><br>Response: We have rephrased the text as (page 12, lines 241-242):<br>"Clients who received ANC from the facilities had a mean age of 23 years (SD±4.3 years) and had a mean eight years (SD±2.9 years) of schooling".<br><br>5.Page 14, table 4 are highly congested and it crosses one page requires rearrangement or division into two table or more.<br><br>Response: We have divided Table 4 into two tables (Table 4 and Table 5).<br><br>6.Discussion<br>Grammatical, spelling, and punctuations improvement is required in this section<br><br>Response: We have corrected grammatical, spelling and punctuation errors.<br><br>7.Overall comment<br>There are some areas need corrections; such as rephrasing, grammatical, spelling, and language.<br>Kindly review issues raised and if possible get a trained English proof reader to edit the entire manuscript.<br><br>Response: We have revised accordingly.
**Additional Information:**	
**Question**	**Response**
**Financial Disclosure**<br><br>Enter a financial disclosure statement that describes the sources of funding for the work included in this submission. Review the submission guidelines for detailed requirements. View published research articles from *PLOS ONE* for specific	The lead author, SMB, received funding from Children's Investment Fund Foundation (CIFF) for conducting the initial assessment for the Evaluation of Accelerating Implementation of the National Nutritional Service (AINNS) project in Bangladesh (Grant number G-1612-00805). This paper uses the data from the initial assessment. Sufia Askari from the funding agency has contributed to the conception of the paper and provided inputs to the manuscript draft. The funding agency had no other role in original study design, data collection and analysis, decision to publish, or preparation of the manuscript.

Powered by Editorial Manager® and ProduXion Manager® from Aries Systems Corporation

examples.<br><br>This statement is required for submission and **will appear in the published article** if the submission is accepted. Please make sure it is accurate.<br><br>**Unfunded studies**<br>Enter: *The author(s) received no specific funding for this work.*<br><br>**Funded studies**<br>Enter a statement with the following details:<br>• Initials of the authors who received each award<br>• Grant numbers awarded to each author<br>• The full name of each funder<br>• URL of each funder website<br>• Did the sponsors or funders play any role in the study design, data collection and analysis, decision to publish, or preparation of the manuscript?<br>• **NO** - Include this sentence at the end of your statement: *The funders had no role in study design, data collection and analysis, decision to publish, or preparation of the manuscript.*<br>• **YES** - Specify the role(s) played.<br><br>* typeset	
**Competing Interests**<br><br>Use the instructions below to enter a competing interest statement for this submission. On behalf of all authors, disclose any competing interests that could be perceived to bias this work—acknowledging all financial support and any other relevant financial or non-financial competing interests.<br><br>This statement is required for submission and **will appear in the published article** if the submission is accepted. Please make sure it is accurate and that any funding sources listed in your Funding Information later in the submission form are also declared in your Financial Disclosure statement.	I have read the journal's policy and the authors of this manuscript have the following competing interests: Sufia Askari is from the funding agency. She contributed to the study concept but played no role in study design, data collection or data analysis. Her contributions to this manuscript included inputs to the section on interpretation of results and review of manuscript drafts. However, the final decision about the results to include, interpretation and conclusion rested with the lead author and the authors from the evaluation team. All other authors declare that no competing interests exist.

View published research articles from *PLOS ONE* for specific examples.<br><br>**NO authors have competing interests**<br><br>Enter: *The authors have declared that no competing interests exist.*<br><br>**Authors with competing interests**<br><br>Enter competing interest details beginning with this statement:<br><br>*I have read the journal's policy and the authors of this manuscript have the following competing interests: [insert competing interests here]*<br><br>* typeset	
**Ethics Statement**<br><br>Enter an ethics statement for this submission. This statement is required if the study involved:<br><br>• Human participants<br>• Human specimens or tissue<br>• Vertebrate animals or cephalopods<br>• Vertebrate embryos or tissues<br>• Field research<br><br>Write "N/A" if the submission does not require an ethics statement.<br><br>General guidance is provided below. Consult the submission guidelines for detailed instructions. **Make sure that all information entered here is included in the Methods section of the manuscript.**	Ethical Review Committee of icddr,b approved the study (Protocol Number-15107). Written informed consent was obtained from health care providers, health facility managers and clients before data collection.

Powered by Editorial Manager® and ProduXion Manager® from Aries Systems Corporation

**Format for specific study types**<br><br>**Human Subject Research (involving human participants and/or tissue)**<br>• Give the name of the institutional review board or ethics committee that approved the study<br>• Include the approval number and/or a statement indicating approval of this research<br>• Indicate the form of consent obtained (written/oral) or the reason that consent was not obtained (e.g. the data were analyzed anonymously)<br><br>**Animal Research (involving vertebrate animals, embryos or tissues)**<br>• Provide the name of the Institutional Animal Care and Use Committee (IACUC) or other relevant ethics board that reviewed the study protocol, and indicate whether they approved this research or granted a formal waiver of ethical approval<br>• Include an approval number if one was obtained<br>• If the study involved *non-human primates*, add *additional details* about animal welfare and steps taken to ameliorate suffering<br>• If anesthesia, euthanasia, or any kind of animal sacrifice is part of the study, include briefly which substances and/or methods were applied<br><br>**Field Research**<br><br>Include the following details if this study involves the collection of plant, animal, or other materials from a natural setting:<br>• Field permit number<br>• Name of the institution or relevant body that granted permission	
**Data Availability**<br><br>Authors are required to make all data underlying the findings described fully available, without restriction, and from the time of publication. PLOS allows rare exceptions to address legal and ethical concerns. See the PLOS Data Policy and FAQ for detailed information.	Yes - all data are fully available without restriction

A Data Availability Statement describing where the data can be found is required at submission. Your answers to this question constitute the Data Availability Statement and **will be published in the article**, if accepted.<br><br>**Important:** Stating 'data available on request from the author' is not sufficient. If your data are only available upon request, select 'No' for the first question and explain your exceptional situation in the text box.<br><br>Do the authors confirm that all data underlying the findings described in their manuscript are fully available without restriction?	
**Describe where the data may be found in full sentences. If you are copying our sample text, replace any instances of XXX with the appropriate details.**<br><br>• If the data are **held or will be held in a public repository**, include URLs, accession numbers or DOIs. If this information will only be available after acceptance, indicate this by ticking the box below. For example: *All XXX files are available from the XXX database (accession number(s) XXX, XXX.*).<br>• If the data are all contained **within the manuscript and/or Supporting Information files**, enter the following: *All relevant data are within the manuscript and its Supporting Information files.*<br>• If neither of these applies but you are able to provide **details of access elsewhere**, with or without limitations, please do so. For example:<br><br>*Data cannot be shared publicly because of [XXX]. Data are available from the XXX Institutional Data Access / Ethics Committee (contact via XXX) for researchers who meet the criteria for access to confidential data.*<br><br>*The data underlying the results presented in the study are available from (include the name of the third party*	All relevant data are held on a public repository and can be accessed from https://doi.org/10.6084/m9.figshare.14608206.v1

and contact information or URL).<br>• This text is appropriate if the data are owned by a third party and authors do not have permission to share the data.<br><br>* typeset	
Additional data availability information:	

Manuscript Click here to access/download;Manuscript;Manuscript.docx

Factors influencing quality nutrition service provision at antenatal care contacts: findings from a public health facility-based observational study in 21 districts of Bangladesh

Authors

Sk Masum Billah[1,2*], Nazia Binte Ali[1], Abdullah Nurus Salam Khan[1], Camille Raynes-Greenow[2], Patrick John Kelly[2], Md. Shahjahan Siraj[1], Sufia Askari[3], Purnima Menon[4], Shams El Arifeen[1], Michael John Dibley[2], Phuong Hong Nguyen[4]

[1]Maternal and Child Health Division, International Centre for Diarrhoeal Disease Research, Bangladesh (icddr,b), Dhaka, Bangladesh
[2]The University of Sydney School of Public Health, Sydney, NSW 2006, Australia
[3]Children's Investment Fund Foundation, Clifford Street. London W1S 2FT, United Kingdom
[4]International Food Policy Research Institute (IFPRI), Washington, DC., United States of America

* Corresponding author
E-mail: billah@icddrb.org

Abstract

Malnutrition during pregnancy is associated with increased maternal morbidity and mortality and has a long-term negative impact on child growth and development. Antenatal care (ANC) is the formal point of contact for pregnant women to receive preventive health and nutrition services. We assessed the quality of nutrition service delivery during ANC and examined its influencing factors related to the health facility, health care provider (HCP) and client characteristics. We conducted a cross-sectional assessment in 179 facilities, including 1,242 ANC observations and exit interviews of pregnant women from 21 districts in Bangladesh. We considered four essential nutrition services at each ANC contact including maternal weight measurement, anaemia assessment, nutrition counselling and iron-folic acid (IFA) supplement provision. We defined a composite 'quality nutrition service' outcome by counting the number of services (out of four) provided at each ANC from observation data. We explored both the supply-side and the client-level factors of quality nutrition service using multilevel Poisson regression. Overall, only 15% of clients received all four nutrition services. Performance of weight measurement (79%) was higher than IFA provision (56%), anaemia assessment (52%) and nutrition counselling (52%). The multivariable analysis showed that quality nutrition service delivery is positively associated with good logistical readiness of the facilities (aIRR: 1.23, 95% CI: 1.08-1.39), consultation by paramedics (aIRR 1.23, 95% CI: 1.06-1.42) and community health care providers (aIRR 1.32, 95% CI: 1.12-1.57), HCPs' knowledge on maternal nutrition (aIRR 1.04; 95% CI: 1.01-1.08), better HCP-client communication (aIRR 1.14; 95% CI: 1.04-1.26) and use visual aids or ANC card (aIRR 1.18; 95% CI: 1.11-1.27). We found limited associations between HCP training and external supervision with the quality of nutrition services. In conclusion, the quality of nutrition service provision during ANC is suboptimal. Public health nutrition programmers should ensure the facilities' logistical readiness, and revisit and reinforce the content and modality of training and supportive

supervision of the HCPs. They should also emphasize positive HCP-client communication and the use of job aids to improve the quality of nutrition service provision during ANC.

Introduction

Maternal nutrition is a major global public health concern. Undernutrition and micronutrient deficiencies during pregnancy are associated with increased maternal morbidity and mortality and have a long-term negative impact on child growth and development [1, 2]. Despite gradual progress in low and middle-income countries (LMIC) during the last two decades, maternal and child nutrition improvements remain an unfinished agenda, especially with the interruptions by the COVID-19 pandemic [1]. In Bangladesh, 42% of women of reproductive age (15-49 years) and nearly half of all pregnant women are anaemic, 12% are thin and 14% have short stature [3, 4]. The principal drivers for maternal malnutrition are poor-quality diets, low intake of micronutrient supplements and lack of access to quality healthcare during pregnancy [5, 6]. A comprehensive analysis of data from 69 LMICs suggested that receiving antenatal care (ANC) from appropriate sources and with recommended frequency was associated with positive birth outcomes and childhood nutrition [7]. Evidence also suggests receiving three or more ANC visits is likely to reduce maternal malnutrition by three-fold [8]. All these findings highlight the need for improving coverage and quality of nutrition services during pregnancy.

ANC is the first contact for pregnant women to health and nutrition services. “WHO antenatal care guideline for positive pregnancy experience” recommends routine supplementation of iron-folic acid (IFA), nutrition counselling and weight monitoring during ANC to ensure greater coverage of nutrition interventions during pregnancy [9]. Aligned with global recommendations, the Government of Bangladesh prioritized maternal nutrition service delivery using the ANC platform in their fourth Health

Nutrition and Population Sector Program to continue 'mainstreaming nutrition' in the health systems [10]. Like several LMICs, timely utilisation and quality of ANC remain a major challenge to increase the reach and coverage of nutrition interventions in Bangladesh [11]. In 2017, the coverage of four ANC visits was 47%, indicating more than half of pregnant women were missing the opportunity of maternal nutrition services [4]. Another challenge is ensuring the quality of nutrition interventions provided at ANC contacts, without which programmes will not fully harness the benefits of these interventions on maternal and child nutrition outcomes. Moreover, a recent study reported incomplete readiness of healthcare facilities to provide nutrition interventions during ANC (51%) [12]. Nutrition input-adjusted coverage was suboptimal (18% for ANC) and disproportionately affected the poor and women with lower educational attainment. Suboptimal quality of care for nutrition services during ANC further deteriorates the situation. An assessment of the quality of nutrition services in primary care facilities showed that only 30% of mothers received four nutrition services during ANC [13].

The quality of health service delivery primarily depends on three levels of factors: structure (readiness of the health facilities), process (provision and experience of care) and outcomes (effective coverage and health outcomes) [14]. Previous studies from LMICs have mainly reported on the quality of ANC service by using one or some of the following indicators: frequency of visits, structural readiness to provide the service, content on maternity care, client experience and their determinants [11, 15-21]. Although most of these studies covered the structural and outcome-level indicators, very few explored process-level indicators. One of the few attempts in exploring process-level factors and adherence to recommended quality provisions during ANC was made in 2016 in Tanzania [21]. The study reported that female providers, availability of routine tests and basic medicines at the public facilities and providers who received refresher training were positively associated with better adherence to quality ANC provision at a single point of contact. However, this study lacked the client-level factor and experience of care domains. Another study conducted in Ghana explored the client-level factors for quality ANC but lacked

structural and provider-level factors [22]. A recent systematic review (2018) looking at the effectiveness of nutrition programming highlighted the need for additional analysis on barriers and enablers of nutrition service delivery using ANC and maternal, newborn and child health platforms in South Asia [23]. Considering the existing evidence and current research needs, a comprehensive analysis of quality ANC service delivery including structural, process and client-level factors, will be beneficial for effective programme planning [21]. This paper aims to assess the quality of nutrition service provision during ANC and examine its influencing factors related to facility readiness, health care providers, the process of care, and client characteristics.

Methods

Study design and setting

This study is a part of an initial cross-sectional assessment of coverage and quality of maternal and child nutrition services for a health system strengthening project, Accelerating Implementation of the National Nutritional Services (AINNS) in Bangladesh. The project included interventions to improve coordination of nutrition service delivery both at the national and sub-national levels and health care providers' (HCP) skills for maternal and child nutrition service delivery at public health care platforms. Details of the project evaluation plan have been reported elsewhere [24, 25]. Briefly, the initial assessment was done in 21 out of 64 districts in Bangladesh. We selected public health facilities from 14 of 40 AINNS intervention districts and seven of 24 non-intervention districts. We selected the districts based on comparability of population density, literacy rate, housing characteristics, people in the lowest wealth quintile, access to a safe drinking water source, electricity connection, improved sanitation, coverage of childhood immunization, ANC, skilled birth attendance, postnatal care and modern methods of family planning, under-five mortality rate and childhood stunting prevalence. We created a score for each district by principal component analysis of these variables. Then, we applied nearest neighbour matching of the district's PCA

score to match two intervention districts with one non-intervention district. Finally, we selected seven matched groups of districts (each having two AINNS intervention districts and one non-intervention), which had the minimum difference in the PCA score.

The initial assessment included secondary to community-level public health facilities, namely district hospitals, sub-district hospitals, union health and family welfare centres and community clinics at the ward-level. At the district and sub-district-level hospitals, physicians with or without specialisation in obstetrics and gynaecology, nurses and paramedics provide maternal care services, including ANC. At union-level facilities, which serves a catchment of 25-30 thousand people, paramedics are the main ANC service providers. These paramedics have either a four-year medical diploma or 18 months of pre-service training including six months on midwifery skills. At community clinics, which serve six to eight thousand people in each ward, community health care providers and frontline community health workers provide ANC services. From each district, we selected the district hospital and two upazila health complexes (sub-district hospitals) from two randomly selected subdistricts for this assessment. We also randomly selected four union-level health facilities and four community clinics from a list of functioning facilities prepared in consultation with the sub-district health manager in each chosen sub-district. We conducted the initial assessment between February and June 2016.

Data collection

We used the structure and process domains of the Donabedian framework for the factors influencing quality nutrition service provision. Globally, the Donabedian framework is widely used for measuring the quality of healthcare service delivery [14, 26]. This framework identifies structure, process and outcome level indicators for a comprehensive measurement of quality of care [26, 27]. We have also considered the client characteristics as they have been found to influence the provision of ANC services [21]. We collected data at both supply and client-levels from different sources, including i) a health facility

readiness assessment, ii) health care provider interviews, iii) direct observation of ANC practices, and iv) exit interviews with the ANC clients. For health facility assessment, we used a structured checklist adapted from the Bangladesh Health Facility Assessment tool [28] to collect information on facility infrastructure, and availability of specific logistics items (including weighing scale, haemoglobin testing tool, health education material/visual aids, ANC card, clinical guideline, registers and iron-folic acid (IFA) supplements). Interviews of the ANC service providers included information about their duration of work in the facility, training status and knowledge regarding nutrition services. For direct observation, we developed the observation checklist following the standard operating procedures for maternal health services [29] to document the nutrition services provided at ANC consultation. Finally, we conducted exit interviews with clients after receiving ANC to collect information on their background, demographic and obstetric characteristics and ANC service care-seeking. S1 Table summarizes the outcome and explanatory measures, definitions and data collection methods.

Sample size

We assessed the readiness to provide ANC services in 231 facilities (Fig 1). ANC services were not sought in 47 out of the 231 facilities on the assessment days; thus, we observed 1295 ANC service consultations at 184 facilities. We had complete background information from 201 HCPs who offered ANC services on the day of the visit. We excluded 53 observations from the analysis due to missing information on HCPs or the client characteristics. Finally, we included 1,242 observations of ANC service consultation provided by 201 service providers at 179 facilities in the analysis for this paper.

Fig 1. Study flow diagram showing health facilities, health care providers and participants recruited and final sample size.

ANC: antenatal care, HCP: health care provider, HF: health facility

Measures of quality of nutrition services (outcome measures)

We considered maternal weight measurement, screening for anaemia, nutrition counselling and provision of IFA supplements as the ANC nutrition services [10]. During the observations of ANC, the assessors recorded if these services were provided. We considered weight assessment performed if the HCPs took the weight of pregnant women using either a digital or analogue weighing scale. We included anaemia assessment in the quality of nutrition service during ANC due to the high prevalence of anaemia among women of reproductive age in Bangladesh [30]. Screening for anaemia included assessing blood haemoglobin levels either using Tallquist paper onsite, by previous laboratory investigation or examining eye or palm (clinical assessment). Provision of nutrition counselling included HCPs providing messages on dietary diversity, quantity and types of nutritious food. We defined IFA provision as HCPs distributing IFA supplements during the ANC consultation; however, we did not include HCPs prescribing IFA to be bought from outside pharmacies. We created a composite score variable for 'quality nutrition service' by adding the number of services (out of four) provided at the ANC consultation. Similar methods of defining a composite score for quality service have been used in previous studies [11, 31].

Explanatory variables

We explored the supply-side and the client-level factors of quality nutrition services provided at ANC (S1 Table). According to the 'structure' domain of the Donabedian framework [14], we considered the health facility characteristics and readiness and HCP characteristics and knowledge as supply-side factors. At the health facility-level, we constructed two indicators. The first was structural readiness (if all five readiness items were available). The logistical readiness indicator was created from seven logistic and supplies items using principal component analysis and then categorized into four logistical readiness quantiles. Cronbach's alpha for the logistics and supplies included in the composite score calculation was 0.62. HCP characteristics included age, sex, duration of service at the facility and receiving external supervision at

least once in the last six months. HCPs' knowledge of nutrition services at ANC was a composite score derived by adding the correct responses to four questions (S1 Table). The process-level factor included two variables on HCP-client interaction: 'HCP-client communication' (HCP discussing the progress of pregnancy and asking if the client has any questions) and the 'use of visual aids and ANC card' during ANC service provision. Client-level factors included client characteristics, namely age, education, gestational age and the number of ANC visits.

Statistical analysis

We used descriptive analysis to summarise the client and provider characteristics, health facility readiness to provide nutrition services at ANC, and client-provider interactions during ANC consultation. We reported summary statistics by frequency and proportion or by mean (±SD) for categorical and continuous variables, respectively. We explored the differences in provider characteristics and provider-client interactions by types of providers and facility readiness by types of facilities using Analysis of Variance (ANOVA) and chi-square test for continuous and categorical characteristics variables, respectively. We checked for the linearity in the relationship between each continuous explanatory variable and nutrition service quality score by fitting the locally estimated scatterplot smoothing (loess) curve. As the outcome variable includes counts of nutrition services, we used Poisson regressions to examine the potential influencing factors of quality nutrition service at ANC [32]. We included provider and facility as the random effects in the model as clients were nested within the providers and providers were nested within the facilities. We calculated p-values using Wald tests that had robust standard errors to adjust for model violations, such as under-dispersion. In the multivariable regression model, we included the explanatory variables associated with quality nutrition service at a p-value <0.2 in the bivariate analysis. The facility type had collinearity (correlation coefficient: 0.72) with HCP type. We compared Akaike's information criterion (AIC) and Bayesian information criterion (BIC) between the models with and without facility type

and excluded facility type from the final model. We also ran multivariable Poisson regression using each of the four elements of quality nutrition services as the outcome. We reported the adjusted incidence rate ratios (aIRR) and their 95% confidence interval as the measure of association. Associations were considered statistically significant at a p-value<0.05 in the final model. We used Stata version 14 software in all statistical analyses.

Ethical approval

We obtained ethical approval from the Ethical Review Committee of icddr,b (Protocol Number-15107), and written informed consent from HCPs, health facility managers and ANC clients before data collection.

Results

The study includes data from 21 district hospitals, 40 sub-district hospitals, 61 union-level facilities and 57 community clinics. Most of the facilities (88%) had one HCP who provided ANC services on the day of assessment and participated in the study. Overall, 58% of facilities had all five infrastructural readiness items considered in this analysis (Table 1). Infrastructural readiness of community clinics (21%) and family welfare centres (56%) was poorer than higher-tier facilities, including district hospitals (95%) and sub-district hospitals (91%). The overall availability of supplies like weighing scales (82%), ANC registers (99%), IFA supplements (93%) and visual aids for counselling (88%) was high. However, only 35% of facilities had a haemoglobin testing kit to detect maternal anaemia and about one third had an ANC guideline. We found significant differences in the availability of haemoglobin testing kits, visual job aids, ANC cards and IFA supplements across different types of facilities (all p<0.01).

Table 1. Characteristics and readiness of the healthcare facilities by types of facility

	Overall (N=179)	District hospital (N=21)	Sub-district hospital (N=40)	Union health centre (N=61)	Community clinic (N=57)	p-value
**All structural readiness[1] items available**	57.5	90.5	95.0	55.7	21.0	<0.001
**Logistics and supplies**						
Weighing scale	82.1	81.0	72.5	85.3	86.0	0.318
Haemoglobin testing tool	35.2	9.5	17.5	50.8	40.4	<0.001
Health education materials/visual aids	88.3	81.0	72.5	93.4	96.5	0.001
ANC card	84.9	71.4	72.5	95.1	87.7	0.004
ANC guideline	30.2	38.1	30.0	32.8	24.6	0.642
ANC register	99.4	95.2	100	100	100	0.056
IFA supplement	93.3	95.2	97.5	83.6	100	0.002
**Average number of ANC services provided on a day**						
<5	44.7	9.5	42.5	57.4	45.6	<0.001
5-10	35.8	23.8	30.0	36.1	43.9	
≥11	19.6	66.7	27.5	6.6	10.5	

Values are %; p-values are test of difference in facility readiness across facility types; [1] Structural readiness: availability of examination room/area, privacy, examination bed, electricity and functioning toilet for clients
ANC: antenatal care, IFA: iron- folic acid

Most of the HCPs (86%) were female (Table 2). About half of the community HCPs received nutrition training compared to only 9% of physicians. Knowledge of nutrition services was similar across the HCP types. Although 84% of HCPs received external supervision at least once in six months preceding the assessment, the external supervision of nurses was much lower (55%) compared to other types of HCP (>80%). HCP-client interaction during ANC consultation varied significantly by types of providers (Table 2). While the proportion of HCPs informing clients about the progress of their pregnancy or allowing clients to ask questions was high (73%), visual aid or ANC card usage during counselling was low (only in 47% of ANC consultations). Clients who received ANC from the facilities had a mean age of 23 years (SD±4.3 years) and had a mean eight years (SD±2.9 years) of schooling. Clients were mostly first-time pregnant women (42%), in their second trimester (46%), and came for either the second or third ANC visit (44%).

Table 2. Characteristics of health care providers and provider-client interaction by types of health care provider

	Overall (N=201)	Physician (N=35)	Nurse (N=20)	Paramedics (N=88)	CHCP/CHW (N=58)	p-value
**HCP characteristics**						
Age of provider (years)	38.1 (±10.0)	35.7 (±6.8)	41.4 (±9.1)	43.1 (±10.2)	30.9 (±6.2)	<0.001
Sex of the provider						
Male	13.9	5.7	0	4.6	37.9	<0.001
Female	86.1	94.3	100	95.5	62.1	
Duration of work at the current facility (years)						
≤3	32.3	62.9	35.0	35.2	8.6	<0.001
4-7	37.8	25.7	15.0	15.9	86.2	
≥8	29.9	11.4	50.0	48.9	5.2	
Received any in-service training on nutrition	28.9	8.6	20.0	23.9	51.7	<0.001
Knowledge (score out of 6) on nutrition services at ANC[1]	4.3 (±1.4)	4.2 (±1.6)	4.3 (±1.1)	4.3 (±1.4)	4.3 (±1.4)	0.994
Received any external supervision in last 6 months	83.6	82.9	55.0	83.0	94.8	0.001
Place of work						
District hospital	13.9	40.0	30.0	9.1	0	<0.001
Sub-district hospital	26.9	60.0	70.0	21.6	0	
Union health centre	30.4	0	0	69.3	0	
Community clinic	28.9	0	0	0	100	
**HCP-client interaction**	**N=1242**	**N=338**	**N=179**	**N=497**	**N=228**	
HCP-client communication[2]	72.6	61.8	63.1	87.7	63.2	<0.001
Used visual aids or ANC card	47.0	29.9	71.5	56.3	32.9	<0.001

Values are % or mean (± SD), p-values test difference of HCP characteristics by types of health care provider; [1]includes knowledge on the provision of Iron-folic acid, diagnosis and management of anaemia, weight measurement and diet counselling; [2]HCP discussing the progress of pregnancy, asked if the client had any question HCP: health care providers, ANC: antenatal care, CHCP: community health care providers, CHW: community health workers

Among the four nutrition services to be provided during ANC, assessment of weight for maternal weight gain during pregnancy was the highest (79%), followed by IFA provision (56%) (Fig 2A). In nearly half of the ANC consultations, HCPs screened for anaemia either by testing haemoglobin or by clinical examination of eye or palm and provided counselling on appropriate dietary practices. Overall, only 15% of clients received all four nutrition services components and about one third received three services (Fig 2B).

Fig 2. Percentage of clients received (A) nutrition service components and (B) number of nutrition service components at ANC.

IFA: Iron-folic acid

We summarized the factors influencing the overall quality of ANC nutrition services in Table 3. The unadjusted results showed that logistical readiness of the facilities, types of HCP, knowledge of the HCP on maternal nutrition, provision for external supervision, HCP-client communication, use of visual aids or ANC cards during the consultation, and gestational age at ANC visit were positively associated with quality nutrition service at ANC. In the adjusted analysis, there was an association between facilities with average and good logistical readiness with higher quality nutrition service provision (aIRR: 1.17, 95% CI: 1.04-1.32 and aIRR: 1.23, 95% CI: 1.08-1.39, respectively) compared to facilities with low readiness. Although nurses provided services of similar quality to the physicians, paramedics were 23% (aIRR: 1.23, 95% CI: 1.06-1.42) and community health care providers were 32% (aIRR: 1.32, 95% CI: 1.12-1.57) more likely to provide quality nutrition services. Each point increase in HCPs' knowledge of nutrition was associated with a 4% higher quality of nutrition services at ANC (aIRR: 1.04; 95% CI: 1.01-1.08). There was no association between HCPs' training on nutrition and receiving external supervision in the last six months with quality nutrition service provision in the adjusted analysis. There was an association between HCP-client communication (aIRR: 1.14; 95% CI: 1.04-1.26) and the use of visual aids or ANC cards (aIRR: 1.18; 95% CI: 1.11-1.27) with quality nutrition service provision. Finally, the clients in the second and third trimesters of pregnancy received higher-quality nutrition service (aIRR: 1.23, 95% CI: 1.11-1.35 and aIRR: 1.19, 95% CI: 1.08-1.32, respectively) compared to those in the first trimester.

Table 3. Unadjusted and adjusted incidence rate ratio of quality nutrition service at ANC according to health facility readiness, HCP characteristics, client-provider interactions and client characteristics

Variables	Unadjusted models		Adjusted model	
	IRR (95% CI)	p-value	aIRR (95% CI)	p-value
**Facility characteristics and readiness**				
All structural readiness[1] available	0.86 (0.77-0.96)	0.007	1.00 (0.98-1.03)	0.884
Logistical readiness quantile[2] (ref: Level 1 (poor))		0.015		0.013
Level 2 (average)	1.23 (1.04-1.45)	0.016	1.17 (1.04-1.32)	0.011
Level 3 (good)	1.31 (1.11-1.54)	0.001	1.23 (1.08-1.39)	0.002
Level 4 (best)	1.27 (1.03-1.58)	0.024	1.10 (0.95-1.28)	0.201
Average number of ANC services provided per day (ref: <5)		0.107		0.302
5-10	0.92 (0.81-1.05)	0.220	0.98 (0.89-1.07)	0.614
≥11	0.85 (0.73-0.99)	0.039	0.91 (0.82-1.02)	0.123
**Provider characteristics**				
Age of provider (years)	1.00 (0.99-1.00)	0.447		
Sex of provider (ref: female)	0.99 (0.84-1.17)	0.919		
Duration of work at this facility (years) (ref: ≤3)		0.062		0.731
4-7	1.16 (1.02-1.32)	0.024	1.04 (0.93-1.16)	0.537
≥8	1.13 (1.00-1.27)	0.055	1.05 (0.93-1.18)	0.464
Type of provider (ref: physician)		<0.001		<0.001
Nurse	0.99 (0.80-1.21)	0.900	0.89 (0.74-1.05)	0.169
Paramedic	1.41 (1.17-1.69)	<0.001	1.23 (1.06-1.42)	0.006
CHCP/CHW	1.46 (1.21-1.76)	<0.001	1.32 (1.12-1.57)	0.001
Received any training on nutrition	1.11 (1.00-1.24)	0.060	1.00 (0.91-1.10)	0.949
Knowledge on nutrition service (score out of 6)	1.07 (1.03-1.11)	0.001	1.04 (1.01-1.08)	0.012
Received external supervision in last 6 months	1.17 (1.01-1.36)	0.034	1.09 (0.97-1.24)	0.154
**HCP-client interaction (process)**				
HCP-client communication[3]	1.24 (1.11-1.38)	<0.001	1.14 (1.04-1.26)	0.007
Used visual aids or ANC card	1.19 (1.10-1.28)	<0.001	1.18 (1.11-1.27)	<0.001
**Client characteristics**				
Age of women (years)	1.00 (0.99-1.00)	0.169	1.00 (0.99-1.01)	0.946
Education (years of schooling)	1.00 (0.99-1.01)	0.781		
Gravida (ref: 1)		0.184		0.188
2	1.01 (0.96-1.05)	0.784	1.00 (0.96-1.05)	0.841
≥3	0.95 (0.90-1.01)	0.127	0.94 (0.87-1.02)	0.149
Gestational age (ref: 1st trimester)		<0.001		<0.001
2nd trimester	1.28 (1.16-1.42)	<0.001	1.23 (1.11-1.35)	<0.001
3rd trimester	1.23 (1.10-1.37)	<0.001	1.19 (1.08-1.32)	0.001
Number of ANC visits (ref: 1st visit)		0.234		
2nd – 3rd	1.05 (0.99-1.11)	0.108		
≥4th	1.04 (0.99-1.09)	0.156		

[1] Structural readiness: availability of examination room, privacy, examination bed, electricity and functioning toilet for clients**;** [2]Logistical readiness quantile was derived by categorizing score from principal component analysis of the availability of weighing scale, haemoglobin testing tool, health education materials/visual aids, ANC card, ANC guideline, ANC register and IFA supplement; [3]HCP discussing the progress of pregnancy, asked if the client had any

question; IRR: incidence rate ratio, aIRR: adjusted incidence rate ratio, ANC: antenatal care, CHCP: community health care providers, CHW: community health workers; HCP: health care provider, ref: reference

We also explored facility, HCP and client-level factors influencing each of the components of nutrition services at ANC (Table 4 and Table 5). The key influencing factors for weight measurement were the logistical readiness of the health facilities, clients seeing a lower-level provider like a paramedic or CHCP/CHW, using visual aids or ANC cards during service provision, and clients attending health facilities in the second and third trimester (Table 4). Anaemia assessment was also positively associated with higher HCPs' knowledge on nutrition and the use of visual aids or ANC cards but inversely associated with providers receiving external supervision. For IFA provision, the key influencing factors included lower-level HCPs like CHWs, clients in the second and third trimester of pregnancy, external supervision in the last six months, and HCP-client communication (Table 5). Provision of nutrition counselling was lower at facilities with 11 or more ANC services provided daily, but higher among community-level providers and HCPs with better knowledge of nutrition services. The use of visual aids or ANC cards and good HCP-client communication during the consultation also resulted in greater provision of nutrition counselling at ANC.

Table 4. Adjusted incidence rate ratio of weight measurement and anaemia assessment at ANC according to health facility readiness, HCP characteristics, client-provider interactions and client characteristics

Variables	Weight measured		Anaemia[1] assessed	
	aIRR (95% CI)	p-value	aIRR (95% CI)	p-value
**Facility characteristics**				
All structural readiness[1] available	1.00 (0.97-1.04)	0.876	1.03 (0.96-1.10)	0.483
Logistical readiness quantile[3] (ref: Level 1 - poor)		0.002		0.386
Level 2 (average)	1.61 (1.22-2.12)	0.001	0.94 (0.67-1.33)	0.749
Level 3 (good)	1.59 (1.21-2.11)	0.001	1.18 (0.87-1.60)	0.276
Level 4 (best)	1.39 (1.03-1.87)	0.030	0.94 (0.63-1.40)	0.761
Average number of ANC services provided per day (ref: <5)		0.699		0.163
5-10	1.00 (0.86-1.16)	0.999	1.06 (0.81-1.38)	0.668
≥11	1.06 (0.92-1.23)	0.456	0.78 (0.57-1.07)	0.130
**Provider characteristics**				
Duration of work in facility (years) (ref: ≤3)		0.486		0.334
4-7	1.07 (0.90-1.28)	0.429	1.17 (0.87-1.57)	0.298

Variables	Weight measured		Anaemia[1] assessed	
	aIRR (95% CI)	p-value	aIRR (95% CI)	p-value
≥8	1.11 (0.94-1.30)	0.212	0.95 (0.73-1.24)	0.712
Type of provider (ref: Physician)		0.001		0.132
Nurse	0.94 (0.76-1.18)	0.615	0.77 (0.51-1.17)	0.222
Paramedic	1.23 (1.01-1.49)	0.042	1.07 (0.80-1.43)	0.658
CHCP/CHW	1.32 (0.99-1.74)	0.055	0.76 (0.50-1.16)	0.202
Received training on nutrition	0.95 (0.85-1.06)	0.327	1.02 (0.76-1.37)	0.882
Knowledge on nutrition service (score out of 6)	0.98 (0.93-1.03)	0.404	1.18 (1.08-1.29)	<0.001
Received external supervision in last 6 months	1.00 (0.86-1.17)	0.959	0.77 (0.60-0.99)	0.042
**HCP-client interaction (process)**				
HCP-client communication[4]	1.06 (0.92-1.20)	0.427	0.87 (0.69-1.09)	0.222
Used visual aids or ANC card	1.23 (1.11-1.37)	<0.001	1.28 (1.08-1.53)	0.005
**Client characteristics**				
Age of women (years)	1.01(1.00-1.01)	0.144	1.00 (0.98-1.01)	0.546
Gravida (ref: 1)		0.084		0.927
2	1.02 (0.96-1.08)	0.546	0.99 (0.88-1.13)	0.907
≥3	0.92 (0.82-1.02)	0.120	1.03 (0.87-1.20)	0.790
Gestational age (ref: 1st trimester)		0.026		0.128
2nd trimester	1.19 (1.05-1.33)	0.005	1.03 (0.85-1.25)	0.763
3rd trimester	1.19 (1.04-1.35)	0.009	1.14 (0.93-1.38)	0.206

[1]Anaemia assessment included HCPs assessing blood haemoglobin levels or examining eye or palm (clinical assessment); [2] Structural readiness: availability of examination room/area, privacy, examination bed, electricity and functioning toilet for clients**;** [3]Logistical readiness quantile was derived by categorizing score from principal component analysis of the availability of weighing scale, haemoglobin testing tool, health education materials/visual aids, ANC card, ANC guideline, ANC register and IFA supplement; [4]HCP discussing the progress of pregnancy, asked if the client had any question; CHCP: community health care providers, CHW: community health workers; HCP: health care provider, aIRR: adjusted incidence rate ratio

Table 5. Adjusted incidence rate ratio of IFA provision and nutrition counselling at ANC according to health facility readiness, HCP characteristics, client-provider interactions and client characteristics.

Variables	IFA provided		Nutrition counselling	
	aIRR (95% CI)	p-value	aIRR (95% CI)	p-value
**Facility characteristics**				
All structural readiness[1] available	0.97 (0.91-1.04)	0.383	1.00 (0.94-1.06)	0.996
Logistical readiness quantile[3] (ref: Level 1 - poor)		0.410		0.236
Level 2 (average)	0.83 (0.61-1.13)	0.253	1.29 (0.96-1.75)	0.095
Level 3 (good)	0.81 (0.62-1.05)	0.108	1.35 (1.00-1.82)	0.047
Level 4 (best)	0.84 (0.61-1.17)	0.306	1.25 (0.90-1.74)	0.190
Average number of ANC services provided per day (ref: <5)		0.799		0.045
5-10	0.93 (0.72-1.18)	0.538	0.89 (0.72-1.10)	0.271
≥11	1.01 (0.73-1.39)	0.950	0.70 (0.53-0.94)	0.016
**Provider characteristics**				

Variables	IFA provided		Nutrition counselling	
	aIRR (95% CI)	p-value	aIRR (95% CI)	p-value
Duration of work in facility (years) (ref: ≤3)		0.454		0.471
4-7	0.81 (0.58-1.14)	0.235	1.15 (0.88-1.51)	0.302
≥8	0.94 (0.68-1.33)	0.779	1.18 (0.89-1.56)	0.260
Type of provider (ref: Physician)		0.023		0.029
Nurse	0.87 (0.51-1.46)	0.594	0.88 (0.57-1.38)	0.586
Paramedic	1.41 (0.99-2.00)	0.058	1.28 (0.89-1.85)	0.182
CHCP/CHW	1.74 (1.19-2.56)	0.005	1.56 (1.03-2.34)	0.034
Received training on nutrition	1.11(0.88-1.40)	0.357	1.01(0.85-1.20)	0.886
Knowledge on nutrition service (score out of 6)	0.99 (0.91-1.07)	0.745	1.08(1.01-1.16)	0.040
Received external supervision in last 6 months	2.42(1.42-4.11)	0.001	1.00(0.75-1.31)	0.949
**HCP-client interaction (process)**				
HCP-client communication[4]	1.34 (1.06-1.70)	0.013	1.51 (1.17-1.95)	0.002
Used visual aids or ANC card	1.05 (0.90-1.22)	0.571	1.20 (1.01-1.42)	0.038
**Client characteristics**				
Age of women (years)	1.00(0.99-1.01)	0.955	1.00(0.98-1.01)	0.675
Gravida (ref: 1)		0.239		0.432
2	1.06 (0.96-1.15)	0.245	0.96 (0.87-1.06)	0.443
≥3	0.96 (0.84-1.11)	0.599	0.90 (0.76-1.06)	0.219
Gestational Age (ref: 1st trimester)		<0.001		0.360
2nd trimester	2.13 (1.58-2.88)	<0.001	0.92 (0.79-1.09)	0.354
3rd trimester	1.84 (1.35-2.51)	<0.001	0.89 (0.75-1.05)	0.164

[1]Anaemia assessment included HCPs assessing blood haemoglobin levels or examining eye or palm (clinical assessment); [2] Structural readiness: availability of examination room/area, privacy, examination bed, electricity and functioning toilet for clients**;** [3]Logistical readiness quantile was derived by categorizing score from principal component analysis of the availability of weighing scale, haemoglobin testing tool, health education materials/visual aids, ANC card, ANC guideline, ANC register and IFA supplement; [4]HCP discussing the progress of pregnancy, asked if the client had any question; CHCP: community health care providers, CHW: community health workers; HCP: health care provider, aIRR: adjusted incidence rate ratio

Discussion

ANC is the formal point of contact for pregnant women to receive preventive health and nutrition services. We found that only 15% of women received all four nutrition services at ANC. Anaemia measurement and nutrition counselling were the least provided (52%) nutrition service. Logistical readiness of the facilities, types of ANC provider, HCPs' knowledge of nutrition services, client-provider interactions during the consultation and gestational age of clients were the factors influencing quality nutrition services during ANC. We found limited associations of training and external supervision of the providers with the quality

of nutrition services. Our study adds to the knowledge about the quality of nutrition services provided during pregnancy. It provides some critical insights on areas to intervene for improving the nutrition service provision at ANC in a low-resource setting.

Our findings of the quality of nutrition service provision at ANC is consistent with the poor content of ANC services reported in Bangladesh and other low-income settings [11, 17, 20]. However, previous studies mostly focused on the content of ANC, asking women who had recently given birth if they had received any of the specified services at any point during their pregnancy, rather than on a specific service contact [11, 15, 20]. Studies from Peru and Tanzania observing-ANC service at the point-of-care reported low adherence to recommended service provision [19, 21]. A study in Bangladesh also reported high weight measurement and IFA provision but inadequate counselling on nutrition at ANC [13].

Adequate readiness of the health facilities is essential to ensure quality service provision [11, 33-35]. Our study showed that improved logistical readiness of health facilities is strongly associated with higher quality nutrition services provision at ANC. In the disaggregated analysis, we found weight assessment was substantially lower in the facilities with poor logistical readiness compared to the service at better equipped facilities. However, the provision of IFA did not differ significantly by the facilities' logistical readiness level as IFA was available in most (93%) of the facilities. Although unadjusted analysis showed an inverse association between infrastructural readiness of facilities and quality nutrition service provision, this association was attenuated and no longer significant in the adjusted analysis. The crude association was likely to be confounded by the higher-tier facilities with better infrastructural readiness but poorer service provision, as shown in our descriptive analysis. Moreover, lower-level providers who mainly offer services at lower-tier facilities provided better quality service than physicians and nurses at higher-tier facilities. Previous studies from LMICs also reported that lower-level providers had offered similar or better compliance with standard maternal and child out-patient care than providers at higher-

level facilities [22, 36]. The National Nutrition Services programme should ensure a consistent supply of the logistics and supplies that are essential for nutrition service provision at ANC to improve the quality of service. The role of the providers at the higher level facility should be clarified based on WHO and national recommendations for nutrition services during pregnancy.

Our study demonstrates that HCP-client interactions such as informing them about the progress of pregnancy and giving them the opportunity to ask questions had a significant association with the quality of nutrition service provision. The WHO's standards for maternal and newborn care and WHO's guidelines on ANC recommend treating clients with dignity and compassion and providing information on health status and potential interventions for making an informed choice by clients [9, 37]. Earlier studies also reported the importance of HCPs' interpersonal communication and attitude towards clients to improve the quality of ANC services at health facilities [16, 18]. A study in Laos reported that HCP behaviour, mutual communication and treating clients with dignity are important considerations to improve ANC service quality [16]. This evidence highlights the importance of client-provider interactions for improving the quality of ANC nutrition services.

We also found that the application of job aids such as visual aids and ANC cards during ANC consultations improved the quality of nutrition services, especially for weight measurement, anaemia assessment, and nutrition counselling. A possible reason could be that HCPs document the key components of nutrition services provided on the ANC card, which acts as a reminder to ensure providing the services [38]. Similarly, visual aids likely remind HCPs of the counselling contents, improved communication techniques and service provision [39]. However, there was no association between the use of ANC cards or visual aids and increased provision of IFA supplements. The ANC card used in public facilities in Bangladesh does not mention IFA provision. Moreover, IFA provision is more likely to depend on the availability of the supplements, HCPs perceptions about supplementation, and client characteristics.

In contrast to previous studies, we did not find any association between HCPs nutrition training and the quality of nutrition service delivery during ANC [21, 40]. The content and quality of their training in nutrition might explain this finding. There are several criticisms of the government's Basic Nutrition Training that most HCPs received. There was too much content to cover in a short training time[41]. It was overly focused on child feeding with few practical demonstrations. The training quality declined when it was scaled up to a large number of HCPs nationwide using a cascade model [41, 42]. Only IFA provision was positively associated with receiving external supervision. Like training on nutrition, the quality of supervision visits to HCPs is often a question in resource-poor settings. HCPs often receive external supervision from administrative supervisors who have limited technical expertise and supervisory skills, resulting in a missed opportunity to reinforce the implementation of the knowledge and training obtained by HCPs into practice [41, 43]. Considering the quality issue of nutrition training, the current NNS operation plan introduced Comprehensive Competency-Based Training on Nutrition in 2017, which is implemented by technical experts [10]. The Competency-Based Training also addressed the supervision issue by developing and training supervisors on specifically designed modules to improve the technical competence of the supervisors. However, the success of Competency-Based Training in overcoming these challenges is yet to be evaluated.

Gestation was an important factor influencing the quality of nutrition services during ANC. Women coming for ANC in late trimesters received better quality nutrition services compared to women presenting in the first trimester [13, 22, 44]. The women presenting in the first trimester were less likely to have their weight assessed or receive IFA supplements. Low IFA supplement provision in the first trimester could be related to some inconsistent perceptions among HCPs on when to start IFA and the adverse effect of iron on foetal development [45, 46]. HCPs perception of the importance of different services and self-prioritisation of some services may also differ according to clients' gestational age. A study in Ghana suggested that HCPs sometimes disregard physical measurements in the first trimester

[22]. However, the reasons for lower compliance with weight assessment in the earlier stage of pregnancy require in-depth research. Detection and management of anaemia and provision of IFA early in pregnancy are important to avoid anaemia's detrimental effect on maternal and child health outcomes [47]. Adaptation of the national ANC guideline to comply with the global recommendations and improving HCPs' awareness of the recommended nutrition services during ANC visits may improve the quality of services.

The main strength of our study is the collection of observation-based data on nutrition service provision during ANC and the assessment of facility, provider and client characteristics at the same time-point, which allowed us to undertake supply-side and client-level factor analysis. The sampling of facilities covered 21 out of 64 districts spread across all geographic regions, which improved the generalisability of the study findings to public facilities in Bangladesh. Our study has some limitations. First, the observation of service provision by an external assessor may have resulted in some improved practice by HCPs. However, we anticipate that such bias will have a limited effect on the analysis of factors influencing the quality of service as facility and HCPs characteristics will not change during the observation. To reduce the Hawthorne effect, we conducted the observations in a natural setting, spending a reasonable time (i.e., one-two full days) at each facility [48]. We assured HCPs about their anonymity when presenting findings and emphasised that our observations were not related to individual performance evaluation. Second, the study methodology did not involve any in-depth interviews with HCPs to explore the reasons for the relatively low proportion of pregnant women whose weight was assessed during the first trimester of pregnancy and the limited impact of training and supervision on quality service provision. Third, we did not account for hospital management and governance level factors influencing the quality of nutrition service provision at health care platforms such as ANC. Fourth, we assessed only the on-site availability of rapid haemoglobin testing kits at the ANC room, which may underestimate the haemoglobin testing

readiness of district and sub-district hospitals as some of them might have advanced diagnostic tests for anaemia. Future research should explore these questions.

Conclusion

ANC is the primary platform to deliver nutrition services during pregnancy. The quality of nutrition service provided at ANC in public health facilities in Bangladesh is sub-standard. On the contrary to WHO and national recommendations, we observed differential practice in nutrition service provision by gestational age of the clients and type of HCPs proving the service necessitating increased awareness of ANC standards and guidelines among HCPs. Logistical readiness of the facility influences the quality of nutrition service provision and demands urgent attention from nutrition program managers. Better HCP-client communications, such as providing the ANC clients with information about the progress of pregnancy and allowing them to ask questions were associated with improved quality of the service. The utilisation of simple but effective tools, such as ANC cards and visual aids, resulted in better nutrition services and should be promoted universally. Failure of the traditional hierarchical administrative-focused supervision to improve the quality of nutrition service provision calls for service-specific supportive supervision by technical experts to harness the benefit of supervision.

Acknowledgments

We thank the Directorate General Health Services, Directorate General Family Planning and National Nutrition Services of the Ministry of Health and Family Welfare for approving us to conduct the study at public health facilities. We acknowledge the contribution of physicians and research assistants involved in the assessment. We are grateful to all participants for allowing us to conduct the assessment and interviews. We would like to thank Julie Ghostlaw, a program manager at IFPRI, for her support to edit

the manuscript. icddr,b is also grateful to the Governments of Bangladesh, Canada, Sweden and the UK for providing core/unrestricted support.

References

1. Victora CG, Christian P, Vidaletti LP, Gatica-Domínguez G, Menon P, Black RE. Revisiting maternal and child undernutrition in low-income and middle-income countries: variable progress towards an unfinished agenda. The Lancet. 2021.
2. Christian P, Mullany LC, Hurley KM, Katz J, Black RE. Nutrition and maternal, neonatal, and child health. Seminars in perinatology. 2015;39(5):361-72.
3. National Institute of Population Research and Training (NIPORT), Mitra and Associates, and ICF International. Bangladesh Demographic and Health Survey 2014. Dhaka, Bangladesh, and Rockville, Maryland, USA: NIPORT, Mitra and Associates, and ICF International, 2016.
4. National Institute of Population Research and Training (NIPORT), Mitra and Associates, ICF. Bangladesh Demographic and Health Survey 2017-18: Key Indicators. Dhaka, Bangladesh, and Rockville, Maryland, USA: National Institute of Population Research and Training (NIPORT), ICF, 2019.
5. Arsenault JE, Yakes EA, Islam MM, Hossain MB, Ahmed T, Hotz C, et al. Very low adequacy of micronutrient intakes by young children and women in rural Bangladesh is primarily explained by low food intake and limited diversity. The Journal of nutrition. 2013;143(2):197-203.
6. Abera SF, Kantelhardt EJ, Bezabih AM, Tsadik M, Lauvai J, Ejeta G, et al. What factors are associated with maternal undernutrition in eastern zone of Tigray, Ethiopia? Evidence for nutritional well-being of lactating mothers. BMC public health. 2020;20(1):1-12.
7. Kuhnt J, Vollmer S. Antenatal care services and its implications for vital and health outcomes of children: evidence from 193 surveys in 69 low-income and middle-income countries. BMJ open. 2017;7(11):e017122.
8. Haileslassie K, Mulugeta A, Girma M. Feeding practices, nutritional status and associated factors of lactating women in Samre Woreda, South Eastern Zone of Tigray, Ethiopia. Nutrition journal. 2013;12(1):1-11.
9. World Health Organization. WHO recommendations on antenatal care for a positive pregnancy experience: WHO; 2016.
10. GoB. 4th Health, Population and Nutrition Sector Programme (4th HPNSP), Operational Plan, National Nutrition Service (NNS) Institute of Public Health Nutrition, Directorate General of Health Service, Ministry of Health and Family Welfare, Government of the People's Republic of Bangladesh, 2017.
11. Islam MM, Masud MS. Determinants of frequency and contents of antenatal care visits in Bangladesh: Assessing the extent of compliance with the WHO recommendations. PloS one. 2018;13(9):e0204752.
12. Nguyen PH, Khương LQ, Pramanik P, Billah SM, Menon P, Piwoz E, et al. Effective coverage of nutrition interventions across the continuum of care in Bangladesh: insights from nationwide cross-sectional household and health facility surveys. BMJ open. 2021;11(1):e040109.
13. Billah SM, Saha KK, Khan ANS, Chowdhury AH, Garnett SP, Arifeen SE, et al. Quality of nutrition services in primary health care facilities: Implications for integrating nutrition into the health system in Bangladesh. PloS one. 2017;12(5):e0178121-e. doi: 10.1371/journal.pone.0178121. PubMed PMID: 28542530.
14. Donabedian A. The quality of care: How can it be assessed? JAMA. 1988;260(12):1743-8.
15. Afulani PA, Buback L, Essandoh F, Kinyua J, Kirumbi L, Cohen CR. Quality of antenatal care and associated factors in a rural county in Kenya: an assessment of service provision and experience dimensions. BMC health services research. 2019;19(1):1-16.

16. Phommachanh S, Essink DR, Jansen M, Broerse JE, Wright P, Mayxay M. Improvement of Quality of Antenatal Care (ANC) Service Provision at the Public Health Facilities in Lao PDR: Perspective and Experiences of Supply and Demand Sides. BMC pregnancy and childbirth. 2019;19(1):1-13.
17. Kanyangarara M, Munos MK, Walker N. Quality of antenatal care service provision in health facilities across sub–Saharan Africa: Evidence from nationally representative health facility assessments. Journal of global health. 2017;7(2).
18. Emiru AA, Alene G, Debelew GT. Women's satisfaction with the quality of antenatal care services rendered at public health facilities in Northwest Ethiopia: the application of partial proportional odds model. BMJ open. 2020;10(9):e037085.
19. Wynne SJ, Duarte R, de Wildt G, Meza G, Merriel A. The timing and quality of antenatal care received by women attending a primary care centre in Iquitos, Peru: A facility exit survey. PloS one. 2020;15(3):e0229852.
20. Singh L, Dubey R, Singh S, Goel R, Nair S, Singh P. Measuring quality of antenatal care: a secondary analysis of national survey data from India. BJOG: An International Journal of Obstetrics & Gynaecology. 2019;126:7-13.
21. Bintabara D, Nakamura K, Ntwenya J, Seino K, Mpondo BC. Adherence to standards of first-visit antenatal care among providers: A stratified analysis of Tanzanian facility-based survey for improving quality of antenatal care. PloS one. 2019;14(5):e0216520.
22. Amoakoh-Coleman M, Agyepong IA, Zuithoff NP, Kayode GA, Grobbee DE, Klipstein-Grobusch K, et al. Client factors affect provider adherence to clinical guidelines during first antenatal care. PLoS One. 2016;11(6):e0157542.
23. Goudet S, Murira Z, Torlesse H, Hatchard J, Busch-Hallen J. Effectiveness of programme approaches to improve the coverage of maternal nutrition interventions in South Asia. Maternal & child nutrition. 2018;14 Suppl 4(Suppl 4):e12699-e. doi: 10.1111/mcn.12699. PubMed PMID: 30499258.
24. Billah SM, Arifeen SE. Evaluation of GoB, UNICEF, CIFF initiative to facilitate and accelerate implementation of the Bangladesh National Nutrition Services Registry for International Development for Impact Evaluations (RIDIE), Available at: 10.23846/ridie0902016. Available from: https://ridie.3ieimpact.org/index.php?r=search/detailView&id=415.
25. Billah SM, Khan ANS, Raihana S, Rahman M, Salam SS, Arifeen SE, et al. Evaluation of Accelerating Implementation of the Bangladesh National Nutrition Services: Initial Assessment 2016. 2016.
26. Campbell SM, Roland MO, Buetow SA. Defining quality of care. Social science & medicine. 2000;51(11):1611-25.
27. Bruce J. Fundamental elements of the quality of care: a simple framework. Studies in family planning. 1990;21(2):61-91.
28. NIPORT, ACPR, ICF International. Bangladesh Health Facility Survey 2014. Dhaka, Bangladesh: National Institute of Population Research and Training (NIPORT), Associates for Community and Population Research (ACPR) and ICF International, 2016.
29. MOH&FW. Maternal Health Standard Operating Procedure (SOP) Volume-1. Bangladesh: Ministry of Health and Family Welfare, 2015.
30. NIPORT, Mitra and Associates, and ICF International. Bangladesh Demographic and Health Survey 2014. . Dhaka, Bangladesh, and Rockville, Maryland, USA:: National Institute of Population Research and Training (NIPORT), Mitra and Associates, and ICF International., 2016.
31. Owili PO, Muga MA, Mendez BR, Chen B. Quality of maternity care and its determinants along the continuum in Kenya: A structural equation modeling analysis. PloS one. 2017;12(5):e0177756.
32. Harris T, Yang Z, Hardin JW. Modeling underdispersed count data with generalized Poisson regression. The Stata Journal. 2012;12(4):736-47.

33. Mansur AM, Rezaul KM, Mahmudul HM. Quality of antenatal care in primary health care centers of bangladesh. Journal of family & reproductive health. 2014;8(4):175.
34. Rahman A, Nisha MK, Begum T, Ahmed S, Alam N, Anwar I. Trends, determinants and inequities of 4+ ANC utilisation in Bangladesh. Journal of Health, Population and Nutrition. 2017;36(1):2.
35. Stelfox HT, Straus SE. Measuring quality of care: considering measurement frameworks and needs assessment to guide quality indicator development. Journal of clinical epidemiology. 2013;66(12):1320-7.
36. Hoque DE, Arifeen SE, Rahman M, Chowdhury EK, Haque TM, Begum K, et al. Improving and sustaining quality of child health care through IMCI training and supervision: experience from rural Bangladesh. Health policy and planning. 2014;29(6):753-62.
37. World Health Organization. Standards for improving quality of maternal and newborn care in health facilities. Switzerland: WHO, 2016 9241511214.
38. Gross K, Schellenberg JA, Kessy F, Pfeiffer C, Obrist B. Antenatal care in practice: an exploratory study in antenatal care clinics in the Kilombero Valley, south-eastern Tanzania. BMC pregnancy and childbirth. 2011;11(1):1-11.
39. Jennings L, Yebadokpo AS, Affo J, Agbogbe M. Antenatal counseling in maternal and newborn care: use of job aids to improve health worker performance and maternal understanding in Benin. BMC pregnancy and childbirth. 2010;10(1):1-13.
40. Omer AM, Haile D, Shikur B, Macarayan ER, Hagos S. Effectiveness of a nutrition education and counselling training package on antenatal care: a cluster randomized controlled trial in Addis Ababa. Health policy and planning. 2020;35(Supplement_1):i65-i75. doi: 10.1093/heapol/czaa101. PubMed PMID: 33165586.
41. Saha KK, Billah M, Menon P, El Arifeen S, Mbuya NV. Bangladesh National Nutrition Services: Assessment of Implementation Status: World Bank Publications; 2015.
42. Ajeani J, Mangwi Ayiasi R, Tetui M, Ekirapa-Kiracho E, Namazzi G, Muhumuza Kananura R, et al. A cascade model of mentorship for frontline health workers in rural health facilities in Eastern Uganda: processes, achievements and lessons. Global health action. 2017;10(sup4):1345497.
43. Karuga RN, Mireku M, Muturi N, McCollum R, Vallieres F, Kumar M, et al. Supportive supervision of close-to-community providers of health care: Findings from action research conducted in two counties in Kenya. PloS one. 2019;14(5):e0216444.
44. Berehe TT, Modibia LM. Assessment of Quality of Antenatal Care Services and Its Determinant Factors in Public Health Facilities of Hossana Town, Hadiya Zone, Southern Ethiopia: A Longitudinal Study. Advances in Public Health. 2020;2020:NA-NA.
45. Alam A, Rasheed S, Khan NU, Sharmin T, Huda TM, Arifeen SE, et al. How can formative research inform the design of an iron-folic acid supplementation intervention starting in first trimester of pregnancy in Bangladesh? BMC public health. 2015;15(1):374.
46. Siekmans K, Roche M, Kung'u JK, Desrochers RE, De-Regil LMJM, nutrition c. Barriers and enablers for iron folic acid (IFA) supplementation in pregnant women. 2018;14:e12532.
47. Di Renzo GC, Spano F, Giardina I, Brillo E, Clerici G, Roura LC. Iron deficiency anemia in pregnancy. Women’s Health. 2015;11(6):891-900.
48. Oswald D, Sherratt F, Smith S. Handling the Hawthorne effect: The challenges surrounding a participant observer. Review of social studies. 2014;1(1):53-73.

Supporting information

S1 Table. Definition of outcome and explanatory variables and data collection methods

Figure 1 Click here to access/download;Figure;Fig 1.tif

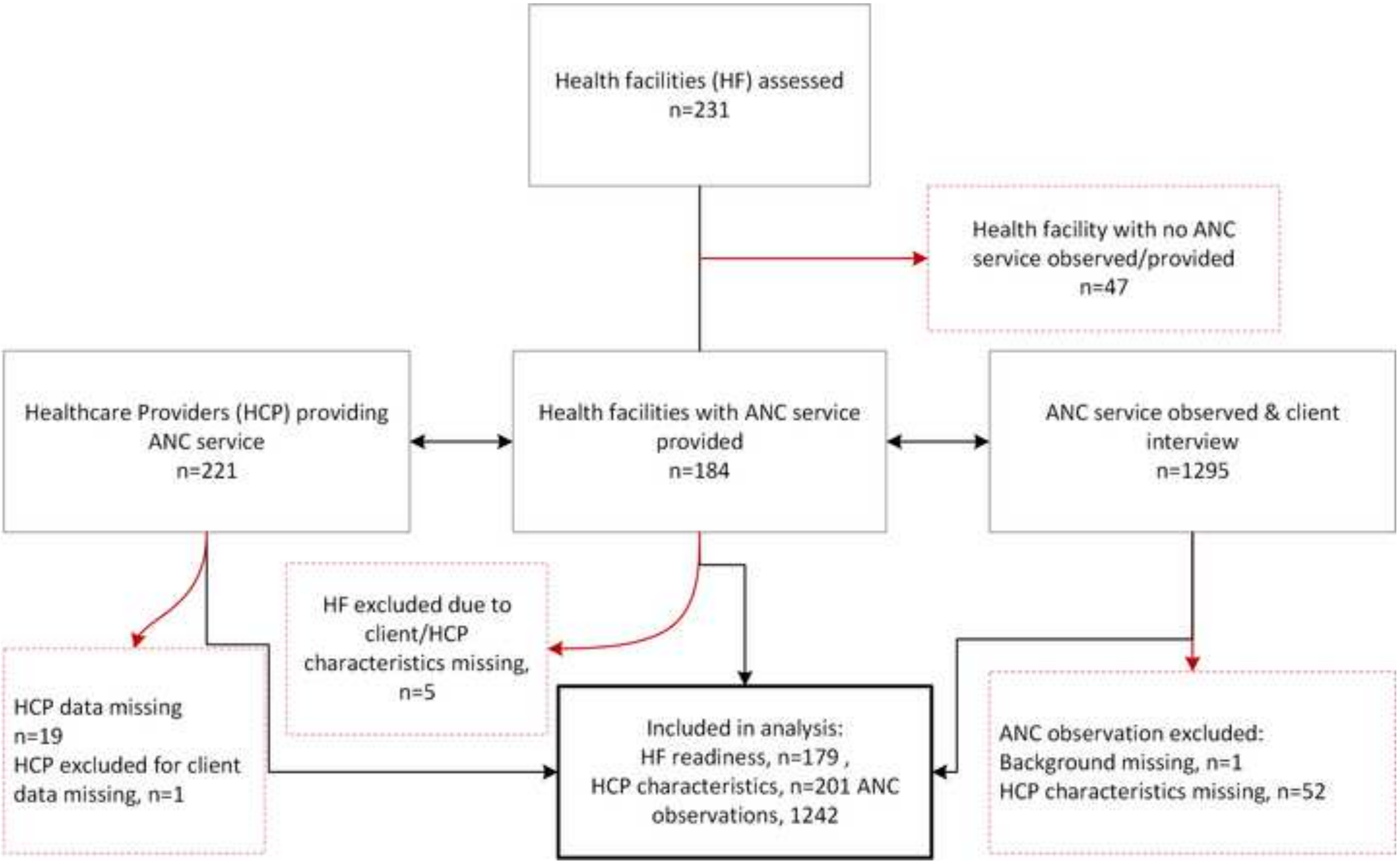

Click here to access/download;Figure;Fig 2.tif

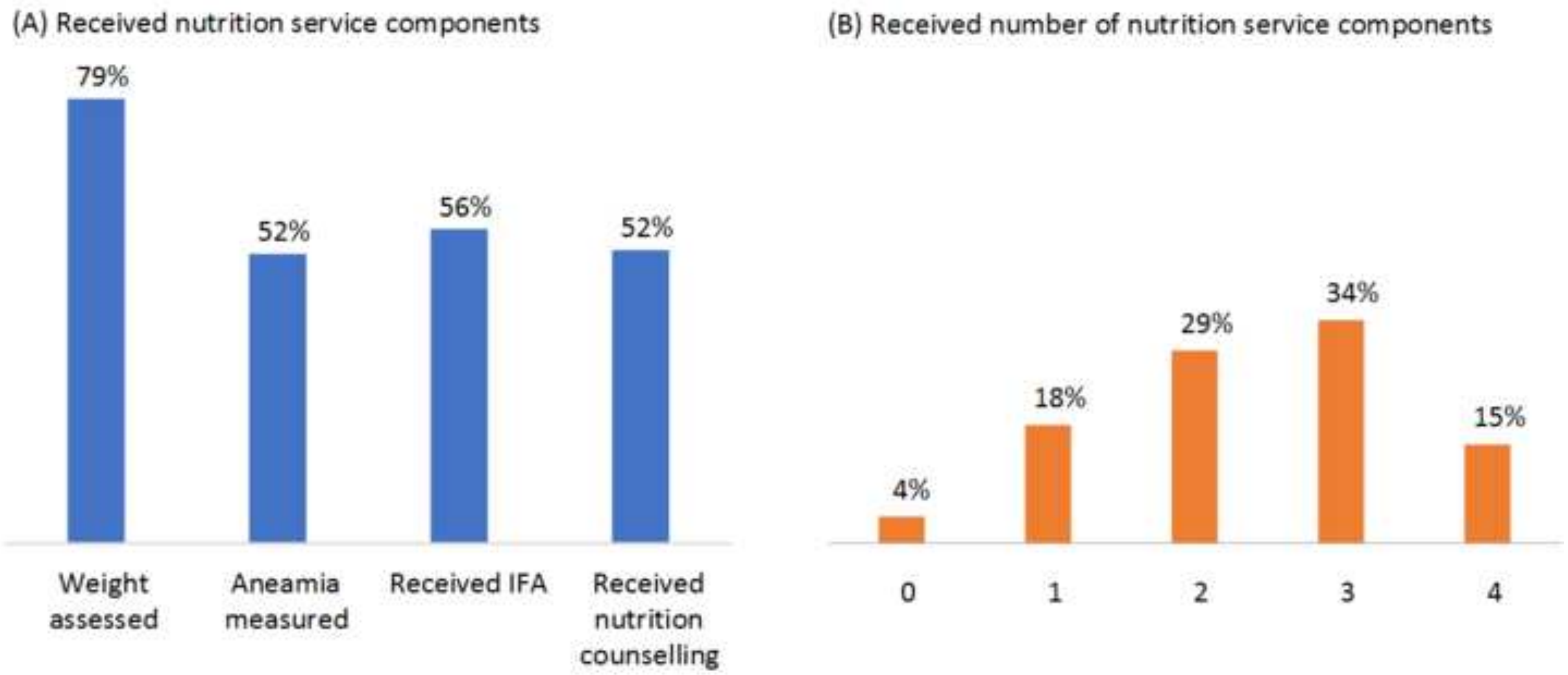

S1 Table

Click here to access/download
Supporting Information
S1_Table.docx

Factors influencing ~~Determinants of~~ quality nutrition service provision at antenatal care contacts: findings from a public health facility-based observational study in 21 districts of Bangladesh

Authors

Sk Masum Billah[1,2*], Nazia Binte Ali[1], Abdullah Nurus Salam Khan[1], Camille Raynes-Greenow[2], Patrick John Kelly[2], Md. Shahjahan Siraj[1], Sufia Askari[3], Purnima Menon[4], Shams El Arifeen[1], Michael John Dibley[2], Phuong Hong Nguyen[4]

[1]Maternal and Child Health Division, International Centre for Diarrhoeal Disease Research, Bangladesh (icddr,b), Dhaka, Bangladesh
[2]The University of Sydney School of Public Health, Sydney, NSW 2006, Australia
[3]Children's Investment Fund Foundation, Clifford Street. London W1S 2FT, United Kingdom
[4]International Food Policy Research Institute (IFPRI), Washington, DC., United States of America

* Corresponding author
E-mail: billah@icddrb.org

// Abstract

Malnutrition during pregnancy is associated with increased maternal morbidity and mortality and has a long-term negative impact on child growth and development. Antenatal care (ANC) is the formal point of contact for pregnant women to receive preventive health and nutrition services. We assessed the quality of nutrition service delivery during ANC and examined its influencing factors~~determinants~~ related to the health facility, health care provider (HCP) and client characteristics. We conducted a cross-sectional assessment in 179 facilities, including 1,242 ANC observations and exit interviews of ~~the~~ pregnant women from 21 districts in Bangladesh. We considered four essential nutrition services at each ANC contact including -maternal weight measurement, anaemia assessment, nutrition counselling and iron-folic acid (IFA) supplement provision. We defined a composite 'quality nutrition service' outcome -by- counting ~~adding~~ the ~~counts~~number -of ~~four~~ services (out of four) provided at each ANC from observation data~~, including maternal weight measurement, anaemia assessment, nutrition counselling and iron-folic acid (IFA) supplement provision~~. We explored both the supply--side and the client-level factors~~determinants~~ of quality nutrition service using multilevel Poisson regression. Overall, only 15% of clients received all four nutrition services. Performance of weight measurement (79%) was higher than IFA provision (56%), anaemia assessment (52%) and nutrition counselling (52%). The multivariable analysis showed that quality nutrition service delivery is positively associated with good logistical readiness of the facilities (aIRR: 1.23, 95% CI: 1.08-1.39), consultation by paramedics (aIRR 1.23, 95% CI: 1.06-1.42) and community health care providers (aIRR 1.32, 95% CI: 1.12-1.57), HCP's' knowledge on maternal nutrition (aIRR 1.04; 95% CI: 1.01-1.08), better HCP-client communication (aIRR 1.14; 95% CI: 1.04-1.26) and use visual aids or ANC card (aIRR 1.18; 95% CI: 1.11-1.27). We found limited associations ~~of~~ between HCP~~'s~~ training and external supervision with the quality of nutrition services. In conclusion, t~~T~~he quality of nutrition service provision during ANC is suboptimal. Public health nutrition programmers should ensure the facilities' logistical

readiness, and revisit~~s~~ and reinforce the content and modality of training and supportive supervision of the HCPs. They should also emphasize positive HCP-client communication and the use of job aids to improve the quality of nutrition service provision during ANC.

Introduction

Maternal nutrition is a major global public health concern. Undernutrition and micronutrient deficiencies during pregnancy are associated with increased maternal morbidity and mortality and have a long-~~-~~term negative impact on child growth and development [1, 2]. Despite gradual progress in low and middle-income countries (LMIC) during the last two decades, maternal and child nutrition improvements remain an unfinished agenda, especially with the interruptions by the COVID-19 pandemic [1]. In Bangladesh, 42% of women of reproductive age (15-49 years) and nearly half of all pregnant women are anaemic, 12% are thin~~,~~ and 14% have short stature [3, 4]. The principal drivers for maternal malnutrition are poor-quality diets, low intake of micronutrient supplements~~,~~ and lack of access to quality health~~-~~care during pregnancy [5, 6]. A comprehensive analysis of data from 69 LMICs suggested that receiving antenatal care (ANC) from appropriate sources and with recommended frequency was associated with positive birth outcomes~~-~~ and childhood nutrition [7]. Evidence also suggests receiving three or more ~~antenatal care~~ANC visits is likely to reduce maternal malnutrition by three-fold [8]. All these findings highlight the need for improving coverage and quality of nutrition services during pregnancy.

ANC is the first contact for pregnant women to health and nutrition services.~~ The~~ “WHO antenatal care guideline for positive pregnancy experience” recommends routine supplementation of iron-folic acid (IFA), nutrition counselling~~,~~ and weight monitoring during ANC to ensure greater coverage of nutrition interventions during pregnancy [9]. Aligned with global recommendations, the G~~g~~overnment of Bangladesh prioritized maternal nutrition service delivery using the ANC platform in their fourth Health Nutrition and Population Sector Program to continue ~~the~~ ‘~~nutrition~~ mainstreaming nutrition’ in~~to~~ the

health systems ~~approach~~ [10]. Like several LMICs, timely utilis~~z~~ation and quality of ANC remain a major challenge to increase the reach and coverage of nutrition interventions in Bangladesh [11]. In 2017, the coverage of four ANC visits was 47%, indicating more than half of pregnant women were missing the opportunity of maternal nutrition services [4]. Another challenge is ensuring the quality of nutrition interventions provided at ANC contacts~~care~~, without which programmes will not fully harness the benefits of these~~ nutrition~~ interventions on maternal and child nutrition outcomes. Moreover, a recent study ~~facilities have demonstrated~~ reported incomplete readiness of healthcare facilities to provide~~for~~ nutrition interventions during ANC (51%) [12]. Nutrition input-adjusted coverage was suboptimal (18% for ANC) and disproportionately affected the poor and women with lower educational attainment. Suboptimal quality of care for nutrition services during ANC further deteriorates the situation. An assessment of the quality of nutrition services in~~ the~~ primary care facilities showed that only 30% of mothers received four nutrition services during ANC [13].

The quality of health service delivery primarily depends on three ~~-~~levels of factors: structure (readiness of the health facilities), process (provision and experience of care)~~,~~ and outcomes (effective coverage and health outcomes) [14]. Previous studies from LMICs have mainly reported on the quality of ANC service by using one or some of the following indicators: frequency of visits, structural readiness to provide the service, content on maternity care, client experience~~,~~ and their determinants [11, 15-21]. Although~~,~~ most of these studies covered the structural and outcome-~~-~~level indicators, very few explored process-~~-~~level indicators. ~~-~~One of the few attempts in exploring process-level factors and adherence to recommended quality provisions during ANC was made in 2016 in Tanzania [21].~~–~~ The study reported that female providers, availability of routine tests and basic medicines at the public facilities and providers who received refresher training were positively associated with better adherence to quality ANC provision at a single point of contact. However, this study lacked~~s~~ the client-~~–~~level factor and experience of care domains. ~~On the contrary, A~~another study conducted in Ghana explored the client-~~-~~level factors for quality

ANC but lacked structural and provider-level factors [22]. ~~Moreover,~~ A~~a~~ recent systematic review (2018) looking at the effectiveness of nutrition programming~~,~~ highlighted the need for additional analysis on barriers and enablers of nutrition service delivery using ANC and maternal, newborn and child health platforms in South Asia [23]. Considering the existing evidence and current research needs, a comprehensive analysis of quality ANC service delivery including structural, process and client-~~-~~level factors, will be beneficial for effective programme planning [21]. This paper aims to assess the quality of nutrition service provision during ANC and examine its influencing factors ~~determinants~~ related to facility readiness, health care providers, the process of care, and client characteristics.

Methods

Study design and setting

This study is a part of an initial cross-sectional assessment of coverage and quality of maternal and child nutrition services for a health system strengthening project, ~~that aimed to~~ A~~a~~ccelerating~~e the~~ I~~i~~mplementation of the National Nutritional Services (AINNS) in Bangladesh. The project included interventions to improve coordination of nutrition service delivery both at the national and sub-national levels and health care provider~~'~~s' (HCP) skills for maternal and child nutrition service delivery at public health care platforms. Details of the project evaluation plan have been reported elsewhere [24, 25]. Briefly, the initial assessment was done in 21 out of 64 districts in Bangladesh. We selected public health facilities from 14 of 40 AINNS intervention districts and seven~~7~~ of 24 non-intervention districts.~~ ~~ We selected the districts based on comparability of population density, literacy rate, housing characteristics, people in the lowest wealth quintile, access to a safe drinking water source, electricity connection, improved sanitation, coverage of childhood immunization, ANC, skilled birth attendance, postnatal care and modern methods of family planning, under-five mortality rate and childhood stunting prevalence. We created a score for each district by principal component analysis of these variables. Then, we applied

nearest neighbour matching of the district's PCA score to match two intervention districts with one non-intervention district. Finally, we selected seven matched groups of districts (each having two AINNS intervention districts and one non-intervention), which had the minimum difference in the PCA score. ~~Before selection, we matched two intervention districts with one non-intervention district by a score created by principal component analysis of district's population, geographic, and health care characteristics.~~

The initial assessment included secondary to community-level public health facilities, namely district hospitals, sub-district hospitals, union health and family welfare centres and community clinics at the ward-level. At the district and sub-district-level hospitals, physicians with or without specialis~~z~~ation in obstetrics and gynaecology, nurses and paramedics provide maternal care services, including ANC. At union-level facilities, which serves a catchment of 25-30 thousand people, paramedics are the main ANC service providers. These paramedics have either a four-year medical diploma or 18 months of pre-service training including six months on midwifery skills. At community clinics, which serve six to eight thousand people in each ward, community health care providers and frontline community health workers provide ANC services. From each district, we selected the district~~s~~ hospital and two~~the~~ upazila health complexes (sub-district hospitals) from two randomly selected subdistricts for this assessment. We also randomly selected four union-level health facilities and four community clinics from a list of functioning facilities prepared in consultation with the sub-district health manager in each chosen sub-district. We conducted the initial assessment between February and June 2016.

Data collection

We used the structure and process domains of the Donabedian framework for the factors influencing ~~determinants of~~ quality nutrition service provision. Globally, the Donabedian framework is widely used for measuring the quality of healthcare service delivery [14, 26]. This framework identifies structure,

process~~,~~ and outcome level indicators for a comprehensive measurement of quality of care [26, 27]. We have also considered the client characteristics as they have been found to influence the provision of ANC services [21]. We ~~used mixed methods to~~ collected~~ different~~ data at both supply and client-~~-~~levels from different sources, including i~~1~~) a health facility readiness assessment, ii~~2~~) health care provider interviews, ~~3~~iii) direct observation of ANC practices, and iv~~4~~) exit interviews with the ANC clients. For health facility assessment, we used a structured checklist adapted from the Bangladesh Health Facility Assessment tool [28] to collect information on facility infrastructure, and availability of specific logistics items (including weighing scale, haemoglobin testing tool, health education material/visual aids, ANC card, clinical guideline, registers~~,~~ and iron-folic acid (IFA) supplements). ~~–~~Interviews of the ANC service providers included information about their duration of work in the facility, training status~~,~~ and knowledge regarding nutrition services. For direct observation, we developed the observation checklist following the standard operating procedures for maternal health services [29] to document the nutrition services provided at ANC consultation. Finally, we conducted exit interviews with clients after receiving ANC to collect information on their background, demographic and obstetric characteristics~~,~~ and ANC service care-seeking. S1 Table summarizes the outcome and explanatory measures, definitions~~,~~ and data collection methods.

Sample size

We ~~conducted the~~ assessed the~~ment~~ readiness to provide ANC services in 231 facilities (Fig 1). ANC services were not sought in 47 out of the 231 facilities on the assessment days; thus, we observed 1295 ANC service consultations~~,~~ ~~and in~~at 184 facilities.~~, we observed ANC services provided on the assessment days.~~ We had complete background information~~interviewed~~ from 201~~217 health care providers~~HCPs who offered ANC services ~~at the facilities~~ on the day of the visit~~, and we observed 1296 ANC consultations~~. We excluded 53~~4~~ observations from the analysis due to missing information on~~about the health care~~

~~providers~~HCPs or ~~, ~~the client~~'s~~ characteristics~~, or the exit interview (Fig 1)~~. Finally, ~~W~~we included~~conducted the final analysis among~~ 1,242 observations of ANC service consultation~~s~~ provided by 201 service providers at 179 facilities in the analysis for this paper.

Fig 1. Study flow diagram showing health facilities, health care providers and participants recruited and final sample size.

ANC: antenatal care, HCP: h~~H~~ealth care provider, HF: h~~H~~ealth facility

Measures of quality of nutrition services (o~~O~~utcome measures)

We considered maternal weight measurement, screening~~assessment~~ for anaemia, nutrition counselling and provision of IFA supplements as the ANC nutrition services [10]. During the observations of ANC, the assessors recorded if these services were provided. We considered weight assessment performed if the HCPs took the weight of pregnant women using either a digital or analogue weighing scale. We included anaemia assessment~~ in the measurement of the quality of nutrition service. It an essential service ~~in the quality of nutrition service during ANC~~, following the national guideline ~~due to the~~for a population with a~~ high prevalence of ~~-~~anaemia ~~burden ~~among women of reproductive age in Bangladesh [30]. ~~During the observations of ANC, the assessors recorded the services provided.~~ Screening for anaemia included assessing blood haemoglobin levels either using Tallquist paper onsite, by previous laboratory investigation or examining eye or palm (clinical assessment). Provision of nutrition counselling included HCPs providing messages on dietary diversity, quantity and types of nutritious food. We defined IFA provision as HCPs distributing IFA supplements during the ANC consultation; however, we did not include HCPs prescribing IFA to be bought from outside pharmacies. We created a composite score variable for 'quality nutrition service' by adding the number of services (out of four) provided at the ANC consultation. Similar methods of defining a composite score for quality service have been used in previous studies [11, 31].

Explanatory variables

We explored the supply-~~-~~side and the client-level factors ~~determinants~~ of quality nutrition services provided at ANC (S1 Table). According to the 'structure' domain of the Donabedian framework [14], we considered the health facility characteristics and readiness~~,~~ and HCP~~'s~~ characteristics and knowledge as supply-side factors~~determinants~~. At the health facility~~-~~level, we constructed two indicators. The first was structural readiness (if all five readiness items were available).~~ And~~ T~~t~~he ~~second was~~ logistical~~ s and supplies~~ readiness indicator was ~~(~~created from seven logistic and supplies items using ~~a~~ principal component analysis~~,~~ and then categorized into four logistical readiness quantiles~~)~~. Cronbach's alpha for the logistics and supplies included in the composite score calculation was 0.62. HCP~~'s~~ characteristics included age, sex, duration of service at the facility~~,~~ and receiving external supervision at least once in the last six months. HCP~~'~~s' knowledge of nutrition services at ANC was a composite score derived by adding the correct responses to four questions (S1 Table). The ~~'~~process~~'-~~-level factor~~determinants~~ included two variables on HCP-client interaction: ~~including~~ 'HCP-client communication' (HCP discussing the progress of pregnancy and asking if the client has any questions) and the 'use of visual aids and ANC card' during ANC service provision. Client-level factor~~determinant~~s included~~volved~~ client characteristics, namely age, education, gestational age~~,~~ and the number of ANC visits.

Statistical analysis

We used descriptive analysis to summarise the client and provider characteristics, health facility readiness to provide nutrition services at ANC, and client-provider interactions during ANC consultation. We reported summary statistics by frequency and proportion or by mean (±SD) for categorical and continuous variables, respectively. We explored the differences in provider characteristics and provider-client interactions by types of providers and facility readiness by types of facilities using Analysis of Variance (ANOVA) and chi-square test for continuous and categorical characteristics variables, respectively. We

checked for the linearity in the relationship between each continuous explanatory variable and ~~the quality~~ nutrition service quality score by fitting the locally estimated scatterplot smoothing (loess) curve. As the outcome variable~~,~~ includes counts of nutrition services,~~,~~ we used Poisson regressions to examine the potential influencing factor~~determinant~~s of quality nutrition service at ANC [32]. We included provider and facility as the random effects in the model as clients were nested within the providers and providers were nested within the facilities. We calculated p-values using Wald tests that had robust standard errors to adjust for model violations, such as under-dispersion. In the multivariable regression model, we included the explanatory variables associated with quality nutrition service at a p-value <0.2 in the bivariate analysis. The facility type had collinearity (correlation coefficient: 0.72) with HCP type. We compared Akaike's information criterion (AIC) and Bayesian information criterion (BIC) between the models with and without facility type and excluded facility type from the final model. We also ran multivariable Poisson regression using each of the four elements of quality nutrition services as the outcome. We reported the adjusted incidence rate ratios (aIRR) and their 95% confidence interval as the measure of association. Associations were considered statistically significant at a p-value<0.05 in the final model. We used Stata version 14 software in all statistical analyses.

Ethical approval

We obtained ethical approval from the Ethical Review Committee of icddr,b (Protocol Number-15107), and written informed consent from HCPs, health facility managers and ANC clients before data collection.

Results

The study includes data from 21 district hospitals, 40 sub-district hospitals, 61 union-~~-~~level facilities and 57 community clinics. Most of the facilities (88%) had one HCP who provided ANC services on the ~~visit~~ day of assessment and participated in the study. Overall, 58% of facilities had all five infrastructural readiness

items considered in this analysis (Table 1). Infrastructural readiness of community clinics (21%) and family welfare centres~~FWC~~ (56%) was~~ere~~ poorer than higher-tier facilities, including district hospitals (95%) and sub-district hospitals (91%). The overall availability of ~~logistics~~supplies like weighing scales (82%), ANC registers (99%), IFA supplements (93%) and visual aids for counselling (88%) was~~ere~~ high. However, only 35% of facilities had a haemoglobin testing kit to detect maternal anaemia and about one~~a~~ third had an ANC guideline. We found significant differences in the availability of haemoglobin testing kits~~tool~~, visual job aids, ANC cards and IFA supplements across different types of facilities (all p<0.01).

Table 1. Characteristics and readiness of the healthcare facilities by types of facility

	Overall (N=179)	District hospital (N=21)	Sub-district hospital (N=40)	Union health centre (N=61)	Community clinic (N=57)	p-value
**All structural readiness[1] items available**	57.5	90.5	95.0	55.7	21.0	<0.001
**Logistics and supplies**						
Weighing scale	82.1	81.0	72.5	85.3	86.0	0.318
Haemoglobin testing tool	35.2	9.5	17.5	50.8	40.4	<0.001
Health education materials/visual aids	88.3	81.0	72.5	93.4	96.5	0.001
ANC card	84.9	71.4	72.5	95.1	87.7	0.004
ANC guideline	30.2	38.1	30.0	32.8	24.6	0.642
ANC register	99.4	95.2	100	100	100	0.056
IFA supplement	93.3	95.2	97.5	83.6	100	0.002
**Average number of ANC services provided on a day~~ANC utilization per day~~**						
<5	44.7	9.5	42.5	57.4	45.6	<0.001
5-10	35.8	23.8	30.0	36.1	43.9	
≥11~~ or more~~	19.6	66.7	27.5	6.6	10.5	

Values are %; p-values are test of difference in facility readiness across facility types; [1] Structural readiness: availability of examination room/area, privacy, examination bed, electricity and functioning toilet for clients
ANC: antenatal care, IFA: iron-~~ and~~ folic acid

Most of the HCPs (86%) were female (Table 2). About~~ a~~ half of the community HCPs received nutrition training compared to only 9% of physicians. Knowledge of nutrition services was similar across the HCP types. Although 84% of HCPs received external supervision at least once in six months preceding the

assessment, the external supervision of nurses was much lower (55%) compared to other types of HCP (>80%). HCP-client interaction during ANC consultation varied significantly by types of providers (Table 2). While the proportion of HCPs informing clients about the progress of their pregnancy or allowing clients to ask questions was high (73%), visual aid~~s~~ or ANC card usage during counselling was low (only in 47% of ANC consultations). ~~On average, c~~Clients who received ANC from the facilities ~~were~~had a mean age of 23 years (SD±4.3 years) ~~old~~ and had a mean eight years (SD±2.9 years) of schooling. Clients were mostly first-time pregnant women (42%), in their second trimester (46%), and came for either the second or third ANC visit (44%) ~~(data not shown)~~.

Table 2. Characteristics of health care providers and provider-client interaction by types of health care provider

	Overall (N=201)	Physician (N=35)	Nurse (N=20)	Paramedics (N=88)	CHCP/CHW (N=58)	p-value
**HCP characteristics**						
Age of provider (years)	38.1 (±10.0)	35.7 (±6.8)	41.4 (±9.1)	43.1 (±10.2)	30.9 (±6.2)	<0.001
Sex of the provider						
Male	13.9	5.7	0	4.6	37.9	<0.001
Female	86.1	94.3	100	95.5	62.1	
Duration of work at the current facility (years)						
≤3	32.3	62.9	35.0	35.2	8.6	<0.001
4-7	37.8	25.7	15.0	15.9	86.2	
≥8 ~~or more~~	29.9	11.4	50.0	48.9	5.2	
Received any in-service training on nutrition	28.9	8.6	20.0	23.9	51.7	<0.001
Knowledge (score out of 6) on nutrition services at ANC[1]	4.3 (±1.4)	4.2 (±1.6)	4.3 (±1.1)	4.3 (±1.4)	4.3 (±1.4)	0.994
Received any external supervision in last 6 months	83.6	82.9	55.0	83.0	94.8	0.001
Place of work						
District hospital	13.9	40.0	30.0	9.1	0	<0.001
Sub-district hospital	26.9	60.0	70.0	21.6	0	
Union health centre	30.4	0	0	69.3	0	
Community clinic	28.9	0	0	0	100	
**HCP-client interaction**	**N=1242**	**N=338**	**N=179**	**N=497**	**N=228**	
HCP-client communication[2]	72.6	61.8	63.1	87.7	63.2	<0.001
Used visual aids or ANC card	47.0	29.9	71.5	56.3	32.9	<0.001

Values are % or mean (± SD)~~.~~, p-values test difference of ~~health care providers~~HCP characteristics by types of health care provider;~~,~~ [1]includes knowledge on the provision of Iron-~~-~~folic acid, diagnosis and management of anaemia, weight measurement and diet counselling; [2]HCP discussing ~~about~~ the progress of pregnancy, asked if the client had any question H~~P~~CP: health care providers, ANC: antenatal care, CHCP: c~~C~~ommunity h~~H~~ealth c~~C~~are p~~P~~roviders, CHW: c~~C~~ommunity h~~H~~ealth w~~W~~orkers

Among the four nutrition services to be provided during ANC, assessment of weight for maternal weight gain during pregnancy was the highest (79%), followed by IFA provision (56%) (Fig 2A). In nearly half of the ANC consultations, HCPs ~~assessed~~ screened for anaemia either by testing haemoglobin or by clinical examination of eye or palm and provided counselling on appropriate dietary practices. Overall, only 15% of clients received all four nutrition services components and about one~~a~~ third received three services (Fig 2B).

Fig 2. Percentage of clients received (A) nutrition service components and (B) number of nutrition service components at ANC.

IFA: Iron-~~-~~folic acid

We summarized the factors ~~determinants~~ influencing~~of~~ the overall quality of ANC nutrition services in Table 3. The unadjusted results ~~-~~showed that logistical readiness of the facilities, types of HCP, knowledge of the HCP on maternal nutrition, provision for external supervision, HCP-client communication, use of visual aids or ANC cards during the consultation, and gestational age at ANC visit were positively associated with quality nutrition service at ANC. In the adjusted analysis, there was an association between facilities with ~~medium~~ average and good logistical readiness with higher quality nutrition service provision (~~adjusted incidence rate ratio,~~ aIRR: 1.17, 95% CI: 1.04-1.32 and aIRR: 1.23, 95% CI: 1.08-1.39, respectively) compared to facilities with low readiness. Although nurses provided services ~~with~~ of similar quality to the physicians, paramedics were 23% (aIRR: 1.23, 95% CI: 1.06-1.42) and community health care providers were 32% (aIRR: 1.32, 95% CI: 1.12-1.57) more likely to provide quality nutrition services. Each point increase in ~~the~~ HCPs’ knowledge of nutrition was associated with a 4% higher quality of nutrition

services at ANC (aIRR: 1.04; 95% CI: 1.01-1.08). ~~-~~There was no association between HCP~~'~~s' training on nutrition and receiving external supervision in the last six months with quality nutrition service provision in the adjusted analysis. There was an association between HCP-client communication (aIRR: 1.14; 95% CI: 1.04-1.26) and the use of visual aids or ANC cards (aIRR: 1.18; 95% CI: 1.11-1.27) with quality nutrition service provision. Finally, the client~~'~~s in the second and third trimesters of pregnancy received higher-quality nutrition service (aIRR: 1.23, 95% CI: 1.11-1.35 and aIRR: 1.19, 95% CI: 1.08-1.32, respectively) compared ~~with~~ to those in the first trimester.

Table 3. Unadjusted and adjusted incidence rate ratio of quality nutrition service at ANC according to health facility readiness, HCP characteristics, client-provider interactions and client characteristics

Variables	Unadjusted models		Adjusted model	
	**IRR (95% CI)**	**p-value**	**aIRR (95% CI)**	**p-value**
**Facility characteristics and readiness**				
All structural readiness[1] available	0.86 (0.77-0.96)	0.007	1.00 (0.98-1.03)	0.884
Logistical readiness quantile[2] (ref: Level 1 (poor~~low~~))		0.015		0.013
Level 2 (average~~medium~~)	1.23 (1.04-1.45)	0.016	1.17 (1.04-1.32)	0.011
Level 3 (good)	1.31 (1.11-1.54)	0.001	1.23 (1.08-1.39)	0.002
Level 4 (best)	1.27 (1.03-1.58)	0.024	1.10 (0.95-1.28)	0.201
Average number of ANC services provided ~~utilization~~ per day (ref: <5)		0.107		0.302
5-10	0.92 (0.81-1.05)	0.220	0.98 (0.89-1.07)	0.614
≥11 ~~or more~~	0.85 (0.73-0.99)	0.039	0.91 (0.82-1.02)	0.123
**Provider characteristics**				
Age of provider (years)	1.00 (0.99-1.00)	0.447		
Sex of provider (ref: female)	0.99 (0.84-1.17)	0.919		
Duration of work at this facility (years) (ref: ≤3)		0.062		0.731
4-7	1.16 (1.02-1.32)	0.024	1.04 (0.93-1.16)	0.537
≥8 ~~or more~~	1.13 (1.00-1.27)	0.055	1.05 (0.93-1.18)	0.464
Type of provider (ref: ~~P~~physician)		<0.001		<0.001
Nurse	0.99 (0.80-1.21)	0.900	0.89 (0.74-1.05)	0.169
Paramedic	1.41 (1.17-1.69)	<0.001	1.23 (1.06-1.42)	0.006
CHCP/CHW	1.46 (1.21-1.76)	<0.001	1.32 (1.12-1.57)	0.001
Received any training on nutrition	1.11 (1.00-1.24)	0.060	1.00 (0.91-1.10)	0.949
Knowledge on nutrition service (score out of 6)	1.07 (1.03-1.11)	0.001	1.04 (1.01-1.08)	0.012
Received external supervision in last 6 months	1.17 (1.01-1.36)	0.034	1.09 (0.97-1.24)	0.154
**HCP-client interaction (process)**				
HCP-client communication[3]	1.24 (1.11-1.38)	<0.001	1.14 (1.04-1.26)	0.007
Used visual aids or ANC card	1.19 (1.10-1.28)	<0.001	1.18 (1.11-1.27)	<0.001
**Client characteristics**				

Variables	Unadjusted models		Adjusted model	
	IRR (95% CI)	p-value	aIRR (95% CI)	p-value
Age of women (~~in~~ years)	1.00 (0.99-1.00)	0.169	1.00 (0.99-1.01)	0.946
Education (years of schooling)	1.00 (0.99-1.01)	0.781		
Gravida (ref: 1)		0.184		0.188
2	1.01 (0.96-1.05)	0.784	1.00 (0.96-1.05)	0.841
≥3 ~~or more~~	0.95 (0.90-1.01)	0.127	0.94 (0.87-1.02)	0.149
Gestational a~~A~~ge (ref: 1st trimester)		<0.001		<0.001
2nd trimester	1.28 (1.16-1.42)	<0.001	1.23 (1.11-1.35)	<0.001
3rd t~~T~~rimester	1.23 (1.10-1.37)	<0.001	1.19 (1.08-1.32)	0.001
Number of ANC v~~V~~isits (ref: 1st visit)		0.234		
2nd – 3rd	1.05 (0.99-1.11)	0.108		
≥4th ~~or more~~	1.04 (0.99-1.09)	0.156		

[1] Structural readiness: availability of examination room, privacy, examination bed, electricity and functioning toilet for clients; [2] Logistical readiness quantile was derived by categorizing score from principal component analysis of the availability of weighing scale, haemoglobin testing tool, health education materials/visual aids, ANC card, ANC guideline, ANC register and IFA supplement; [3] ~~-~~HCP discussing ~~about~~ the progress of pregnancy, asked if the client had any question; IRR: incidence rate ratio, aIRR: adjusted incidence rate ratio, ~~;~~ ANC: antenatal care, CHCP: c~~C~~ommunity h~~H~~ealth c~~C~~are p~~P~~roviders, CHW: c~~C~~ommunity h~~H~~ealth w~~W~~orkers; HCP: h~~H~~ealth care provider, ref: reference

We also explored facility, HCP and client-~~-~~level factors~~determinants~~ influencing ~~for~~ each of the components of nutrition services at ANC (Table 4 and Table 5). The key ~~determinants~~ influencing factors for weight measurement were the logistical readiness of the health facilities, clients seeing a lower-~~-~~level provider~~s~~ like a paramedic~~s~~ or CHCP/CHW, using visual aids or ANC cards during service provision, and clients attending health facilities in the second and third trimester (Table 4). Anaemia assessment was also positively associated with higher HCPs' knowledge on nutrition and the use of visual aids or ANC cards but inversely associated with providers receiving external supervision. For IFA provision, the key influencing factors~~determinants~~ included lower-~~-~~level HCPs like CHWs~~community health workers~~, clients in the second and third trimester of pregnancy, external supervision in the last six months, and HCP-client communication (Table 5). Provision of nutrition counselling was lower at facilities with 11 or more ANC services provided~~utilization~~ daily, but higher among community-~~-~~level providers and HCPs with better knowledge of nutrition services. The use of visual aids or ANC cards and good HCP-client communication during the consultation also resulted in ~~the~~ ~~higher~~ greater provision of nutrition counselling at ANC.

Table 4. Adjusted incidence rate ratio of ~~four nutrition service components~~ weight measurement and anaemia assessment at ANC according to health facility readiness, HCP characteristics, client-provider interactions and client characteristics

Variables	Weight measured		Anaemia[1] assessed	
	aIRR (95% CI)	p-value	aIRR (95% CI)	p-value
**Facility characteristics**				
All structural readiness[1] available	1.00 (0.97-1.04)	0.876	1.03 (0.96-1.10)	0.483
Logistical readiness quantile[3] (ref: Level 1 - poor~~low~~)		0.002		0.386
Level 2 (average~~medium~~)	1.61 (1.22-2.12)	0.001	0.94 (0.67-1.33)	0.749
Level 3 (good)	1.59 (1.21-2.11)	0.001	1.18 (0.87-1.60)	0.276
Level 4 (best)	1.39 (1.03-1.87)	0.030	0.94 (0.63-1.40)	0.761
Average number of ANC ~~utilization~~services provided per day (ref: <5)		0.699		0.163
5-10	1.00 (0.86-1.16)	0.999	1.06 (0.81-1.38)	0.668
≥11 ~~or more~~	1.06 (0.92-1.23)	0.456	0.78 (0.57-1.07)	0.130
**Provider characteristics**				
Duration of work in facility (years) (ref: ≤3)		0.486		0.334
4-7	1.07 (0.90-1.28)	0.429	1.17 (0.87-1.57)	0.298
≥8 ~~or more~~	1.11 (0.94-1.30)	0.212	0.95 (0.73-1.24)	0.712
Type of provider (ref: Physician)		0.001		0.132
Nurse	0.94 (0.76-1.18)	0.615	0.77 (0.51-1.17)	0.222
Paramedic	1.23 (1.01-1.49)	0.042	1.07 (0.80-1.43)	0.658
CHCP/CHW	1.32 (0.99-1.74)	0.055	0.76 (0.50-1.16)	0.202
Received training on nutrition	0.95 (0.85-1.06)	0.327	1.02 (0.76-1.37)	0.882
Knowledge on nutrition service (score out of 6)	0.98 (0.93-1.03)	0.404	1.18 (1.08-1.29)	<0.001
Received external supervision in last 6 months	1.00 (0.86-1.17)	0.959	0.77 (0.60-0.99)	0.042
**HCP-client interaction (process)**				
HCP-client communication[4]	1.06 (0.92-1.20)	0.427	0.87 (0.69-1.09)	0.222
Used visual aids or ANC card	1.23 (1.11-1.37)	<0.001	1.28 (1.08-1.53)	0.005
**Client characteristics**				
Age of women (~~in~~ years)	1.01(1.00-1.01)	0.144	1.00 (0.98-1.01)	0.546
Gravida (ref: 1)		0.084		0.927
2	1.02 (0.96-1.08)	0.546	0.99 (0.88-1.13)	0.907
≥3~~+~~	0.92 (0.82-1.02)	0.120	1.03 (0.87-1.20)	0.790
Gestational a~~A~~ge (ref: 1st trimester)		0.026		0.128
2nd trimester	1.19 (1.05-1.33)	0.005	1.03 (0.85-1.25)	0.763
3rd t~~T~~rimester	1.19 (1.04-1.35)	0.009	1.14 (0.93-1.38)	0.206

[1]Anaemia assessment included HCPs assessing ~~ment of~~ blood haemoglobin levels or examining~~ed~~ eye or palm (clinical assessment);~~,~~ [2] Structural readiness: availability of examination room/area, privacy, examination bed, electricity and functioning toilet for clients; [3]Logistical readiness quantile was derived by categorizing score from principal component analysis of the availability of weighing scale, haemoglobin testing tool, health education materials/visual aids, ANC card, ANC guideline, ANC register and IFA supplement;~~.~~ [4] ~~-~~HCP discussing ~~about~~ the progress of pregnancy, asked if the client had any question;~~,~~ CHCP: c~~C~~ommunity h~~H~~ealth c~~C~~are p~~P~~roviders, CHW: c~~C~~ommunity h~~H~~ealth w~~W~~orkers; HCP: h~~H~~ealth care provider, aIRR: adjusted incidence rate ratio

Table 5. Adjusted incidence rate ratio of IFA provision and nutrition counselling at ANC according to health facility readiness, HCP characteristics, client-provider interactions and client characteristics.

Variables	IFA provided		Nutrition counselling	
	aIRR (95% CI)	p-value	aIRR (95% CI)	p-value
**Facility characteristics**				
All structural readiness[1] available	0.97 (0.91-1.04)	0.383	1.00 (0.94-1.06)	0.996
Logistical readiness quantile[3] (ref: Level 1 - poor~~low~~)		0.410		0.236
Level 2 (average~~medium~~)	0.83 (0.61-1.13)	0.253	1.29 (0.96-1.75)	0.095
Level 3 (good)	0.81 (0.62-1.05)	0.108	1.35 (1.00-1.82)	0.047
Level 4 (best)	0.84 (0.61-1.17)	0.306	1.25 (0.90-1.74)	0.190
Average number of ANC ~~utilization~~services provided per day (ref: <5)		0.799		0.045
5-10	0.93 (0.72-1.18)	0.538	0.89 (0.72-1.10)	0.271
≥11 ~~or more~~	1.01 (0.73-1.39)	0.950	0.70 (0.53-0.94)	0.016
**Provider characteristics**				
Duration of work in facility (years) (ref: ≤3)		0.454		0.471
4-7	0.81 (0.58-1.14)	0.235	1.15 (0.88-1.51)	0.302
≥8 ~~or more~~	0.94 (0.68-1.33)	0.779	1.18 (0.89-1.56)	0.260
Type of provider (ref: Physician)		0.023		0.029
Nurse	0.87 (0.51-1.46)	0.594	0.88 (0.57-1.38)	0.586
Paramedic	1.41 (0.99-2.00)	0.058	1.28 (0.89-1.85)	0.182
CHCP/CHW	1.74 (1.19-2.56)	0.005	1.56 (1.03-2.34)	0.034
Received training on nutrition	1.11(0.88-1.40)	0.357	1.01(0.85-1.20)	0.886
Knowledge on nutrition service (score out of 6)	0.99 (0.91-1.07)	0.745	1.08(1.01-1.16)	0.040
Received external supervision in last 6 months	2.42(1.42-4.11)	0.001	1.00(0.75-1.31)	0.949
**HCP-client interaction (process)**				
HCP-client communication[4]	1.34 (1.06-1.70)	0.013	1.51 (1.17-1.95)	0.002
Used visual aids or ANC card	1.05 (0.90-1.22)	0.571	1.20 (1.01-1.42)	0.038
**Client characteristics**				
Age of women (~~in~~ years)	1.00(0.99-1.01)	0.955	1.00(0.98-1.01)	0.675
Gravida (ref: 1)		0.239		0.432
2	1.06 (0.96-1.15)	0.245	0.96 (0.87-1.06)	0.443
≥3~~+~~	0.96 (0.84-1.11)	0.599	0.90 (0.76-1.06)	0.219
Gestational Age (ref: 1st trimester)		<0.001		0.360
2nd trimester	2.13 (1.58-2.88)	<0.001	0.92 (0.79-1.09)	0.354
3rd t~~T~~rimester	1.84 (1.35-2.51)	<0.001	0.89 (0.75-1.05)	0.164

[1]Anaemia assessment included HCPs assessing blood haemoglobin levels or examining eye or palm (clinical assessment); [2] Structural readiness: availability of examination room/area, privacy, examination bed, electricity and functioning toilet for clients; [3]Logistical readiness quantile was derived by categorizing score from principal component analysis of the availability of weighing scale, haemoglobin testing tool, health education materials/visual aids, ANC card, ANC guideline, ANC register and IFA supplement; [4]HCP discussing the progress of pregnancy, asked if the client had any question; CHCP: community health care providers, CHW: community health workers; HCP: health care provider, aIRR: adjusted incidence rate ratio

Discussion

~~Antenatal care~~ANC is the formal point of contact for pregnant women to receive preventive health and nutrition services. We found that only 15% of women received all four nutrition services at ANC. Anaemia measurement and nutrition counselling were the least provided (52%) nutrition service. Logistical readiness of the facilities, types of ANC provider, HCPs' knowledge of nutrition services, client-provider interactions during the consultation~~,~~ and gestational age of clients were the factors influencing~~determinants of~~ quality nutrition services during ANC. We found limited associations of training and external supervision of the providers with the quality of nutrition services. Our study adds to the knowledge ~~on~~ about the quality of nutrition services ~~provision~~ provided during pregnancy. It provides some critical insights on areas to intervene for improving the nutrition service provision at ANC in a low-resource setting.

Our findings of the quality of nutrition service provision at ANC is consistent with the poor content of ANC services reported in Bangladesh and other low-income settings [11, 17, 20]. However, previous studies mostly focused on~~explored~~ the content of ANC, ~~based on the recall by~~ asking women ~~with~~who had ~~a~~ recently given birth if they had received any of the ~~listed~~specified services at any point ~~time~~ during their pregnancy, rather~~but~~ than~~not~~ on a~~at~~ ~~any~~ specific service cont~~r~~act [11, 15, 20]. ~~Point of care o~~Studies from Peru and Tanzania observing~~ation-based studies~~ ANC service at the point-of-care ~~from Peru and Tanzania~~ reported low adherence to recommended service provision ~~at ANC~~ [19, 21]. A~~n earlier~~ study in Bangladesh also reported ~~a~~ high ~~level of~~ weight measurement and IFA provision but inadequate ~~nutrition~~ counselling on nutrition at ANC [13].

Adequate readiness of the health facilities is essential to ensure quality~~any~~ service provision [11, 33-35]. Our study showed that improved logistical readiness of ~~the~~ health facilities is strongly associated with ~~a~~ higher quality ~~of~~ nutrition services provision at ANC. In the di~~e~~sag~~e~~gregated analysis, we found weight

assessmentss was substantially lower in the facilities with poor logistical readiness compared withto the service at facilities with better logistical readinessequipped facilities. However, the provision of IFA did not differ significantly by the facilities' logistical readiness levels of the facilities as IFA was available in most– (93%) of the facilities. –Although unadjusted analysis showed an inverse association between infrastructural readiness of facilities and quality nutrition service provision, this association wasis attenuated and no longer significant in the adjusted analysis. The crude association waiis likely to be confounded by the higher-tier facilities with better infrastructural readiness but poorer service provision, as shown in our descriptive analysis. Moreover, we found better quality service provision by lower-level providers who predominately mainly offer services at lower-tier facilities provided better quality service than physicians and nurses at higher-tier facilities. Previous studies from LMICs also reported that service provision by lower-level providers had offered similar or better compliance withto standard maternal and child out-patient care than providers at the higher-level facilitiesy [22, 36]. The National Nutrition Services NNS programme should ensure a consistent supply of the logistics and supplies that are essential for nutrition service provision at ANC to improve the quality of service. There should be an emphasis on role clarification of the providers at the higher level facility should be clarified based on WHO and national recommendations forof nutrition services during pregnancy.

Our study demonstrates that HCP-client interactions such aslike informing them about the progress of pregnancy and giving them the opportunity to ask questions had a significant association with the quality of nutrition service provision. BothThe WHO's standards for maternal and newborn care and WHO's guidelines on ANC recommend treating clients with dignity and compassion, and providing information on health status and available potential interventions for making an informed choice by clients [9, 37]. Earlier studies also reported the importance of HCP's' interpersonal communication and attitude towards clients- to improve the quality of ANC services provision at health facilities [16, 18]. A study in Laos reported , based on both HCP and client's opinion, that HCP's behaviour, mutual communication and

treating clients ~~equally~~ with dignity are important considerations to improve ANC service quality [16]. ~~All~~ T~~t~~his evidence highlights the importance of ~~enhancing client~~ client-provider interactions for improving the quality of ANC nutrition services. ~~These enhancements should include providing client's adequate information and treating them with dignity so that they feel empowered and confident to ask questions.~~

We also found that the application of job aid~~ tool~~s ~~like~~such as visual aids and ~~the~~ ANC cards during ANC consultations improved the quality of nutrition services, especially for~~on~~ weight measurement, anaemia assessment, and nutrition counselling. A possible reason could be ~~the requirement~~ that ~~the~~ HCPs document the key components of nutrition services provided on the ANC card, which acts as a reminder to ensure providing the services [38]. Similarly~~ to the ANC card~~, visual aids ~~are~~ likely ~~to act as a prompt to~~ remind HCPs of the counselling contents, improved communication techniques and service provision [39]. However, ~~T~~there was no association between the use of ANC cards or visual aids and increased provision of IFA supplements~~ation~~. The ANC card used in public facilities in Bangladesh does not ~~include any item on~~mention IFA provision. Moreover, IFA provision is more likely to depend ~~more~~ on the availability of the supplements, ~~the~~ HCP~~'~~s perceptions about supplementation, and client characteristics.

In contrast to previous studies, we did not find any association between~~of~~ HCP~~'~~s nutrition training ~~with~~and the quality of nutrition service delivery during ANC [21, 40]. The content and quality of their training in nutrition might explain this finding. There are several criticisms of the government's Basic Nutrition Training ~~received by~~ that most ~~of the~~ HCPs received. ~~-~~There was too much content to cover in a short training time[41]. It was overly focused ~~too much~~ on child feeding~~,~~ with~~and there were,~~ few practical demonstrations. ~~and~~ T~~t~~he training ~~lost~~ quality declined when it was scaled up ~~implemented~~ to a large number of HCPs ~~throughout the country~~nationwide using a cascade model [41, 42]. Only IFA provision was positively associated with r~~R~~eceiving external supervision ~~was positively associated only with IFA provision only~~. Like training on nutrition, the quality of supervision visits to HCPs is often a question in

resource-poor settings. HCPs often receive external supervision from Aadministrative supervisors often do the external supervision received by HCPs withoutwho —haveing muchlimited technical expertiseknowledge and supervisory skills, which resultings in a missed opportunity to reinforce the implementation of the knowledge and training obtained by HCPs into practice [41, 43]. Considering the quality issue of nutrition training, in 2017, the current NNS operation plan introduced Comprehensive Competency-Based Training on Nutrition in 2017, which is implemented by technical experts [10]. The Competency-Based Training also addressed the supervision issue by developing and training supervisors on specifically designed supervisor modules to improve the technical competence of the supervisors. However, the success ofwe do not know if the Competency-Based Training in overcoming these challenges is yet to be evaluated.

Gestation was an important- factordeterminant influencingof receiving thehigher quality of nutrition services during ANC. Women coming for ANC in late trimesters were more likely to received better quality nutrition services compared to women presenting in the first trimester [13, 22, 44]. The women presenting in the first trimester were less likely to have their weight assessed or receive IFA supplements. Low IFA supplement provision in the first trimester could be related to some inconsistent perceptions among HCPs on when tothe timing of starting IFA and the adverse effect of iron on foetal development [45, 46]. HCP's perception of the importance of different services and self-prioritisation of some services may also differ according to clients'the gestational age. A study in Ghana suggested that HCPs sometimes disregardconsider physical measurements not important in the first trimester [22]. However, the reasons for lower compliance with weight assessment in the earlier stage of pregnancy require further in-depth research. Detection and management of anaemia and provision of IFA early in pregnancy are important to avert avoid its anaemia's detrimental effect on maternal and child health outcomes [47]. Adaptation of the national ANC guideline to comply with the global recommendations and to improvinge HCPs'

~~perceptions~~awareness of~~ the HCPs on~~ the recommended nutrition services during~~at~~ ANC visits may improve~~enhance~~ the quality of services~~ provision~~.

The main strength of our study is the collection of observation-based data on nutrition service~~s~~ provision during~~at~~ ANC and the assessment of~~ the~~ facility, provider~~,~~ and client characteristics at the same time-point, which allowed us to undertake~~conduct the~~ supply-side and client-level factor~~determinant~~ analysis. The sampling of facilities covered 21 out of 64 districts spread across all geographic regions, which improved the generalis~~z~~ability of the study findings to public facilities in Bangladesh. Our study has some limitations. First~~ly~~, the observation of service provision by an external assessor may have resulted in some improved practice by HCPs. However, we anticipate that such bias will have a limited effect~~ of such bias~~ on the~~ determinant~~ analysis of factors influencing the quality of service as facility and HCPs~~provider~~ characteristics will~~ould~~ not change during the observation. To reduce the Hawthorne effect~~Besides~~, we conducted the observations in a natural setting, spending a reasonable time (~~,~~ i.e., one~~1~~-two~~2~~ full days)~~,~~ at each facility [48]. ~~and~~We assured~~ing the~~ HCPs about their anonymity when presenting findings and~~.~~ ~~Also, we~~ emphasised that our observations were not related to individual performance evaluation~~assessment to try and reduce the Hawthorne effect [48]~~. Second~~ly~~, the study methodology did not involve any in-depth interviews with HCPs to explore the reasons for the relatively low proportion of pregnant women whose ~~compliance to~~ weight was assessed~~ment~~ ~~in the women~~ during ~~presenting in~~ the first trimester of pregnancy and the limited impact of training and supervision on quality service provision. Third~~ly~~, we ~~could~~ did not~~ also~~ account for hospital management and governance level factors~~determinants~~ influencing the~~of~~ ~~providing~~ quality of nutrition service provision at health care platforms ~~like~~ such as ANC. Fourth, we assessed only the on-site availability of rapid haemoglobin testing kits at the ANC room, which may underestimate the haemoglobin testing readiness of district and sub-district hospitals as some of them might have advanced diagnostic tests for anaemia. Future research should explore these questions.

Conclusion

ANC is the primary~~main~~ platform to deliver nutrition services during pregnancy. The quality of nutrition service provided at ANC in public health facilities in Bangladesh is sub-standard. On the contrary to WHO and national recommendations, we observed differential practice in nutrition service provision by gestational age of the clients and type of HCPs proving the service~~, which~~ necessitating~~es~~ increased~~improving~~ awareness of ANC standards and guidelines among HCPs. Logistical readiness of the facility influences the quality of nutrition service provision and demand~~require~~s urgent attention from~~by the~~ nutrition program managers. Better HCP-client communications, ~~like~~such as providing the ANC clients with information about the progress of pregnancy and allowing them to ask questions were associated with~~–~~ improved~~ the~~ quality of the service. ~~Future HCP's capacity building and quality-focused supportive supervision should emphasize these key elements of service provision .~~The utilis~~z~~ation of simple but effective~~useful~~ tools, such as~~like~~ ANC cards and visual aids,~~ needed across all ANC services to~~ resulted in ~~improve~~ better~~the quality of~~ nutrition services and should be promoted universally.~~ Redesigning the content and implementation modality of HCP's training and assessing the long-term effect of such training in improving HCP's practice is imperative.~~ Failure of~~Lastly,~~ the traditional hierarchical administrative-focused supervision ~~failed~~ to improve the quality of nutrition service provision~~, which~~ calls for ~~a paradigm shift to~~ service-specific supportive supervision by technical experts to harness the benefit of supervision.

Acknowledgements

We thank the Directorate General Health Services, Directorate General Family Planning and National Nutrition Services of the Ministry of Health and Family Welfare for approving us to conduct the study at public health facilities. We acknowledge the contribution of physicians and research assistants involved

in the assessment. We are grateful to all participants for allowing us to conduct the assessment and interviews. We would like to thank Julie Ghostlaw, a program manager at IFPRI, for her support to edit the manuscript. icddr,b is also grateful to the Governments of Bangladesh, Canada, Sweden and the UK for providing core/unrestricted support.

References

1. Victora CG, Christian P, Vidaletti LP, Gatica-Domínguez G, Menon P, Black RE. Revisiting maternal and child undernutrition in low-income and middle-income countries: variable progress towards an unfinished agenda. The Lancet. 2021.

2. Christian P, Mullany LC, Hurley KM, Katz J, Black RE. Nutrition and maternal, neonatal, and child health. Seminars in perinatology. 2015;39(5):361-72.

3. National Institute of Population Research and Training (NIPORT), Mitra and Associates, and ICF International. Bangladesh Demographic and Health Survey 2014. Dhaka, Bangladesh, and Rockville, Maryland, USA: NIPORT, Mitra and Associates, and ICF International, 2016.

4. National Institute of Population Research and Training (NIPORT), Mitra and Associates, ICF. Bangladesh Demographic and Health Survey 2017-18: Key Indicators. Dhaka, Bangladesh, and Rockville, Maryland, USA: National Institute of Population Research and Training (NIPORT), ICF, 2019.

5. Arsenault JE, Yakes EA, Islam MM, Hossain MB, Ahmed T, Hotz C, et al. Very low adequacy of micronutrient intakes by young children and women in rural Bangladesh is primarily explained by low food intake and limited diversity. The Journal of nutrition. 2013;143(2):197-203.

6. Abera SF, Kantelhardt EJ, Bezabih AM, Tsadik M, Lauvai J, Ejeta G, et al. What factors are associated with maternal undernutrition in eastern zone of Tigray, Ethiopia? Evidence for nutritional well-being of lactating mothers. BMC public health. 2020;20(1):1-12.

7. Kuhnt J, Vollmer S. Antenatal care services and its implications for vital and health outcomes of children: evidence from 193 surveys in 69 low-income and middle-income countries. BMJ open. 2017;7(11):e017122.

8. Haileslassie K, Mulugeta A, Girma M. Feeding practices, nutritional status and associated factors of lactating women in Samre Woreda, South Eastern Zone of Tigray, Ethiopia. Nutrition journal. 2013;12(1):1-11.

9. World Health Organization. WHO recommendations on antenatal care for a positive pregnancy experience: WHO; 2016.

10. GoB. 4th Health, Population and Nutrition Sector Programme (4th HPNSP), Operational Plan, National Nutrition Service (NNS) Institute of Public Health Nutrition, Directorate General of Health Service, Ministry of Health and Family Welfare, Government of the People's Republic of Bangladesh, 2017.

11. Islam MM, Masud MS. Determinants of frequency and contents of antenatal care visits in Bangladesh: Assessing the extent of compliance with the WHO recommendations. PloS one. 2018;13(9):e0204752.

12. Nguyen PH, Khương LQ, Pramanik P, Billah SM, Menon P, Piwoz E, et al. Effective coverage of nutrition interventions across the continuum of care in Bangladesh: insights from nationwide cross-sectional household and health facility surveys. BMJ open. 2021;11(1):e040109.

13. Billah SM, Saha KK, Khan ANS, Chowdhury AH, Garnett SP, Arifeen SE, et al. Quality of nutrition services in primary health care facilities: Implications for integrating nutrition into the health system in Bangladesh. PloS one. 2017;12(5):e0178121-e. doi: 10.1371/journal.pone.0178121. PubMed PMID: 28542530.

14. Donabedian A. The quality of care: How can it be assessed? JAMA. 1988;260(12):1743-8.

15. Afulani PA, Buback L, Essandoh F, Kinyua J, Kirumbi L, Cohen CR. Quality of antenatal care and associated factors in a rural county in Kenya: an assessment of service provision and experience dimensions. BMC health services research. 2019;19(1):1-16.

16. Phommachanh S, Essink DR, Jansen M, Broerse JE, Wright P, Mayxay M. Improvement of Quality of Antenatal Care (ANC) Service Provision at the Public Health Facilities in Lao PDR: Perspective and Experiences of Supply and Demand Sides. BMC pregnancy and childbirth. 2019;19(1):1-13.
17. Kanyangarara M, Munos MK, Walker N. Quality of antenatal care service provision in health facilities across sub–Saharan Africa: Evidence from nationally representative health facility assessments. Journal of global health. 2017;7(2).
18. Emiru AA, Alene G, Debelew GT. Women's satisfaction with the quality of antenatal care services rendered at public health facilities in Northwest Ethiopia: the application of partial proportional odds model. BMJ open. 2020;10(9):e037085.
19. Wynne SJ, Duarte R, de Wildt G, Meza G, Merriel A. The timing and quality of antenatal care received by women attending a primary care centre in Iquitos, Peru: A facility exit survey. PloS one. 2020;15(3):e0229852.
20. Singh L, Dubey R, Singh S, Goel R, Nair S, Singh P. Measuring quality of antenatal care: a secondary analysis of national survey data from India. BJOG: An International Journal of Obstetrics & Gynaecology. 2019;126:7-13.
21. Bintabara D, Nakamura K, Ntwenya J, Seino K, Mpondo BC. Adherence to standards of first-visit antenatal care among providers: A stratified analysis of Tanzanian facility-based survey for improving quality of antenatal care. PloS one. 2019;14(5):e0216520.
22. Amoakoh-Coleman M, Agyepong IA, Zuithoff NP, Kayode GA, Grobbee DE, Klipstein-Grobusch K, et al. Client factors affect provider adherence to clinical guidelines during first antenatal care. PLoS One. 2016;11(6):e0157542.
23. Goudet S, Murira Z, Torlesse H, Hatchard J, Busch-Hallen J. Effectiveness of programme approaches to improve the coverage of maternal nutrition interventions in South Asia. Maternal & child nutrition. 2018;14 Suppl 4(Suppl 4):e12699-e. doi: 10.1111/mcn.12699. PubMed PMID: 30499258.
24. Billah SM, Arifeen SE. Evaluation of GoB, UNICEF, CIFF initiative to facilitate and accelerate implementation of the Bangladesh National Nutrition Services Registry for International Development for Impact Evaluations (RIDIE), Available at: 10.23846/ridie0902016. Available from: https://ridie.3ieimpact.org/index.php?r=search/detailView&id=415.
25. Billah SM, Khan ANS, Raihana S, Rahman M, Salam SS, Arifeen SE, et al. Evaluation of Accelerating Implementation of the Bangladesh National Nutrition Services: Initial Assessment 2016. 2016.
26. Campbell SM, Roland MO, Buetow SA. Defining quality of care. Social science & medicine. 2000;51(11):1611-25.
27. Bruce J. Fundamental elements of the quality of care: a simple framework. Studies in family planning. 1990;21(2):61-91.
28. NIPORT, ACPR, ICF International. Bangladesh Health Facility Survey 2014. Dhaka, Bangladesh: National Institute of Population Research and Training (NIPORT), Associates for Community and Population Research (ACPR) and ICF International, 2016.
29. MOH&FW. Maternal Health Standard Operating Procedure (SOP) Volume-1. Bangladesh: Ministry of Health and Family Welfare, 2015.
30. NIPORT, Mitra and Associates, and ICF International. Bangladesh Demographic and Health Survey 2014. . Dhaka, Bangladesh, and Rockville, Maryland, USA:: National Institute of Population Research and Training (NIPORT), Mitra and Associates, and ICF International., 2016.
31. Owili PO, Muga MA, Mendez BR, Chen B. Quality of maternity care and its determinants along the continuum in Kenya: A structural equation modeling analysis. PloS one. 2017;12(5):e0177756.
32. Harris T, Yang Z, Hardin JW. Modeling underdispersed count data with generalized Poisson regression. The Stata Journal. 2012;12(4):736-47.

33. Mansur AM, Rezaul KM, Mahmudul HM. Quality of antenatal care in primary health care centers of bangladesh. Journal of family & reproductive health. 2014;8(4):175.
34. Rahman A, Nisha MK, Begum T, Ahmed S, Alam N, Anwar I. Trends, determinants and inequities of 4+ ANC utilisation in Bangladesh. Journal of Health, Population and Nutrition. 2017;36(1):2.
35. Stelfox HT, Straus SE. Measuring quality of care: considering measurement frameworks and needs assessment to guide quality indicator development. Journal of clinical epidemiology. 2013;66(12):1320-7.
36. Hoque DE, Arifeen SE, Rahman M, Chowdhury EK, Haque TM, Begum K, et al. Improving and sustaining quality of child health care through IMCI training and supervision: experience from rural Bangladesh. Health policy and planning. 2014;29(6):753-62.
37. World Health Organization. Standards for improving quality of maternal and newborn care in health facilities. Switzerland: WHO, 2016 9241511214.
38. Gross K, Schellenberg JA, Kessy F, Pfeiffer C, Obrist B. Antenatal care in practice: an exploratory study in antenatal care clinics in the Kilombero Valley, south-eastern Tanzania. BMC pregnancy and childbirth. 2011;11(1):1-11.
39. Jennings L, Yebadokpo AS, Affo J, Agbogbe M. Antenatal counseling in maternal and newborn care: use of job aids to improve health worker performance and maternal understanding in Benin. BMC pregnancy and childbirth. 2010;10(1):1-13.
40. Omer AM, Haile D, Shikur B, Macarayan ER, Hagos S. Effectiveness of a nutrition education and counselling training package on antenatal care: a cluster randomized controlled trial in Addis Ababa. Health policy and planning. 2020;35(Supplement_1):i65-i75. doi: 10.1093/heapol/czaa101. PubMed PMID: 33165586.
41. Saha KK, Billah M, Menon P, El Arifeen S, Mbuya NV. Bangladesh National Nutrition Services: Assessment of Implementation Status: World Bank Publications; 2015.
42. Ajeani J, Mangwi Ayiasi R, Tetui M, Ekirapa-Kiracho E, Namazzi G, Muhumuza Kananura R, et al. A cascade model of mentorship for frontline health workers in rural health facilities in Eastern Uganda: processes, achievements and lessons. Global health action. 2017;10(sup4):1345497.
43. Karuga RN, Mireku M, Muturi N, McCollum R, Vallieres F, Kumar M, et al. Supportive supervision of close-to-community providers of health care: Findings from action research conducted in two counties in Kenya. PloS one. 2019;14(5):e0216444.
44. Berehe TT, Modibia LM. Assessment of Quality of Antenatal Care Services and Its Determinant Factors in Public Health Facilities of Hossana Town, Hadiya Zone, Southern Ethiopia: A Longitudinal Study. Advances in Public Health. 2020;2020:NA-NA.
45. Alam A, Rasheed S, Khan NU, Sharmin T, Huda TM, Arifeen SE, et al. How can formative research inform the design of an iron-folic acid supplementation intervention starting in first trimester of pregnancy in Bangladesh? BMC public health. 2015;15(1):374.
46. Siekmans K, Roche M, Kung'u JK, Desrochers RE, De-Regil LMJM, nutrition c. Barriers and enablers for iron folic acid (IFA) supplementation in pregnant women. 2018;14:e12532.
47. Di Renzo GC, Spano F, Giardina I, Brillo E, Clerici G, Roura LC. Iron deficiency anemia in pregnancy. Women's Health. 2015;11(6):891-900.
48. Oswald D, Sherratt F, Smith S. Handling the Hawthorne effect: The challenges surrounding a participant observer. Review of social studies. 2014;1(1):53-73.

Supporting information

S1 Table. Definition of outcome and explanatory variables and data collection methods

Response to reviewers' comments

Reviewer #1:

1. The study has addressed an important public health issue for Bangladesh, and other LIMCs. However, one thing should be noted, the four factors which were finally revealed as independent determinants (logistical readiness of the facilities, HCP's knowledge on maternal nutrition, better HCP-client communication, and use visual aids or ANC card) are essential pre-requisite for delivering quality nutrition service. If the pre-requisites are not fulfilled, it is not possible to deliver quality service. So, in discussion and conclusion sections of the papers should focused on the other factor (service provider: Doctors/paramedics/CHCP) as it is not a prerequisite like other four factors. Considering these issues, the authors should revised the term 'Determinants' used in the Title and introduction section.

   Response: Our study hypothesizes that facility readiness, health care providers, the process of care, and client characteristics all contribute to the provision of high-quality nutrition services at antenatal care contact. We examined a variety of variables in each category (including provider types) and identified which of those variables are associated with (or may influence) high-quality service. We have revised our title and introduction, replacing the term "Determinants" by "Factors influencing". In the discussion, we have included practice variations among different types of providers and essential programmatic inputs to mitigate the practice gap (page 19, lines 354-362) and now have included them in the conclusion (page 23 lines 444-445).

Reviewer #2:

1. ANC is the classic best entry point for counseling expecting mothers on proper nutrition, danger signs of pregnancy and monitor the health of the mother and the fetus. The authors looked at a critical health system fitness to provide quality nutrition services for pregnant women. The research questions are relevant and the methodology is sound. The results are clearly described and the implications well discussed.
   Response: Thank you for your comments on our manuscript.

   Just one curious question. What did the authors think was the reason for the finding that the quality of nutrition services given during the 2nd and 3rd trimester was better than the one during the 1st trimester? I know that some misperception or misunderstanding from the provider side was mentioned. A bit more explanation would be great, if possible.
   Response: We have discussed some possible explanations in the discussion (page 21, lines 404-412). The difference in the quality of nutrition service across different trimesters of pregnancy is mainly due to the providers' perception gap and self-prioritisation of some services over others. For instance, among the four service components included in the quality of nutrition service during ANC, weight measurement and provision of IFA supplements were poorer in the 1st trimester compared to women in the 2nd and 3rd trimesters. Sometimes health care providers choose not to offer IFA in early pregnancy fearing iron's adverse effect on foetal development. Similarly, sometimes they disregard physical measurement and weight assessment during early pregnancy. It is unlikely that the client's demand or choice for service played a big role in this case, as clients often lack knowledge/perception of good quality care and what to anticipate from service providers when seeking health care in low and middle-income settings.

Reviewer #3:
This manuscript will be a useful addition to our knowledge of nutrition services provided during pregnancy, especially for ANC in Low and Low Middle Income Countries.
Response: Thank you for your encouraging comments on our manuscript.

1. I have a few clarification/suggestions that should be addressed;
   Lines# 23 to 25. Definition is not clear. What is being counted? Will weight measurements done twice and anemia measurements done two times in one woman across pregnancy be counted as equivalent to weight measurement done once and and anemia measure done three times in another women?
   Response: We counted the number of the selected four services provided at each ANC consultation as the 'quality nutrition service' indicator from observation of the ANC consultations. It was a cross-sectional study, and we observed ANC service consultation for a woman only once. To avoid confusion, we have revised the text as:
   "*We considered four essential nutrition services at each ANC contact including maternal weight measurement, anaemia assessment, nutrition counselling and iron-folic acid (IFA) supplement provision. We defined a composite 'quality nutrition service' outcome by counting the number of services (out of four) provided at each ANC from observation data*" (page 2, lines 23-26).

2. Lines# 64 and 65. Some information is missing. Rephrase the statement for clarity.
   Response: We have rephrased the sentence to improve clarity (page 4, lines 65-68).
   "*Another challenge is ensuring the quality of nutrition interventions provided at ANC contacts, without which programmes will not fully harness the benefits of these interventions on maternal and child nutrition outcomes. Moreover, a recent study reported incomplete readiness of healthcare facilities to provide nutrition interventions during ANC (51%).*"

3. Line#175. typo.
   Response: We have corrected the typo (page 10, line 203)

4. Table 1: Haemoglobin testing tool. Was it expected that sub-districts and community clinics will be better than district hospitals? Are they special programs/interventions that are being run by the health authority in Bangladesh that focus on resourcing rural and sub-district hospitals as compared to district hospitals? In most LMICS we see that district level hospitals are better equipped that rural clinics.
   Response: In general, districts and sub-district level facilities have better readiness than union-level health facilities and community clinics**.** However, the availability of logistics and tests to provide ANC service is expected to be similar across all tiers of facilities. One explanation of poorer on-site readiness of haemoglobin testing at the district and sub-district hospital is out of the Tallquist strips due to high client load and availability of other advanced diagnostic facilities at district and sub-district levels. Health care providers sometimes refer the clients to these diagnostic labs for haemoglobin testing rather than doing the rapid test onsite. We did not consider the availability of advanced diagnostics tests for anaemia outside of ANC consultation as HCPs can recommend the test but do not take a decision if the test is done afterward. However, we have included the following text in the limitation (pages 22-23, lines 437-439)
   "*Fourth, we assessed only the on-site availability of rapid haemoglobin testing kits at the ANC room which might under-estimate the haemoglobin testing capacities at district and sub-district hospitals as some of them may have advanced diagnostic tests for anaemia.*"

5. Table 1: What does "ANC utilization per day" mean?
   Response: ANC utilization per day means the average number of ANC services provided on a day at the facility. We have now clarified this in Table 1 (page 11)

6. What was the effect of clients (pregnant women) educational status on the outcome variables?
   Response: We did not find any association between the client's educational status and the quality of nutrition service provided at ANC. We have reported this in table 3. Education was not associated with any of the four selected nutrition services in the bivariate analyses and we did not include it in the multiple regression model presented in Table 4.

7. Figure 2 B: should the items add up to 100%? Currently it adds up 99%.
   Response: We have corrected the rounding error in Figure 2B.

Reviewer #4:

1. Methods
   Upon what basis districts were selected?
   Response: We have expanded the text on district selection as follows (page 5-6, lines 103-111*):*
   "We selected the districts based on comparability of population density, literacy rate, housing characteristics, people in the lowest wealth quintile, access to a safe drinking water source, electricity connection, improved sanitation, coverage of childhood immunization, ANC, skilled birth attendance, postnatal care and modern methods of family planning, under-five mortality rate and childhood stunting prevalence. We created a score for each district by principal component analysis of these variables. Then, we applied nearest neighbour matching of district's PCA score to match two intervention districts with one non-intervention district. Finally, we selected seven matched groups of districts (each having two AINNS intervention districts and one non-intervention), which had the minimum difference in the PCA score."

2. Line 109:Better to mention Upa Zilla as sub district is non existent
   Response: We have added upazila health complexes and mentioned sub-district hospitals in parenthesis in line 123 on page 6. However, we have used sub-district hospital throughout the paper for the ease of understanding of the global audience.

3. Please mention how anaemia was defined
   Response: We have defined anaemia screening as (page 8, lines 169-171):
   "Screening for anaemia included assessing blood haemoglobin level either using Tallquist paper onsite, by previous laboratory investigation or examining eye or palm (clinical assessment)".

4. Conclusion:
   Need to make brief depending upon objective
   Response: We have revised the conclusion and made it relevant only to the study objectives and findings (page 23, lines 445-454).

Reviewer #5:

General comments

1. This manuscript is very interesting having very important findings, but the paper required same professional proofreading to edit the entire paper for language, grammatical, spelling, and punctuations to improve the paper for publication. Once the authors are able to address the concerns appropriately, the manuscript can be published.
   Response: We have proof-read the manuscript and corrected typos and grammatical errors.

2. The four main target variables (maternal weight measurement, anaemia assessment, nutrition counselling and iron-folic acid (IFA) supplementation) for this study was not properly addressed and exhausted, need to elaborate more.
   Response: We have elaborated the definitions of four target variables for quality of nutrition service in lines 164-174 on page 8:
   "*We considered weight assessment performed if the HCPs took the weight of pregnant women using either a digital or analogue weighing scale". "Screening for anaemia included assessing blood haemoglobin level either using Tallquist paper onsite, by previous laboratory investigation or examining eye or palm (clinical assessment). Provision of nutrition counselling included HCPs providing messages on dietary diversity, quantity and types of nutritious food. We defined IFA provision as HCPs distributing IFA supplements during the ANC consultation; however, we did not include HCPs prescribing IFA to be bought from outside pharmacies*".

3. **Methods**
   Page 7, line 133 – 137 the paragraph "We conducted the assessment in 231 facilities, and in 184 facilities, we observed ANC services provided on the assessment days. We interviewed 217 health care providers who offered ANC services at the facilities on the day of the visit, and we observed 1296 ANC consultations. We excluded 54 observations due to missing information about the health care providers, the client's characteristics, or the exit interview" is not clear, need to be reconsidered or rephrased.
   Response: We have rephrased the text as (page 7-8, lines 149-157):
   "*We assessed the readiness to provide ANC services in 231 facilities (Fig 1). ANC services were not sought in 47 out of the 231 facilities on the assessment days; thus, we observed 1295 ANC service consultations at 184 facilities. We had complete background information from 201 HCPs who offered ANC services on the day of the visit. We excluded 53 observations from the analysis due to missing information on HCPs or the client characteristics. Finally, we included 1,242 observations of ANC service consultation provided by 201 service providers at 179 facilities in the analysis for this paper*".

4. **Result**
   Page 10 line 213 & 2014 the sentence "On average, clients who received ANC from the facilities were 23 years old and had eight years of schooling" is not clear, need more explanation?.
   Response: We have rephrased the text as (page 12, lines 241-242):
   "*Clients who received ANC from the facilities had a mean age of 23 years (SD±4.3 years) and had a mean eight years (SD±2.9 years) of schooling***".**

5. Page 14, table 4 are highly congested and it crosses one page requires rearrangement or division into two table or more.
   Response: We have divided Table 4 into two tables (Table 4 and Table 5).

6. **Discussion**
   Grammatical, spelling, and punctuations improvement is required in this section
   Response: We have corrected grammatical, spelling and punctuation errors.

7. **Overall comment**
   There are some areas need corrections; such as rephrasing, grammatical, spelling, and language. Kindly review issues raised and if possible get a trained English proof reader to edit the entire manuscript.
   Response: We have revised accordingly.
