# Supplementary material for: Factors influencing quality nutrition service provision at antenatal care contacts: Findings from a public health facility-based observational study in 21 districts of Bangladesh
Source: PLoS One. 2022 Jan 27;17(1):e0262867. doi: 10.1371/journal.pone.0262867 (PMC8794200; doi:10.1371/journal.pone.0262867)
Supplement: Attachment — Submitted filename: Response to Reviewers.docx [file pone.0262867.s005.docx]

**Response to reviewers' comments**

**Reviewer #1:**

An interesting and important modifiable and independent service related factor revealed in your results is the TYPE OF PROVIDER. In your abstract, you have mentioned it nicely as- Although nurses provided services of similar quality to the physicians, paramedics were 23% (aIRR: 1.23, 95% CI: 1.06-1.42) and community health care providers were 32% (aIRR: 1.32, 95% CI: 1.12-1.57) more likely to provide quality nutrition services. However, this factor has not been addressed at all in the discussion. As I have mentioned earlier (In the first review, this is the only important influencing factor/determinants and this need to be highlighted/explained in the DISCUSSION.

**Response:** We agree with the reviewer that the type of HCPs is an important factor influencing the quality of nutrition service. We have now highlighted this in the discussion and proposed some possible explanations in the following additional text (page 19, lines 339-351):

“*Lower-level providers who mainly offer services at lower-tier facilities provided a better quality of nutrition service than physicians and nurses at higher-tier facilities. Previous studies from LMICs including Bangladesh have also reported that lower-level providers had similar or better compliance with standard maternal and child out-patient care than higher-level providers [1, 2]. All physicians and nurses in our study provided services at the district and subdistrict hospitals where HCP shortage is a challenge [3]. These physicians and nurses often provide service under high workload pressure, are responsible for multiple tasks and manage patients with complications [4, 5]. In such situations, HCP often prioritise curative health services over preventive nutrition services such as counselling and weight assessments for weight gain monitoring [5]. We also found nutrition counselling was lower at facilities where a higher number of ANC services were provided, and this occurs mostly at district and subdistrict hospitals. The role of the higher-level providers should be clarified based on WHO and national recommendations for nutrition services during pregnancy. Further in-depth qualitative assessment is necessary to better understand provider attitudes and motivation factors influencing their provision of nutrition services*.”

**Reviewer #2:**

The study is useful. The findings are relevant. I re-confirm my opinion that the manuscript is clearly written, the methodology is sound and results are well discussed.

**Response**: We appreciate the positive comment from the reviewer.

**Reviewer #3:**

Go over the manuscript carefully to correct any grammatical issues and enhance ease of reading. For instance, you can summarize the conclusion to less than half the current length.

**Response:** We have checked the manuscript thoroughly and corrected grammatical errors and language. We have summarized the conclusion (Pages 22-23, lines 422-435).

**Reviewer #4:**

manuscript titled Factors influencing quality nutrition service provision at antenatal care contacts:
findings from a public health facility-based observational study in 21 districts of Bangladesh. I had reviewed first and made few comments. All comments were addressed properly. It is satisfactory.

Response: We thank the reviewer for the encouraging comment.

1.	Amoakoh-Coleman M, Agyepong IA, Zuithoff NP, Kayode GA, Grobbee DE, Klipstein-Grobusch K, et al. Client factors affect provider adherence to clinical guidelines during first antenatal care. PLoS One. 2016;11(6):e0157542.

2.	Hoque DE, Arifeen SE, Rahman M, Chowdhury EK, Haque TM, Begum K, et al. Improving and sustaining quality of child health care through IMCI training and supervision: experience from rural Bangladesh. Health Policy Plan. 2014;29(6):753-62.

3.	Organization WH. Bangladesh health system review: Manila: WHO Regional Office for the Western Pacific; 2015.

4.	Joarder T, Tune SNBK, Nuruzzaman M, Alam S, de Oliveira Cruz V, Zapata T. Assessment of staffing needs for physicians and nurses at Upazila health complexes in Bangladesh using WHO workload indicators of staffing need (WISN) method. BMJ Open. 2020;10(2):e035183.

5.	Saha KK, Billah M, Menon P, El Arifeen S, Mbuya NV. Bangladesh National Nutrition Services: Assessment of Implementation Status: World Bank Publications; 2015.
